# Trunk Biomechanics in Individuals with Knee Disorders: A Systematic Review with Evidence Gap Map and Meta-analysis

**DOI:** 10.1186/s40798-022-00536-6

**Published:** 2022-12-12

**Authors:** Marina C. Waiteman, Lionel Chia, Matheus H. M. Ducatti, David M. Bazett-Jones, Evangelos Pappas, Fábio M. de Azevedo, Ronaldo V. Briani

**Affiliations:** 1 Department of Physical Therapy, School of Science and Technology, Sao Paulo State University (UNESP), 305, Roberto Simonsen Street, Presidente Prudente, Sao Paulo 19060-900 Brazil; 2 Sydney School of Health Sciences, Faculty of Medicine and Health, The University of Sydney, Sydney, NSW Australia; 3 Cleveland Guardians Baseball Company, Cleveland, OH USA; 4 School of Exercise and Rehabilitation Sciences, College of Health and Human Services, University of Toledo, Toledo, OH USA; 5 School of Medicine and Illawarra Health and Medical Research Institute, The University of Wollongong, Wollongong, NSW Australia

**Keywords:** Trunk motion, Knee pain, Knee injuries, Knee surgeries, Anterior cruciate ligament

## Abstract

**Background:**

The trunk is the foundation for transfer and dissipation of forces throughout the lower extremity kinetic chain. Individuals with knee disorders may employ trunk biomechanical adaptations to accommodate forces at the knee or compensate for muscle weakness. This systematic review aimed to synthesize the literature comparing trunk biomechanics between individuals with knee disorders and injury-free controls.

**Methods:**

Five databases were searched from inception to January 2022. Observational studies comparing trunk kinematics or kinetics during weight-bearing tasks (e.g., stair negotiation, walking, running, landings) between individuals with knee disorders and controls were included. Meta-analyses for each knee disorder were performed. Outcome-level certainty was assessed using the Grading of Recommendations Assessment, Development, and Evaluation (GRADE), and evidence gap maps were created.

**Results:**

A total of 81 studies investigating trunk biomechanics across six different knee disorders were included (i.e., knee osteoarthritis [OA], total knee arthroplasty [TKA], patellofemoral pain [PFP], patellar tendinopathy [PT], anterior cruciate ligament deficiency [ACLD], and anterior cruciate ligament reconstruction [ACLR]). Individuals with knee OA presented greater trunk flexion during squatting (SMD 0.88, 95% CI 0.58–1.18) and stepping tasks (SMD 0.56, 95% CI 0.13–.99); ipsilateral and contralateral trunk lean during walking (SMD 1.36; 95% CI 0.60–2.11) and sit-to-stand (SMD 1.49; 95% CI 0.90–2.08), respectively. Greater trunk flexion during landing tasks in individuals with PFP (SMD 0.56; 95% CI 0.01–1.12) or ACLR (SMD 0.48; 95% CI 0.21–.75) and greater ipsilateral trunk lean during single-leg squat in individuals with PFP (SMD 1.01; 95% CI 0.33–1.70) were also identified. No alterations in trunk kinematics of individuals with TKA were identified. Evidence gap maps outlined the lack of investigations for individuals with PT or ACLD, as well as for trunk kinetics across knee disorders.

**Conclusion:**

Individuals with knee OA, PFP, or ACLR present with altered trunk kinematics in the sagittal and frontal planes. The findings of this review support the assessment of trunk biomechanics in these individuals in order to identify possible targets for rehabilitation and avoidance strategies.

Trial registration: PROSPERO registration number: CRD42019129257.

**Supplementary Information:**

The online version contains supplementary material available at 10.1186/s40798-022-00536-6.

## Key Points

1. Individuals with knee osteoarthritis, patellofemoral pain, or anterior cruciate ligament reconstruction present with altered trunk kinematics in the sagittal and frontal planes, while no trunk alterations were identified in individuals with total knee arthroplasty.
2. There is a noticeable lack of investigation of trunk kinematics and kinetics in individuals with patellar tendinopathy and anterior cruciate ligament deficiency.
3. Assessing trunk kinematics in clinical practice may help to identify possible targets for rehabilitation and avoidance strategies in individuals with osteoarthritis, patellofemoral pain, or anterior cruciate ligament reconstruction.

## Introduction

Knee disorders are very common [1–4] and are linked to prolonged recovery periods and higher reinjury rates [5–8]. Pain is reported to continue for 5–8 years after physical therapies for patellofemoral pain (PFP) in 1 of every 2 patients [5], while nearly 1 in 4 young athletic patients who sustain an anterior cruciate ligament (ACL) injury and return to high-risk sport will sustain another ACL injury at some point in their career [6]. To improve secondary and tertiary prevention efforts, a greater understanding of the effects of knee disorders on trunk biomechanics is required given its influence on knee loading.

The trunk accounts for 38% of whole-body center of mass position [9] and is the foundation for transfer and dissipation of forces throughout the lower extremity kinetic chain during weight-bearing tasks [10–12]. For example, landing from a jump with the trunk flexed was reported to result in 28% less quadriceps activation when compared to landing with the trunk more erect [13]. Conversely, hip extensor weakness is commonly reported in individuals with knee disorders [14–16], which would be expected to be compensated with a more erect trunk posture during dynamic activities [10]. Such trunk motion would increase the demand on the quadriceps and could have implications for several injuries at the knee, including patellar tendinopathy, PFP, and ACL strain (resulting from quadriceps-induced anterior shear forces acting on the tibiofemoral joint) [10]. In the frontal plane, increases in ipsilateral trunk lean (i.e., toward the stance limb) can reduce medial knee load in a dose–response manner [17]. Although this compensatory motion may be beneficial for individuals with medial knee osteoarthritis (OA) [17], ipsilateral trunk lean could create a valgus moment at the knee [10], which may be associated with ACL injury [18] and PFP [19]. A reduction of knee valgus has also been reported to be associated with decreased knee pain levels [20].

Previous systematic reviews have not synthesized the literature to identify trunk biomechanics in individuals with knee disorders. Such a review would inform researchers and clinicians on the utility of assessing trunk biomechanics in different types of knee disorders to identify possible targets for intervention. Therefore, the aims of this systematic review were to: (1) determine whether trunk biomechanics are altered in individuals with knee disorders compared to controls; (2) determine the level of the evidence certainty available; and (3) identify evidence gaps in the literature.

## Methods

This systematic review has been conducted and reported according to Preferred Reporting Items for Systematic Reviews and Meta-Analyses (PRISMA 2020) [21] and Prisma in Exercise, Rehabilitation, Sport medicine and SporTs science (PERSiST) guidelines [22]. The review protocol was pre-registered in PROSPERO (registration number: CRD42019129257).

### Data Sources and Search Strategy

A comprehensive literature search in Medline via OVID, Embase via OVID, CINAHL via EBSCOhost, SPORTDiscus via EBSCOhost, and Web of Science was performed from database inception to January 2022. The search strategy performed in Medline is presented in Additional file 1A. The electronic search was complemented by hand-searching the references of the retrieved studies and citation tracking of the original studies in Google Scholar.

### Selection Criteria

Observational cross-sectional and case–control studies comparing trunk biomechanics (kinematics or kinetics) during weight-bearing tasks (e.g., stair negotiation, walking, running, landings) between injury-free individuals (controls) and those with knee disorders were included. Knee disorders included were: knee OA, PFP, ACL deficiency [ACLD], and patellar tendinopathy [PT], total knee arthroplasty [TKA], and ACL reconstruction [ACLR]. As the prevalence of co-existing tibiofemoral and patellofemoral OA (40%) is higher than isolated tibiofemoral (4%) or patellofemoral (24%) OA [23], no compartment-specific OA was determined in the included studies (i.e., knee OA). Studies were excluded if they did not report a comparator group composed by controls, or investigated laboratory-induced tasks (e.g., balance provocations, sudden force release or external perturbation, laboratory-induced trips). Randomized controlled trials, cluster clinical trials, controlled (non-randomized) clinical trials, cadaveric studies, review papers, editorials, abstracts, and letters were not included. There was no restriction on participant sex or age, or year of publication. Language was limited to English, Portuguese or Spanish.

### Review Process

A single investigator (MCW) exported all studies identified by the electronic search to Covidence (Collins St, Melbourne, AU) where duplicates were removed. Titles, abstracts, and full-text screening were performed independently for eligibility by two reviewers (MCW and RVB). Any disagreements were resolved by consensus and the input of a third reviewer (MHMD).

### Methodological Quality Assessment

Methodological quality assessment was performed using a modified 14-item Downs and Black checklist [24, 25]. The modified Downs and Black checklist was used instead of the modified Newcastle–Ottawa scale as registered in PROSPERO because the former has been used in similar systematic reviews [26–28] and had good interrater reliability [29]. The domains considered not applicable to assess observational cross-sectional studies were removed resulting in a modified version of 14 domains [24, 30]. Studies were considered as ‘high quality’ (HQ) when scoring 11 or higher, ‘moderate quality’ (MQ) when scoring from 6 to 10 and ‘low quality’ (LQ) when scoring 5 or lower [28]. Assessments were performed independently by two reviewers (MCW and MHMD), and disagreements were resolved by consensus and the input of a third reviewer (RVB).

### Data Extraction

Study characteristics including publication details (e.g., author, year, study design), population characteristics (e.g., knee injury/surgery, number of participants, age, sex, body mass index), procedures (e.g., task, instrumentation), and outcomes were extracted and entered into Excel by one reviewer (MCW). All extracted data were double-checked by a second reviewer (MHMD). If necessary, authors were contacted via e-mail for further information to facilitate accurate data extraction. When possible, data reported as graphs were digitized and extracted using WebPlotDigitizer 4.0 (Ankit Rohatgi, San Francisco, CA).

### Data Analysis

Extracted data on trunk biomechanics were grouped according to population, measurement method (e.g., kinematics, kinetics), planes of motion (i.e., sagittal, frontal, or transverse planes), outcome (e.g., peak or range of motion [RoM], angle at initial contact [IC], or take-off), and task. Similar tasks were separated into categories: walking, running, stepping tasks (e.g., stair ascent, stair descent, step-down task), squatting tasks (e.g., single-leg squat, sit-to-stand), landing and jumping tasks. Outcomes were separately analyzed considering the task phase (e.g., IC, weight acceptance [peak/RoM], or take-off) or whether they were relative to another parameter (e.g., peak vertical ground reaction force) as they may represent different joint mechanics [31–34]. When a study reported outcomes that measured peak angle and RoM (which are similar underlying parameters), they were pooled together. When a study used a waveform analysis (e.g., statistical parametric mapping), qualitative synthesis was performed as data were unable to be pooled. Where two or more studies were available, data were pooled in a meta-analysis using a random effects model (Review Manager Version 5.3.). Standardized mean differences (SMD) and 95% confidence intervals (CIs) were calculated for all variables analyzed in the knee disorders versus control groups by dividing the difference between groups by the pooled SD. SMD were interpreted according to Cohen’s criteria: large effect defined as ≥ 0.8, moderate as > 0.5 and < 0.8, small effect as ≤ 0.5 and ≥ 0.2, and < 0.2 as no effect [35]. Statistical heterogeneity was quantified by the *I*^2^ statistic where *I*^2^ < 50% was considered not important, 50–75% as moderate, and > 75% as high heterogeneity [36]. Sensitivity analyses were performed by removing studies when there was high heterogeneity or where we used a digitizing software to extract data from graphs.

### Level of Certainty

Following the Cochrane recommendation [37], outcome-level certainty was assessed for all meta-analyses using the Grading of Recommendations Assessment, Development, and Evaluation (GRADE). A modified version proposed for observational studies was used as per recent publications [38–40]. The criteria adopted in this review comprise 4 domains: (1) risk of bias (i.e., more than 25% of participants from studies with a high risk of bias; studies scoring as low quality in the Downs and Black checklist were rated as high risk of bias), (2) inconsistency (i.e., substantial heterogeneity across the studies [− 1 for *I*^2^ > 50%; − 2 for *I*^2^ > 75%]), (3) imprecision (i.e., total sample size < 100 participants per group, large confidence intervals or confidence intervals that do not overlap), and (4) indirectness (i.e., study population and outcome measures do not align with the purpose of the review) (Table 1). Publication bias (i.e., visual inspection of funnel plot’s asymmetry or Egger’s test *P* < 0.10) was assessed when at least 10 studies were pooled [37, 38]. We downgraded one level for each domain not met from ‘high’ to ‘very low.’ Inconsistency was the only domain that could be double downgraded. We upgraded one level if more than 50% of pooled studies had a large effect (i.e., Cohen’s criteria ≥ 0.8). Levels of certainty were defined as follows:

1. High: Further research is very unlikely to change confidence in the estimate of the effect;
2. Moderate: Further research is likely to have an important impact on confidence in the estimate of the effect and may change the estimate;
3. Low: Further research is very likely to have an important impact on confidence in the estimate of the effect and is likely to change the estimate;
4. Very low: There is very little confidence in the effect estimate.

**Table 1:** Level of certainty evidence for each outcome from pooled data (GRADE approach)

Outcome	No of participants (studies)	SMD (95% CI)	Downgrading domains				Publication bias^‡‡^	Upgrading domain	Level of certainty
			RoB^*^	Inconsistency^†^	Imprecision^††^	Indirectness^‡^		Large effect	
*Knee OA*									
Trunk flexion (walking)	233 (4)	0.40 (− 0.05, 0.84)	0	− 1	0	0	–	0	Moderate
Trunk flexion (sit-to-stand)	402 (4)	0.88 (0.58, 1.18)	0	0	0	0	–	+ 1	High
Trunk flexion (stepping tasks)	95 (2)	0.56 (0.13, 0.99)	0	0	− 1	0	–	0	Moderate
Ipsilateral trunk lean (walking)	643 (8)	1.36 (0.60, 2.11)	0	− 2	0	0	–	+ 1	Moderate
Contralateral trunk lean (sit-to-stand)	227 (2)	1.49 (0.90, 2.08)	0	− 1	0	0	–	+ 1	High
Ipsilateral trunk rotation (walking)	208 (3)	0.00 (− 0.28, 0.28)	0	0	0	0	–	0	High
*TKA*									
Trunk flexion (walking)	95 (3)	0.09 (− 0.31, 0.50)	0	0	− 1	0	–	0	Moderate
Trunk flexion (stepping tasks)	116 (3)	− 0.15 (− 0.55, 0.25)	0	0	− 1	0	–	0	Moderate
Ipsilateral trunk lean (walking)	70 (2)	− 0.10 (− 0.57, 0.37)	0	0	− 1	0	–	0	Moderate
Contralateral trunk lean (walking)	70 (2)	0.06 (− 0.41, 0.53)	0	0	− 1	0	–	0	Moderate
Ipsilateral trunk lean (stepping tasks)	70 (2)	− 0.20 (− 0.67, 0.27)	0	0	− 1	0	–	0	Moderate
Contralateral trunk lean (stepping tasks)	70 (2)	− 0.07 (0.54, 0.40)	0	0	− 1	0	–	0	Moderate
Ipsilateral trunk rotation (walking)	70 (2)	0.52 (0.04, 0.99)	0	0	− 1	0	–	0	Moderate
Ipsilateral trunk rotation (stepping tasks)	70 (2)	0.00 (− 0.47, 0.47)	0	0	− 1	0	–	0	Moderate
*PFP*									
Trunk flexion (running)	126 (3)	0.32 (− 0.15, 0.79)	0	0	− 1	0	–	0	Moderate
Trunk flexion (stepping tasks)	143 (2)	0.01 (− 0.32, 0.34)	0	0	− 1	0	–	0	Moderate
Trunk flexion (landing tasks)	70 (2)	0.56 (0.01, 1.12)	0	0	− 1	0	–	0	Moderate
Ipsilateral trunk lean (running)	158 (4)	0.20 (− 0.12, 0.52)	0	0	− 1	0	–	0	Moderate
Ipsilateral trunk lean (squatting tasks)	174 (3)	1.01 (0.33, 1.70)	0	− 1	− 1	0	–	+ 1	Moderate
Ipsilateral trunk lean (stepping tasks)	120 (2)	0.39 (− 0.13, 0.91)	0	0	− 1	0	–	0	Moderate
Contralateral trunk lean (stepping tasks)	108 (2)	0.09 (− 0.29, 0.47)	0	0	− 1	0	–	0	Moderate
Ipsilateral trunk lean (landing tasks)	70 (2)	1.12 (− 1.22, 3.47)	0	− 2	− 2	0	–	0	Very low
Ipsilateral trunk rotation (landing tasks)	70 (2)	− 0.63 (− 1.86, 0.61)	0	− 2	− 1	0	–	0	Very low
*ACLR*									
Trunk flexion at IC (landing)	229 (3)	0.69 (0.38, 1.01)	0	0	− 1	0	–	0	Moderate
Trunk flexion (landing)	933 (13)	0.48 (0.21, 0.75)	0	− 1	0	0	0	0	Moderate
Trunk flexion (jumping)	312 (4)	− 0.76 (− 1.62, 0.10)	− 1	− 2	− 1	0	–	0	Very Low
Ipsilateral trunk lean at IC (landing)	180 (2)	− 0.01 (− 0.48, 0.46)	0	− 1	− 1	0	–	0	Low
Ipsilateral trunk lean (landing)	568 (6)	0.23 (− 0.05, 0.51)	0	− 1	0	0	–	0	Moderate

*ACLR* anterior cruciate ligament reconstruction, *IC* initial contact, *OA* osteoarthritis, *PFP* patellofemoral pain, *RoB* risk of bias, *SMD* standardized mean differences, *TKA* total knee arthroplasty

**Downgrading domains**

^*^More than 25% of participants from studies with a high risk of bias. Studies scoring as low quality in the Downs and Black checklist were rated as high risk of bias (− 1)

^†^Substantial heterogeneity across the studies (− 1 for *I*^2^ > 50%; − 2 for *I*^2^ > 75%)

^††^Total sample size < 100 participants per group, large confidence intervals or confidence intervals that do not overlap (− 1)

^**‡**^Study population and outcome measures align with the purpose of the review

^**‡‡**^Evidence of publication bias by asymmetry of the funnel plot or Egger’s test *P* < 0.10

**Upgrading domain**

Large effect: upgrade (+ 1) if more than 50% of pooled studies had Cohen’s criteria ≥ 0.8

### Evidence Gap Map

An evidence gap map was created to provide an overview of the evidence available investigating trunk biomechanics in individuals with knee disorders. The evidence gap map allows the identification of outcomes with sufficient or insufficient evidence due to the number of similar studies [41]. For the presentation of the evidence, all data were grouped according to measurement method, planes of motion, outcome, and task categories as previously mentioned. To assess levels of evidence synthetized in the evidence gap map, an updated version of van Tulder’s criteria was used as listed below [42]:

1. Strong evidence: provided by pooled results derived from three or more studies, including a minimum of two high-quality studies, which were statistically homogeneous (*P* > 0.05); may be associated with a statistically significant or non-significant pooled result.
2. Moderate evidence: provided by statistically significant pooled results derived from multiple studies that were statistically heterogeneous (*P* < 0.05), including at least one high-quality study; or from multiple low-quality studies, which were statistically homogeneous (*P* > 0.05).
3. Limited evidence: provided by results from one high-quality study or multiple low-quality studies that are statistically heterogeneous (*P* < 0.05).
4. Very limited evidence: provided by results from one low-quality study.
5. Conflicting evidence: provided by inconsistent findings among multiple trials and derived from multiple studies regardless of quality that are statistically heterogeneous.

## Results

### Search Results and Studies’ Characteristics

**Fig. 1:**
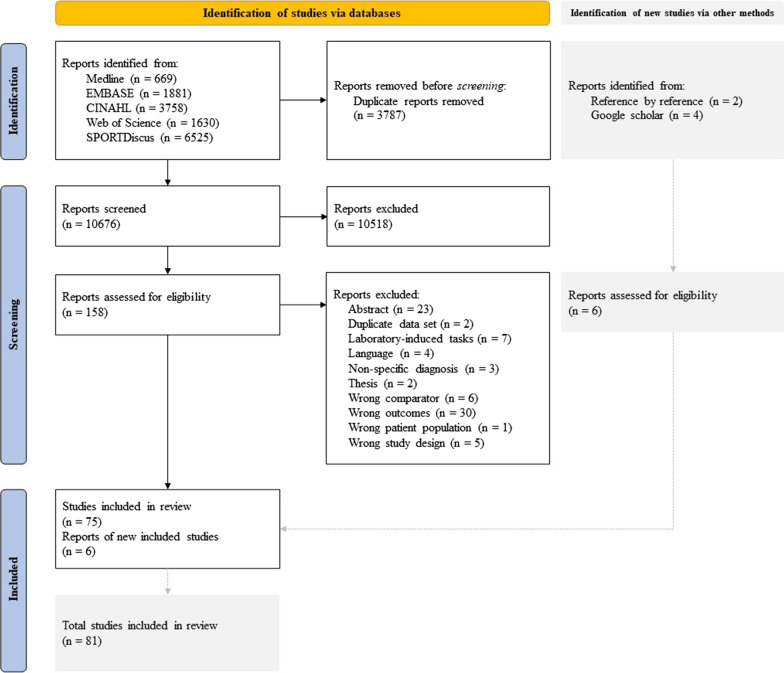
Flow diagram of search results. *CINAHL* Cumulative Index to Nursing and Allied Health Literature

The electronic database search yielded 14,463 studies initially (Fig. 1). Following the removal of duplicates, 10,676 studies were screened by title and abstracts for inclusion. Of these, 158 full-text studies were screened for inclusion and 83 studies did not meet eligibility criteria. Reasons for exclusion are presented in Additional file 1B. Six additional studies were also included based on hand-searching. Eighty-one studies were then included in this review.

From the 81 included studies, 26 studies investigated individuals with OA [43–68], 7 investigated individuals with TKA [69–75], 19 investigated individuals with PFP [76–94], 2 investigated individuals with PT [95, 96], 5 investigated individuals with ACLD [97–101], 19 investigated individuals with ACLR [34, 102–119], and 3 investigated more than 1 knee disorder (e.g., ACLD and ACLR, knee OA and TKA) [120–122].

Fifty-eight studies investigated three-dimensional trunk kinematics in the sagittal plane (knee OA [*n* = 16], TKA [*n* = 7], PFP [*n* = 13], PT [*n* = 2], ACLD [*n* = 3], ACLR [*n* = 17]), 54 in the frontal plane (knee OA [*n* = 21], TKA [*n* = 2], PFP [*n* = 15], ACLD [*n* = 3], ACLR [*n* = 13]), and 16 in the transverse plane (knee OA [*n* = 7], PFP [*n* = 4], ACLD [*n* = 1], ACLR [*n* = 4]). One study investigated trunk acceleration using triaxial accelerometers in individuals with ACLR [102], 1 study investigated trunk kinematics in the frontal plane using 3D accelerometers [64], and 1 study investigated trunk kinematics in the sagittal plane using a bi-axial accelerometer and a gyroscope in individuals with TKA [72]. One study investigated two-dimensional trunk sagittal plane displacement in individuals with PFP [82], and 2 studies investigated two-dimensional trunk sagittal and frontal planes displacement in individuals with ACLD [97, 99].

### Participant Characteristics

Studies included 894 individuals with knee OA [43–68, 121], 160 individuals with TKA [69–75, 121], 28 individuals with PT [95, 96], 442 individuals with PFP [76–94], 203 individuals with ACLD [97–101, 120, 122], 702 individuals with ACLR [34, 102–120, 122], and 1875 controls. The detailed characteristics of participants included in each study, their measurement methods, and outcomes are presented in Additional file 1C.

### Methodological Quality

Agreement between raters for the Downs and Black checklist was 88% with scores ranging from 4 to 12. Of the 81 studies, 22 were high quality, 56 were moderate quality, and 3 were low quality (Additional file 1D).

### Data Findings

From the 81 included studies, 45 were included in meta-analyses. Thirty-six studies were not included due to heterogeneity in tasks or outcomes or missing data. A qualitative synthesis of the unpooled studies along with the reporting quality was performed. For the knee disorders where meta-analysis was possible (i.e., knee OA, TKA, PFP, and ACLR), the qualitative synthesis is presented in Additional file 1E. For those where meta-analysis was not possible (i.e., PT and ACLD), the qualitative synthesis is presented below. The SMD and 95% CI of the unpooled studies are presented in Additional file 1F.

### Knee OA versus Controls

#### Trunk Kinematics in the Sagittal Plane

**Fig. 2:**
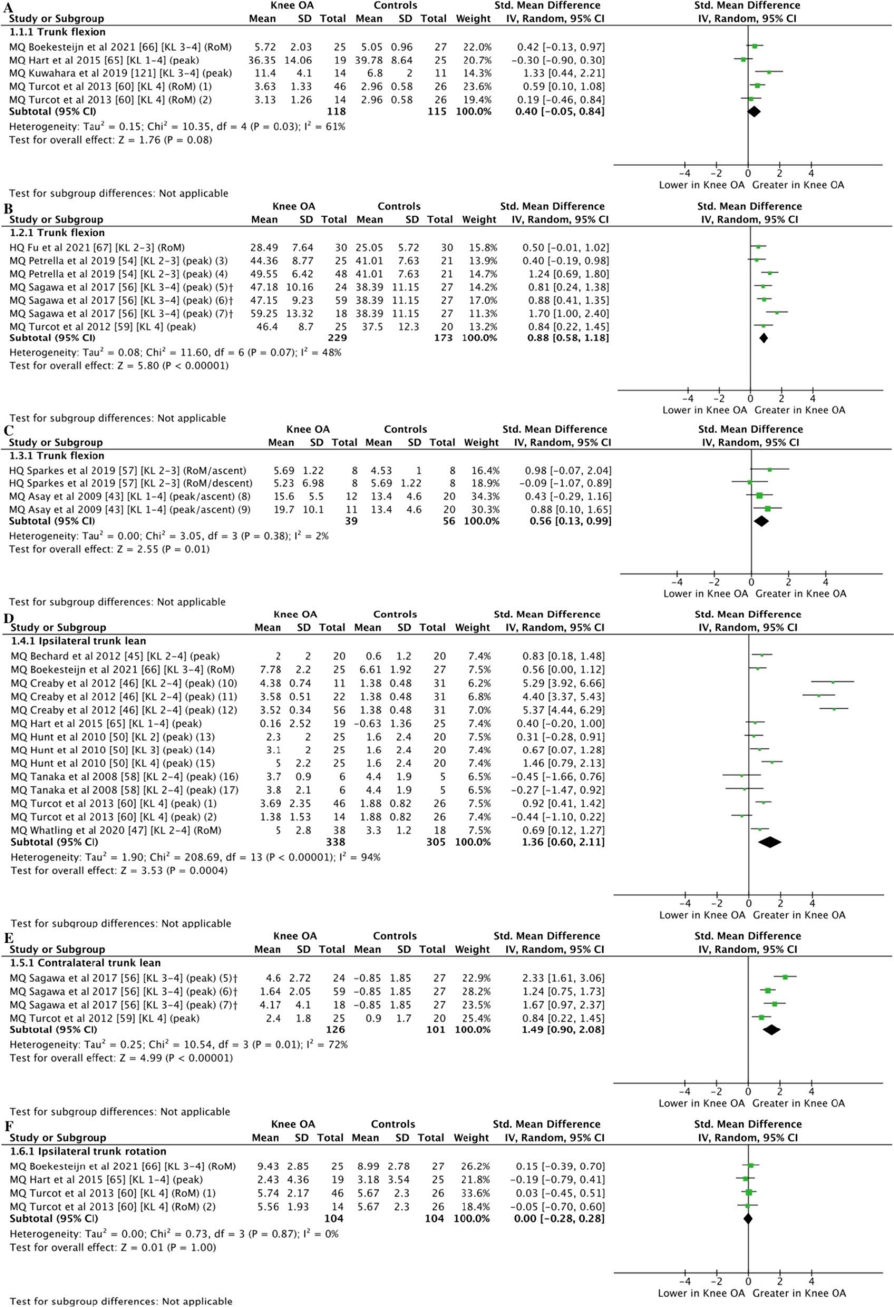
Meta-analyses of trunk kinematics in individuals with knee OA compared to controls. Trunk kinematics in the sagittal plane during walking (**A**), sit-to-stand (**B**), and stepping tasks (**C**). Trunk kinematics in the frontal plane during walking (**D**) and sit-to-stand (**E**). Trunk kinematics in the transverse plane during walking (**F**). (1) varus OA group, (2) valgus OA group, (3) mild OA group, (4) moderate OA group, (5) C-TST OA group, (6) IC-STS OA, (7) SI-STS OA group, (8) less severe OA group, (9) more severe OA group, (10) group with unilateral pain and radiographic knee OA, (11) group with unilateral pain and bilateral radiographic knee OA, (12) group with bilateral pain and radiographic knee OA, (13) mild OA group, (14) moderate OA group, (15) severe OA group, (16) group with unilateral OA pain, (17) group with bilateral OA pain. *HQ* high quality, *MQ* moderate quality, *OA* osteoarthritis, *KL Kellgren–Lawrence* grade. ^†^ Groups divided considering different magnitudes of compensation strategies while performing the task

Moderate-certainty evidence from 4 studies (233 participants) [60, 65, 66, 121] showed no differences between groups for trunk flexion during walking (SMD = 0.40, 95% CI =  − 0.05 to 0.84; *Z* = 1.76, *P* = 0.08 [Fig. 2A]). High-certainty evidence with large effect from 4 studies (402 participants) [54, 56, 59, 67] showed greater trunk flexion in individuals with knee OA compared to controls during sit-to-stand (SMD = 0.088, 95% CI = 0.58–1.18; *Z* = 5.80, *P* = 0.001 [Fig. 2B]). Moderate-certainty evidence with moderate effect from 2 studies (95 participants) [43, 57] showed greater trunk flexion in individuals with knee OA compared to controls during stair ascent and descent (SMD = 0.56, 95% CI = 0.13–.99; *Z* = 2.55, *P* = 0.001 [Fig. 2C]).

#### Trunk Kinematics in the Frontal Plane

Moderate-certainty evidence with large effect from 8 studies (643 participants) [45–47, 50, 58, 60, 65, 66] showed greater ipsilateral trunk lean during walking (SMD = 1.36; 95% CI = 0.60–2.11; *Z* = 3.53, *P* = 0.004 [Fig. 2D]). The high heterogeneity (*I*^2^ = 94%) was mainly influenced by the results of Creaby et al. [46], which was confirmed following visual inspection. A sensitivity analysis was then performed with the removal of Creaby et al. [46]. Results changed to moderate-certainty evidence with moderate effect of greater ipsilateral trunk lean in individuals with knee OA compared to controls, and the heterogeneity was reduced (SMD = 0.51; 95% CI = 0.20–0.82; *Z* = 3.25, *P* = 0.001 [*I*^2^ = 59%]) (Additional file 1G). High-certainty evidence with large effect from 2 studies (227 participants) [56, 59] showed greater contralateral trunk lean in individuals with knee OA compared to controls during sit-to-stand (SMD = 1.49; 95% CI = 0.90–2.08; *Z* = 4.99, *P* = 0.001 [Fig. 2E]).

#### Trunk Kinematics in the Transverse Plane

Moderate-certainty evidence from 3 studies (208 participants) [60, 65, 66] showed no differences between groups for ipsilateral trunk rotation during walking (SMD = 0.00; 95% CI =  − 0.28 to 0.28; *Z* = 0.01, *P* = 1.00 [Fig. 2F]).

### TKA versus Controls

#### Trunk Kinematics in the Sagittal Plane

**Fig. 3:**
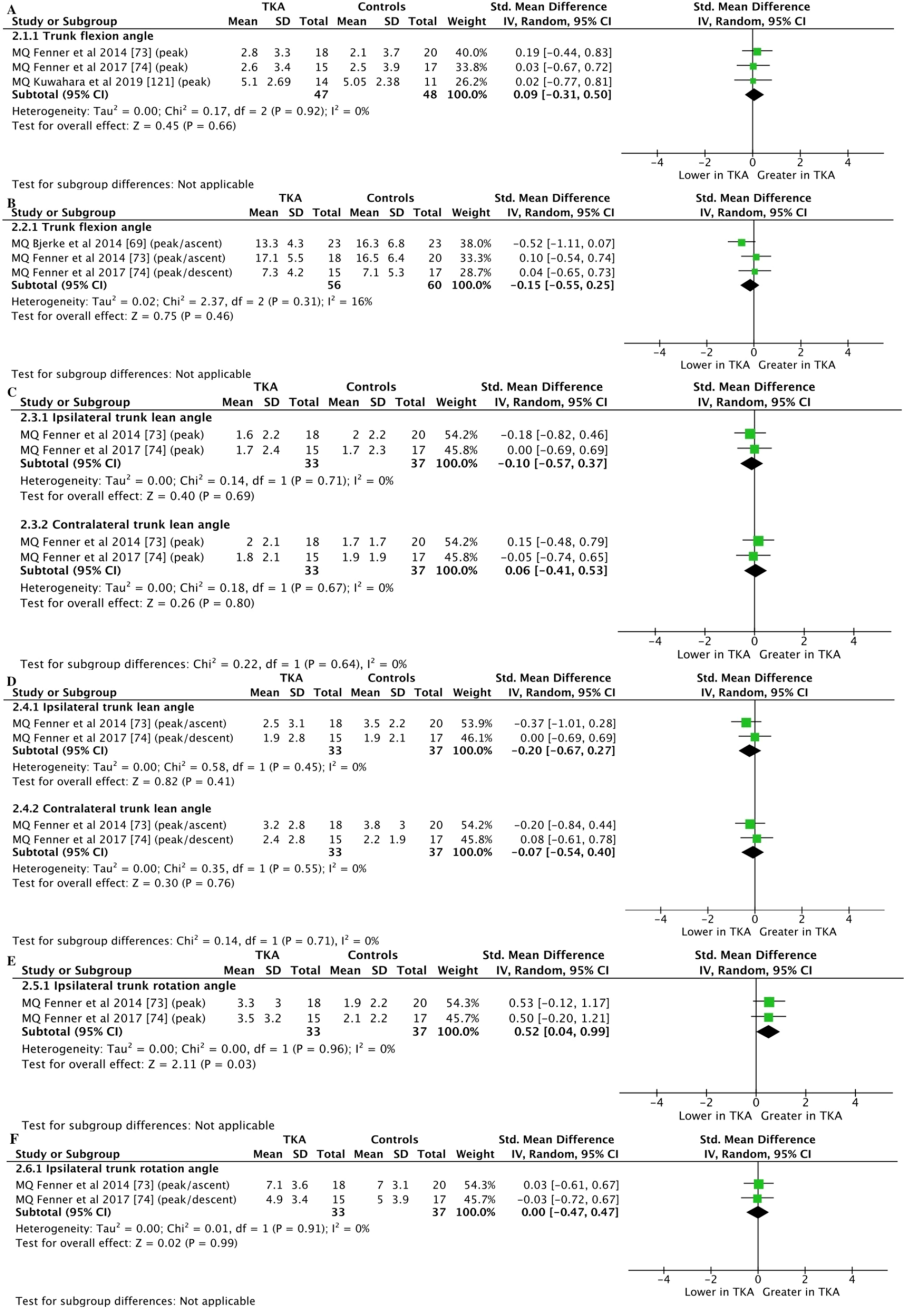
Meta-analyses of trunk kinematics in individuals with TKA compared to controls. Trunk kinematics in the sagittal plane during walking (**A**) and stepping tasks (**B**). Trunk kinematics in the frontal plane during walking (**C**) and stepping tasks (**D**). Trunk kinematics in the transverse plane during walking (**E**) and stepping tasks (**F**). *TKA* Total knee arthroplasty, *MQ* moderate quality

Moderate-certainty evidence from 3 studies (95 participants) [73, 74, 121] showed no differences between groups for trunk flexion during walking (SMD = 0.09; 95% CI =  − 0.31 to 0.50; *Z* = 0.45, *P* = 0.66 [Fig. 3A]), whereas moderate-certainty evidence from 3 studies (116 participants) [69, 73, 74] also showed no differences between groups during stair ascent and descent (SMD =  − 0.15; 95% CI =  − 0.55 to 0.25; *Z* = 0.75, *P* = 0.46 [Fig. 3B]).

#### Trunk Kinematics in the Frontal Plane

Moderate-certainty evidence from 2 studies (70 participants) [73, 74] showed no differences between groups for ipsilateral trunk lean during walking (SMD =  − 0.10; 95% CI =  − 0.57 to 0.37; *Z* = 0.40, *P* = 0.69 [Fig. 3C]) and stair ascent and descent (SMD =  − 0.20; 95% CI =  − 0.67 to 0.27; *Z* = 0.82, *P* = 0.41 [Fig. 3D]). Moderate-certainty evidence from 2 studies (70 participants) [73, 74] showed no differences between groups for contralateral trunk lean during walking (SMD = 0.06; 95% CI =  − 0.41 to 0.53; *Z* = 0.26, *P* = 0.80 [Fig. 3C]) and stair ascent and descent (SMD =  − 0.07; 95% CI =  − 0.54 to 0.40; *Z* = 0.30, *P* = 0.76 [Fig. 3D]).

#### Trunk Kinematics in the Transverse Plane

Moderate-certainty evidence with moderate effect from 2 studies (70 participants) [73, 74] showed greater ipsilateral trunk rotation (SMD = 0.52; 95% CI = 0.04 to 0.99; *Z* = 2.11, *P* = 0.03 [Fig. 3E]) in individuals with TKA compared to controls during walking, whereas moderate-certainty evidence from 2 studies (70 participants) [73, 74] showed no differences between groups for ipsilateral trunk rotation during stair ascent and descent (SMD = 0.00; 95% CI =  − 0.47 to 0.47; *Z* = 0.02, *P* = 0.99 [Fig. 3F]).

### PFP versus Controls

#### Trunk Kinematics in the Sagittal Plane

**Fig. 4:**
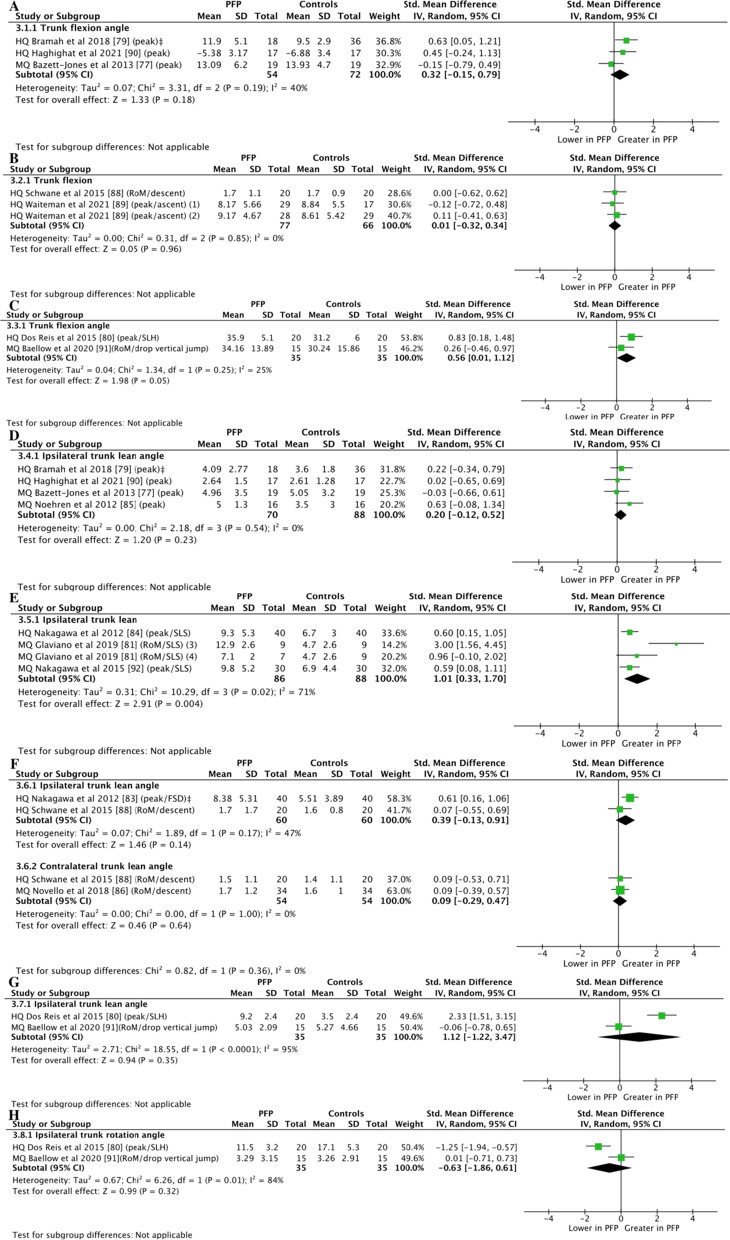
Meta-analyses of trunk kinematics and strength in individuals with PFP compared to controls. Trunk kinematics in the sagittal plane during running (**A**), stepping (**B**), and landing tasks (**C**). Trunk kinematics in the frontal plane during running (**D**), squatting (**E**), stepping (**F**), and landing tasks (**G**). Trunk kinematics in the transverse plane during landing tasks (**H**). (1) PFP and control groups with knee crepitus, PFP and control groups without knee crepitus, (3) PFP group with elevated fear avoidance beliefs, (4) PFP group with low fear avoidance beliefs. *PFP* patellofemoral pain*, HQ* high quality, *MQ* moderate quality, *SLH* single-leg hop for distance, *SLS* single-leg squat, *FSD* forward step-down. ^‡^Data supplied by author

Moderate-certainty evidence from 3 (126 participants) [77, 79, 90] and 2 studies (143 participants) [88, 89] showed no differences between groups for trunk flexion during running (SMD = 0.32; 95% CI =  − 0.15 to 0.79; *Z* = 1.33, *P* = 0.18 [Fig. 4A]) and stair ascent and descent (SMD = 0.01; 95% CI =  − 0.32 to 0.34; *Z* = 0.05, *P* = 0.96 [Fig. 4B]), respectively. Moderate-certainty evidence with moderate effect from 2 studies (70 participants) [80, 91] showed greater trunk flexion during landing tasks (SMD = 0.56; 95% CI = 0.01–1.12; *Z* = 1.98, *P* = 0.05 [Fig. 4C]).

#### Trunk Kinematics in the Frontal Plane

Moderate-certainty evidence from 4 studies (158 participants) [77, 79, 85, 90] showed no differences between groups for ipsilateral trunk lean during running (SMD = 0.20; 95% CI =  − 0.12 to 0.52; *Z* = 1.20, *P* = 0.23 [Fig. 4D]). Moderate-certainty evidence with large effect from 3 studies (174 participants) [81, 84, 92] showed greater ipsilateral trunk lean in individuals with PFP compared to controls during single-leg squat (SMD = 1.01; 95%CI = 0.33–1.70; *Z* = 2.91, *P* = 0.004 [Fig. 4E]). Moderate-certainty evidence from 2 studies (120 participants) [83, 88] showed no differences between groups for ipsilateral trunk lean during stepping tasks (SMD = 0.39; 95% CI =  − 0.13 to 0.91; *Z* = 1.46, *P* = 0.014 [Fig. 4F]). Moderate-certainty evidence from 2 studies (108 participants) [86, 88] showed no differences between groups for contralateral trunk lean during stair descent (SMD = 0.09; 95% CI =  − 0.29 to 0.47; *Z* = 0.46, *P* = 0.64 [Fig. 4F]). Very low-certainty evidence from 2 studies (70 participants) [80, 91] showed no differences between groups for ipsilateral trunk lean during landing tasks (SMD = 1.12; 95% CI =  − 1.22 to 3.47; *Z* = 0.94, *P* = 0.35 [Fig. 4G]).

#### Trunk Kinematics in the Transverse Plane

Very low-certainty evidence from 2 studies (70 participants) [80, 91] showed no differences between group for ipsilateral trunk rotation during landing tasks (SMD =  − 0.63; 95% CI =  − 1.86 to 0.61; *Z* = 0.99, *P* = 0.32 [Fig. 4H]).

### PT versus Controls

#### Trunk Kinematics in the Sagittal Plane

One moderate-quality study [96] reported no differences between groups for trunk flexion during three different variations of squatting tasks. One high-quality study [95] reported no differences between groups for trunk flexion during a drop vertical landing.

### ACLD versus Controls

#### Trunk Kinematics in the Sagittal Plane

One moderate-quality study [100] reported no differences between groups for trunk flexion during walking. One moderate-quality study [100] reported greater trunk flexion in individuals with ACLD during stair ascent and descent. One moderate-quality study [122] reported greater trunk flexion in individuals with ACLD during landing from a single-leg hop for distance, while no differences between groups were reported during jumping and landing from a single-leg vertical hop [122]. Two moderate quality studies [97, 99] reported lower two-dimensional trunk flexion displacement in individuals with ACLD during landing and cutting tasks [97] and single-leg landing [99].

#### Trunk Kinematics in the Frontal Plane

One moderate-quality study [100] reported lower contralateral trunk lean in individuals with ACLD during walking, while another moderate-quality study [120] reported no differences between groups during walking and running. One moderate-quality study [101] reported greater ipsilateral trunk lean in individuals with ACLD during single-leg squat, while another moderate-quality study [120] reported no differences between groups. One moderate-quality study [100] reported greater contralateral trunk lean in individuals with ACLD during stair ascent and descent. One moderate-quality study [120] reported lower contralateral trunk lean in individuals with ACLD during landing of single-leg hop for distance. One moderate-quality study [97] reported greater two-dimensional lateral trunk displacement (side not specified) in individuals with ACLD during landing and cutting tasks.

#### Trunk Kinematics in the Transverse Plane

One moderate-quality study [100] reported greater ipsilateral trunk rotation during walking and lower contralateral trunk rotation during stair ascent and descent.

### ACLR versus Controls

#### Trunk Kinematics in the Sagittal Plane

**Fig. 5:**
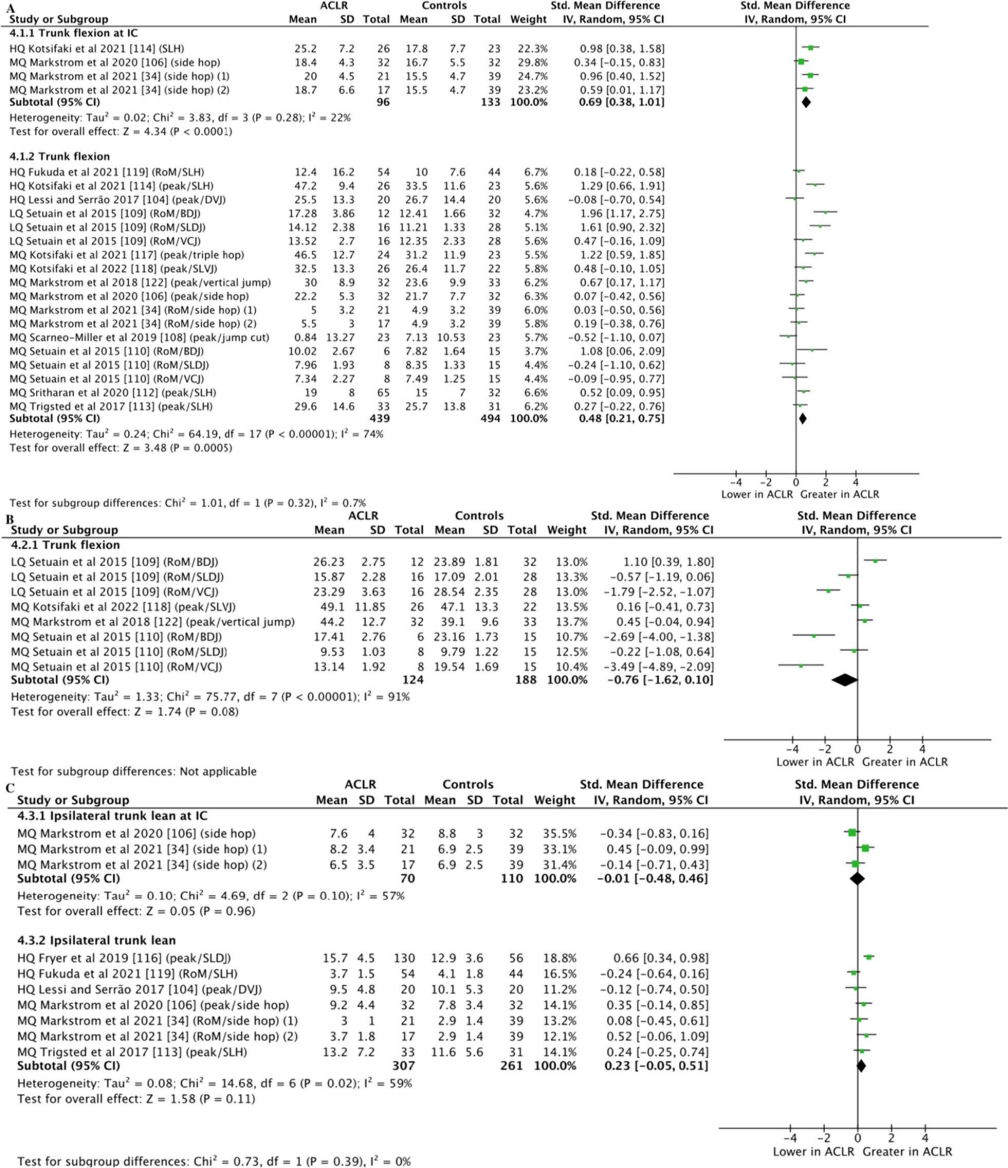
Meta-analyses of trunk kinematics in individuals with ACLR compared to controls. Trunk kinematics in the sagittal plane during landing (**A**) and jumping (**B**) tasks. Trunk kinematics in the frontal plane during landing tasks (**C**). (1) ACLR group with high fear of reinjury, (2) ACLR group with low fear of reinjury. *ACLR* anterior cruciate ligament reconstruction, *HQ* high quality, *LQ* low quality, *MQ* moderate quality, *BDJ* bilateral drop jump, *DVJ* drop vertical jump, *SLDJ* single-leg drop jump, *SLH* single-leg hop for distance, *SLVJ* single-leg vertical jump, *VCJ* single-leg vertical countermovement jump

Moderate-certainty evidence with moderate effect from 3 studies (229 participants) [34, 106, 114] showed greater trunk flexion at IC in individuals with ACLR compared to controls during landing tasks (SMD = 0.69; 95% CI = 0.38–1.01; *Z* = 4.34, *P* = 0.001 [Fig. 5A]). Moderate-certainty evidence with small effect from 13 studies (933 participants) [34, 104, 106, 108–110, 112–114, 117–119, 122] showed greater peak/RoM trunk flexion in individuals with ACLR compared to controls during landing tasks (SMD = 0.48; 95% CI = 0.21–0.75; *Z* = 3.48, *P* = 0.005 [Fig. 5A]). Very low-certainty evidence from 4 studies (312 participants) [109, 110, 118, 122] showed no differences between groups for trunk flexion during jumping tasks (SMD =  − 0.76; 95% CI =  − 1.62 to 0.10; *Z* = 1.74, *P* = 0.08 [Fig. 5B]).

#### Trunk Kinematics in the Frontal Plane

Low-certainty evidence from 2 studies (180 participants) [34, 106] showed no differences between groups for ipsilateral trunk lean at IC during landing tasks (SMD =  − 0.01; 95% CI =  − 0.48 to 0.46; *Z* = .05, *P* = .96 [Fig. 5C]). Moderate-certainty evidence from 6 studies (568 participants) [34, 104, 106, 113, 116, 119] showed no differences between groups for ipsilateral trunk lean (peak/RoM) during landing tasks (SMD = 0.23; 95% CI =  − 0.05 to 0.51; *Z* = 1.58, *P* = 0.11 [Fig. 5C]).

### Evidence Gap Map

Evidence gap maps for discrete variables of trunk kinematics and kinetics as well as waveform analyses of trunk biomechanics are presented in Additional files 1H and 1I, respectively. Overall, PT is the knee disorder with the least amount of studies (2 [95, 96]), which have only investigated trunk kinematics in the sagittal plane. Similarly, trunk kinematics in individuals with ACLD was investigated in only 7 studies [97–101, 120, 122], which were heterogeneous for tasks and outcomes and could not be pooled. Inconclusive evidence is outlined for trunk kinematics in the transverse plane, with studies not clearly specifying which side the trunk is rotating toward. There was also insufficient evidence for trunk kinetics, with investigations limited to ACLR. Furthermore, waveform analyses of trunk kinematics or kinetics have not been performed in individuals with ACLD, PT, or TKA.

## Discussion

This review identified 81 studies investigating trunk biomechanics across 6 different knee disorders (i.e., knee OA, PFP, PT, ACLD, TKA and ACLR). In individuals with knee OA, greater trunk flexion and contralateral trunk lean during sit-to-stand, greater trunk flexion during stepping tasks, and greater ipsilateral trunk lean during walking were identified. On the other hand, no alterations were identified in trunk sagittal or frontal planes in individuals with TKA. Individuals with PFP demonstrated greater trunk flexion during landing tasks and greater ipsilateral trunk lean during single-leg squat. Individuals with ACLR also demonstrated greater trunk flexion during landing tasks. These findings show that changes in trunk biomechanics in the sagittal and frontal planes are present in the most common knee disorders. This systematic review also highlights areas with the need for more research, with noticeable lack of investigations in individuals with PT or ACLD.

### Trunk Biomechanics for Participants with Knee OA

Individuals with knee OA demonstrated greater trunk flexion during sit-to-stand [54, 56, 59] and stair negotiation [43, 57]. Sit-to-stand and stair negotiation require high knee extensor moments and generate great knee loading [59, 123–125]. Since the increase in trunk flexion can reduce knee loading [126], these findings possibly represent a compensatory strategy to reduce knee loading and avoid or control knee pain [53, 68]. However, the persistent use of this strategy may lead to chronic disuse of knee extensors and subsequent muscle weakness [127]. Decreased knee extensor strength is associated with increased risk of worsening of knee pain and lower physical function [128].

Individuals with knee OA presented greater ipsilateral trunk lean during walking [45–47, 50, 58, 60, 65]. This is likely a mechanism to reduce high knee varus moments and medial knee loading, which are associated with the progression of knee OA severity [129] and pain [130]. Although increasing ipsilateral trunk lean reduces knee varus moments in a dose–response manner [17], with greater angulations being associated with worse radiographic knee OA [50], its effect on pain is inconclusive [17] and subsequent hip abductor weakness may be an unintended consequence [10].

Individuals with knee OA demonstrated greater contralateral trunk lean during sit-to-stand (moderate-certainty evidence with large effect) [56, 59]. While this strategy is likely employed to reduce knee loads on the affected knee [56, 59], it may overload the unaffected or less affected knee, leading also to unintended consequences [50, 131].

### Trunk Biomechanics for Participants with TKA

No alterations were identified in trunk sagittal and frontal planes during walking and stepping tasks in individuals with TKA [73, 74, 121]. It has been suggested that the absence of differences between individuals with TKA and controls may be due to positive effects of surgical and conservative managements on knee pain, limiting the need for trunk compensations [73, 74, 121]. In fact, a recent systematic review [132] has identified that excessive trunk amplitudes during walking, as seen pre-operatively, decreased after TKA. However, care should be taken when interpreting our findings due to the limited number of studies included in this review. In addition, more studies are warranted investigating sit-to-stand as it is the task that most consistently elicits trunk compensations [132] and it was not investigated in individuals with TKA.

### Trunk Biomechanics for Participants with PFP

Individuals with PFP presented greater trunk flexion during landing tasks [80, 91], whereas no differences were found during running and stepping tasks [77, 79, 88–90]. Landing tasks impose higher patellofemoral joint loading compared to walking, stepping, and squatting tasks [133] and may reflect the upper limits of lower‐limb joint loading experienced [134]. Since PFP commonly affects young physically active individuals [2], it may be that only a higher knee loading task such as landing would elicit a compensatory trunk flexion. In tasks with lower knee loading, other compensatory strategies may be used such as the modification of step length in running [135] and cadence in stair negotiation [136].

Individuals with PFP presented greater ipsilateral trunk lean during single-leg squat [81, 84, 92]. Greater ipsilateral trunk lean during single-leg squat has been reported as a strategy to compensate for hip abductor weakness in individuals with PFP [10]. Although ipsilateral trunk lean can reduce the demand on the hip abductors [10], such an adaptation might increase knee valgus moments [10, 97, 137], and patellofemoral joint loading [137]. Future studies are required to understand long-term consequences of compensatory ipsilateral trunk lean in individuals with PFP.

### Trunk Biomechanics for Participants with ACLD

The qualitative synthesis suggests that individuals with ACLD may alter trunk motion during stepping and landing tasks, although there is no consistency over the direction of these changes. Two studies reported a more extended trunk posture in individuals with ACLD compared to controls during landing tasks [97, 99], while 2 other studies reported greater trunk flexion during landing and stepping tasks [98, 100]. A more extended trunk posture represents a stiffened movement that may be indicative of fear of reinjury [31]. Conversely, increased trunk flexion is indicative of quadriceps weakness and/or avoidance [138]. These findings indicate that there seems to be more than just one compensatory trunk strategy and individuals with ACLD may respond differently given their fear of reinjury or reliance on quadriceps function. However, further studies are warranted due to the low number of studies included in this review.

There is also conflicting evidence for trunk kinematics in the frontal plane. Greater ipsilateral trunk lean was reported in individuals with ACLD during single-leg landing and squat [97, 101], while greater contralateral trunk lean was reported during stair negotiation [100], suggesting that changes in trunk kinematics in the frontal plane may be task dependent. Alternate stepping may allow greater trunk displacement in the frontal plane and a contralateral trunk lean [139, 140], while only an ipsilateral trunk lean may be possible during single-leg landing and squat. Further research is needed as we were unable to pool data due to the low number of studies.

### Trunk Biomechanics for Participants with ACLR

Moderate-certainty evidence demonstrated greater trunk flexion in individuals with ACLR during landing tasks [104, 106, 108–110, 112–114, 122], likely to reduce quadriceps demand and knee pain. A subgroup of athletes at the time of return-to-sport after ACLR with low-quadriceps symmetry between limbs (i.e., < 85% of isometric strength) presented greater trunk flexion during landing compared to controls [141]. In the same study, quadriceps strength symmetry was found to be a unique and significant predictor of landing symmetry in peak trunk flexion [141]. A recent study [138] has also reported that reductions in peak trunk flexion are correlated to improved pain scores and increased knee extensor moments between baseline and follow-up (2 and 8 years post-ACLR). Therefore, the identification of such a strategy may indicate the need to enhance quadriceps strength and function, especially at the time of return-to-sport [141]. Greater quadriceps neuromuscular function would provide better knee energy attenuation and reduce the level of pain [138] and risk of secondary injury [142, 143].

### Clinical Implications

The findings of this systematic review support the assessment of trunk biomechanics in individuals with OA, PFP, or ACLR to identify possible targets for rehabilitation, especially for those with chronic pain. Reliable, valid, and time- and cost-effective field-based assessments of biomechanics can be performed through two-dimensional motion capture [144–149] and wearable inertial measurement units (IMU) [150, 151]. To target altered trunk kinematics in the sagittal and frontal planes, clinicians can consider interventions like progressive resistance training [152], virtual reality training [153], and motor feedback control using IMUs, visual or verbal feedback [154–156]. The assessment of trunk biomechanics may also provide an objective marker of quadriceps avoidance strategies [138], hip muscle weakness [10, 138], and disease prognosis [50, 138].

The compensatory strategies identified in this systematic review may be useful as a temporary strategy when pain is exacerbated (acute phases) to alleviate symptoms. They could be part of self-management programs [157, 158], although their effectiveness needs further testing. Also, long-term use of these strategies may lead to chronic disuse of the knee extensors or hip abductors.

### Limitations and Future Directions

Although the findings of this systematic review highlight that individuals with knee OA, PFP, and ACLR seem to present with altered trunk biomechanics, the cross-sectional nature of the included studies precludes us from establishing cause and effect, as well as long-term consequences. Prospective cohort studies or randomized controlled trials are needed to determine the causal relationship and long-term consequences of altered trunk biomechanics in individuals with knee OA, PFP, and ACLR.

Some meta-analyses of this systematic review included a small number of studies, which may limit a more precise estimation of the effect. Some meta-analyses that included few studies had low values of heterogeneity. Caution is needed when interpreting these values as they may considerably change upon the inclusion of new studies. Further studies may improve the precision of the estimated effect and provide a more accurate interpretation of heterogeneity.

The findings of this systematic review outline the lack of studies in individuals with PT and ACLD, as well as insufficient evidence for trunk kinematics in the transverse plane and trunk kinetics across knee disorders. Further research is warranted to fill these gaps and strengthen future recommendations.

## Conclusion

Individuals with knee OA, PFP, or ACLR present with altered trunk kinematics in the sagittal and frontal planes. No trunk biomechanical alterations were identified in individuals with TKA, whereas a noticeable lack of investigation in individuals with PT or ACLD precludes any conclusion. Our findings support the assessment of trunk biomechanics in individuals with OA, PFP, or ACLR to identify possible targets for rehabilitation and avoidance strategies.

## Supplementary Information

**Additional file 1.** Supplementary material including: search strategy (A), reasons for exclusion of studies excluded after full-text screening (B), summary of included studies (C), methodological quality assessment of included studies (D), qualitative and quantitative syntheses of unpooled data (E and F), sensitivity analysis (G), and evidence gap maps (H and I).

## Data Availability

All data generated or analyzed during this study are included in this published article and in Additional file 1: Appendices A, B, C, D, E, F, G, H, and I.
