# Supplementary material for: Trunk Biomechanics in Individuals with Knee Disorders: A Systematic Review with Evidence Gap Map and Meta-analysis
Source: Sports Med Open. 2022 Dec 12;8:145. doi: 10.1186/s40798-022-00536-6 (PMC9742076; doi:10.1186/s40798-022-00536-6)
Supplement: Supplementary file 1 — Additional file 1. Supplementary material including: search strategy (A), reasons for exclusion of studies excluded after full-text screening (B), summary of included studies (C), methodological quality assessment of included studies (D), qualitative and quantitative syntheses of unpooled data (E and F), sensitivity analysis (G), and evidence gap maps (H and I). [file 40798_2022_536_MOESM1_ESM.docx]

**Trunk biomechanics in individuals with knee disorders: A systematic review with evidence gap map and meta-analysis**

**Running head:** Trunk biomechanics in knee disorders

**Journal:** Sports Medicine Open

**Additional file 1A** Search strategy

1. Knee injury/

2. Knee pain.af.

3. Knee disorder*.af.

4. 1 or 2 or 3

5. Patellofemoral Pain Syndrome.af.

6. Patellofemoral Pain.af.

7. anterior knee pain.af.

8. ((patell* or femoropatell* or femoro patell* or retropatell* or peripatell*) and (pain or syndrome or dysfunction)).af.

9. ((chondromalac* or chondropath* or chondrosis) and (knee* or patell* or femoropatell* or femoro patell* or retropatell*)).af.

10. Chondromalacia Patellae.af.

11 5 or 6 or 7 or 8 or 9 or 10

12. ((anterior and cruciate* and ligament*) or acl).af.

13. (Anterior Cruciate Ligament and (Rupture or Tear or Lesion or Deficiency or Injury or reconstruct*))

14. 12 or 13

15. knee osteoarthritis/

16. knee osteoarthr*.af.

17. (degenerative and (knee or arthr*)).af.

18. knee arthr*.af.

19. ((patell* or tibiofemor*) and (osteoarthr* or arthr*)).af.

20. 15 or 16 or 17 or 18 or 19

21. (knee and (tendinitis or tendinopathy)).af.

22. (patellar and (tendinitis or tendinopathy)).af.

23. (menisc* and (tear* or lesion)).af.

24. osgood-schlatter/

25. 4 or 11 or 14 or 20 or 21 or 22 or 23 or 24

26. trunk.af.

27. core.af.

28. 26 or 27

29. biomechanic*.af.

30. kinetic*.af.

31. kinematic*.af.

32. moment*.af.

33. motion analy*.af.

34. 29 or 30 or 31 or 32 or 33

35. 28 and 34

36. 25 and 35

**Additional file 1B** Studies excluded after full-text screening, with reasons for exclusion

| **Study** | **Reason for exclusion** |
| --- | --- |
| Almeida et al. 2016 | Wrong outcome: only trunk muscle function data (strength) |
| Anan et al. 2010 | Language |
| Armaki et al. 2020 | Wrong outcome: analysis of active and passive trunk repositioning error at 30 degrees of trunk flexion |
| Biabanimoghadam et al. 2013 | Abstract |
| Biabanimoghadam et al. 2016 | Wrong outcome: only trunk muscle function data (electromyography) |
| Bokaee et al. 2010 | Language |
| Bouchouras et al. 2015 | Wrong outcome: only trunk muscle function data (electromyography) |
| Briani et al. 2019 | Wrong outcome: only trunk muscle function data (ultrasound) |
| Bronner et al. 2002 | Wrong outcome: analysis of pelvis |
| Burnett et al. 2010 | Abstract |
| Carvalho et al. 2020 | Abstract |
| Chiba et al. 2017 | Abstract |
| Colangelo et al. 2019 | Abstract |
| Colné 2010 | Abstract |
| Corkery et al. 2016 | Abstract |
| Cowan et al. 2009 | Wrong outcome: only trunk muscle function analysis (strength) |
| Curry et al. 2013 | Abstract |
| Della Villa et al. 2021 | Wrong comparator: no comparisons between groups were performed for trunk biomechanics |
| De Oliveira Mianutti et al. 2021 | Abstract |
| Dingenen et al. 2019 | Wrong comparator: included runners with iliotibial band syndrome |
| Doozan et al. 2021 | Wrong outcome: reliability data only (no comparisons between groups) |
| Dorosti et al. 2017 | Language |
| Dos Reis et al. 2014 | Wrong study design: systematic review |
| Ernst et al. 2000 | Wrong outcome: only correlation analysis was performed for trunk variables (no comparison between groups) |
| Falstrom et al. 2017 | Wrong outcome: Clinical tests data |
| Ferreira et al. 2019 | Wrong outcome: analysis of movement deviation profile with no specific analyses of trunk biomechanics |
| Flowers et al 2021 | Wrong outcome: only trunk muscle function data (electromyography) |
| Foucher et al. 2020 | Laboratory-induced task: Walking under postural perturbations |
| Frank et al. 2011 | Abstract |
| Hálfdanardóttir et al. 2015 | Abstract |
| Haghighat et al. 2021 | Wrong outcome: analysis of intersegmental couplings angle variability of trunk with other joints |
| Hébert-Losier et al. 2018 | Wrong patient population: over 80% of the sample with anterior cruciate ligament deficiency had concomitant knee osteoarthritis |
| He and Wimmer 2021 | Wrong study design: systematic review |
| Hewett and Myer 2011 | Wrong study design: literature review |
| Hlavacková and Janura 2007 | Wrong outcome: analysis of postural control and trunk displacement during arm and forearm positioning |
| Huang et al. 2014 | Participants with a non-specific diagnosis |
| Huang et al. 2021 | Wrong outcome: no trunk biomechanics data |
| Kalytczak et al. 2016 | Wrong outcome: no trunk biomechanics data |
| Lai et al. 2012 | Abstract |
| Levinger et al. 2016 | Laboratory-induced task: Balance recovery task |
| Liikavainio et al. 2010 | Wrong outcome: no trunk biomechanics data |
| Lin et al. 2015 | Participants with a non-specific diagnosis |
| Markstrom et al. 2021 | Wrong study design: reliability study |
| Maryama et al. 2018 | Wrong outcome: Clinical tests data |
| Mazloum and Sobhani 2017 | Language |
| McClelland et al. 2015 | Abstract |
| Motealleh et al. 2014 | Wrong outcome: only trunk muscle function analysis (electromyography) |
| Motealleh et al. 2019 | Wrong outcome: analysis of postural control with center of pressure displacement |
| Noehren et al. 2011 | Abstract |
| O’Malley et al. 2018 | Wrong outcome: no trunk biomechanics data |
| Papadonikolakis et al. 2003 | Wrong study design: literature review |
| Pater et al. 2016 | Laboratory-induced task: Laboratory-induced trip and the treadmill-delivered perturbation during walking |
| Pedroso et al. 2019 | Abstract |
| Perraton et al. 2014 | Abstract |
| Petrella et al. 2019 | Abstract |
| Phillips 2006 | Thesis |
| Poston et al. 2021 | Wrong comparator: comparisons between groups performed pre-injury |
| Preece et al. 2019 | Abstract |
| Preece and Alghamdi 2020 | Abstract |
| Reed-Jones & Vallis 2007 | Laboratory-induced task: Descent of a 20° incline ramp followed by a cutting maneuver during different ambient lighting conditions |
| Rodrigues et al. 2021 | Wrong outcome: only trunk muscle function analysis (ultrasonography) |
| Sagawa et al. 2017 | Wrong comparator: no comparisons between groups were performed |
| Severin et al. 2017 | Participants with a non-specific diagnosis |
| Shi et al. 2015 | Wrong outcome: analyses were performed with the non-injured limb as reference |
| Shirazi et al. 2014 | Laboratory-induced task: Sudden external perturbation applied laterally to the pelvis |
| Silva et al. 2017 | Abstract |
| Stalter et al. 2006 | Abstract |
| Staples et al. 2020 | Wrong outcome: postural stability |
| Stensdotter et al. 2009 | Laboratory-induced task: Unpredictable perturbation task (a reaction time task performed with bimanual forward reach and grasp) |
| Tecco et al. 2005 | Wrong outcome: lower trapezius activation at mandibular rest position, and during maximal voluntary clenching. |
| Tecco et al. 2006 | Duplicate data set: no descriptive data |
| Tecco et al. 2007 | Wrong outcome: only trunk muscle function analysis (electromyography) |
| Tengman et al. 2015 | Wrong outcome: medio-lateral position of the center of mass in relation to knee and ankle joint centers) data |
| Ukishiro et al. 2017 | Abstract |
| Van Criekinge et al. 2021 | Abstract |
| Viggiano et al. 2014 | Wrong outcome: no trunk biomechanics data |
| Wada et al. 2021 | Wrong outcome: no trunk biomechanics data |
| Werner and Barrios 2021 | Wrong outcome: Clinical tests data |
| Willson 2007 | Thesis |
| Willson 2008 | Wrong outcome: only trunk muscle function analysis (strength) |
| Willson and Davies 2009 | Duplicate data set: later manuscript |
| Yamazaki et al. 2010 | Wrong outcome: no trunk biomechanics data |
| Zazulak et al. 2007 | Laboratory-induced task: Sudden force release in flexion, extension, and lateral directions of isometric trunk exertions |

**References**

1. Almeida GPL, De Moura Campos Carvalho e Silva AP, França FJR, Magalhães MO, Burke TN, Marques AP. Relationship between frontal plane projection angle of the knee and hip and trunk strength in women with and without patellofemoral pain. Journal Back Musculoskeletal Rehabilitation. 2016;29:259–66.
2. Anan M, Tokuda K, Kito N, Shinkoda K. Kinematic analysis of sit-to-stand motion in knee osteoarthritis. Rigakuryoho Kagaku. 2010;25:755-60.
3. Armaki RH, Abbasnia K, Motealleh A. Comparison of trunk flexion proprioception between healthy athletes and athletes with patellofemoral pain. Journal of Sport Rehabilitation. 2020;30:430-36.
4. Biabanimoghadam M, Shizari, RZ, Motealleh, A. Core and lower extremity muscle recruitment pattern in response to an unexpected external perturbation in patients with patellofemoral pain syndrome and healthy individuals. Gait and Posture. 2013;38:S51.
5. Biabanimoghadam M, Motealleh A, Cowan SM.The knee. Core muscle recruitment pattern during voluntary heel raises is different between patients with patellofemoral pain and healthy individuals. The knee. 2016;23:382-86.
6. Bokaee F, Nasseri N, Mazaheri H, Fakhari Z, Jalaee S. Strengths of lower extremity and lower trunk muscles in females with patellofemoral pain syndrome. Koomesh. 2010;12(1):22-30.
7. Bouchouras G, Patsika G, Hatzitaki V, Kellis E. Kinematics and knee muscle activation during sit-to-stand movement in women with knee osteoarthritis. Clinical Biomechanics. 2014;30:599-607.
8. Briani RV, Waiteman MC, Albuquerque CE, Gasoto E, Segatti G, Oliveira CB, De Azevedo FM, Silva DO. Lower trunk muscle thickness is associated with pain in women with patellofemoral pain. Journal Ultrasound in Medicine. 2019;38:2685–93.
9. Bronner S, Kaminski TR, Gordon AM. Effect of anterior cruciate ligament reconstruction on the passe movement in elite dancers. Journal of Dance Medicine & Science. 2002;6:110-18.
10. Burnett DR, Campbell-Kyureghyan NH, Topp RV, Quesada PM. Comparison of bilateral kinematics and kinetics during stand-to-sit between healthy subjects and unilateral knee osteoarthritis patients. Conference Proceedings of the Annual Meeting of the American Society of Biomechanics. 2010:3-4.
11. Carvalho C, Pisani GK, Martinez AF, Mancini L, Serrao FV, Serrao, PRMS. Hip abductors strength and trunk, pelvis, hip and knee frontal plane kinematics analysis during single-leg squat in individuals with and without patellofemoral osteoarthritis. Annals of the Rheumatic Diseases. 2020;79:521.
12. Chiba T,Yamanaka M, Sabashi K, Samukawa M, Saitoh H, Uri M, Kobayashi T, Tohyama H. Comparison of the trunk and pelvic kinematics during single-leg standing between individuals with knee osteoarthritis and healthy subjects. Osteoarthritis and Cartilage. 2017;25:S398.
13. Colangelo A, Pavan D, Cibin F, Spolaor F, Guiotto A, Cesana M, Furlan E, Casagrande T, Sawacha Z. The role of muscle forces in elite athletes before and after ACL surgery while performing a drop landing task. Gait and Posture. 2019;73:116-17.
14. Colné P. The course of activity of the gluteus medius and frontal stability of the closed-chain trunk: Elements to consider in the treatment of patellofemoral syndromes. Kinesitherapie. 2010;10:12-13.
15. Corkery M, Cohen E, Esposito M, Newton R, Rogazzo M, Rudnick C, Salanitro B, Santilli R, Yen S-C. Trunk kinematics and motor control in athletes with and without patellofemoral pain during a lateral step-down test. Manual Therapy. 2016;25:e132.
16. Cowan SM, Crossley KM, Bennell KL. Altered hip and trunk muscle function in individuals with patellofemoral pain. British Journal of Sports Medicine. 2009;43:584-88.
17. Curry M, Abraham A, Ireland ML, Lattermann C, Noehren B. Hip strength and trunk control in females scheduled to undergo an anterior cruciate ligament reconstruction. Medicine and Science in Sports and Exercise. 2013;45:222.
18. Della Villa F, Tosarelli F, Ferrari R, Grassi A, Ciampone L, Nanni G, Zaffagnini S, Buckthorpe M. Systematic video analysis of anterior cruciate ligament injuries in professional male rugby players: Pattern, injury mechanism, and biomechanics in 57 consecutive cases. Orthopaedic Journal of Sports Medicine. 2021;9:1-11.
19. De Oliveira Mianutti G, Bomtempo KK, Fachin BP, Pesenti FB, Macedo CSG. Physical Therapy in Sport. 2021;47:e1-e2.
20. Dingenen B, Malliaras P, Janssen T, Ceyssens L, Vanelderen R, Barton C. Two-dimensional video analysis can discriminate differences in running kinematics between recreational runners with and without running-related knee injury. Physical Therapy in Sport. 2019;38:184-91.
21. Doozan M, Bazett-Jones DM, Glaviano NR. Novice versus expert intertester reliability of two-dimensional squatting kinematics in females with and without patellofemoral pain. International Journal of Athletic Therapy & Training. 2021;26:154-60.
22. Dorosti R, Ghasemi M, Khademi-Kalantari K, Akbarzadeh-Baghban A. Comparison of the electrical activity of trunk core muscles and knee muscles in subjects with and without patellofemoral pain during gait. Tehran University medical journal. 2017;75:504-12.
23. Dos Reis AC, Bley AS, Rabelo NDA, Alonso AC, Fukuda TY, Lucareli PRG. Kinematic jump characteristics in women with patellofemoral pain syndrome. Fisioterapia Brasil. 2014:15:227-30.
24. Ernst GP, Saliba E, Diduch DR, Hurwitz SR, Ball DW. Lower extremity compensations following anterior cruciate ligament reconstruction. Physical Therapy. 2000;18:251-60.
25. Falstrom A, Hagglund M, Kvist J. Functional performance among active female soccer players after unilateral primary anterior cruciate ligament reconstruction compared with knee-healthy controls. The American Journal of Sports Medicine. 2016;45:377-85.
26. Ferreira CL, Barton G, Borges LD, Rabelo NDA, Politti F, Lucareli PRG. Step down tests are the tasks that most differentiate the kinematics of women with patellofemoral pain compared to asymptomatic controls. Gait and Posture. 2019;72:129-34.
27. Flowers DW, Brewer W, Ellison J, Mitchell K, Frilot C. Transversus abdominis activation does not alter gait impairments in patients with and without knee osteoarthritis. Clinical Biomechanics. 2021;82:105270.
28. Foucher KC, Pater ML, Grabiner MD. Task‐specific perturbation training improves the recovery stepping responses by women with knee osteoarthritis following laboratory‐induced trips. Journal of Orthopaedic Research. 2020;38:663-69.
29. Frank BS, Goerger BM, Bell DR, Norcross MF, Padua DA, Blackburn T. Trunk neuromuscular control is associated with ACL loading mechanisms during an athletic cutting task. Medicine and Science in Sports and Exercise. 2011;43:S806.
30. Hálfdanardóttir F, Ingvarsson P, Briem K. Proximal effects of unloader bracing for medial knee osteoarthritis. Osteoarthritis and Cartilage. 2015;23:A366-A367.
31. Haghighat F, Rezaie M, Ebrahimi S, Shokouhyan SM, Motealleh A, Parnianpour M. Coordination variability during walking and running in individuals with and without patellofemoral pain part 2: Proximal segments coordination variability. Journal of Medical and Biological Engineering. 2021;41.
32. Hébert-Losier K, Schelin L, Tengman E, Strong A, Hager CK. Curve analyses reveal altered knee, hip, and trunk kinematics during drop-jumps long after anterior cruciate ligament rupture. The Knee. 2018;25:226-39.
33. He J, Wimmer MA. Motion analysis for studying gait modification as a biomechanical intervention for medial knee osteoarthritis. Motion Analysis in Orthopedics. 2021;75:474-83.
34. Hewett TE, Myer GD. The mechanistic connection between the trunk, hip, knee, and anterior cruciate ligament injury. Exercise and Sport Sciences Reviews. 2011;39:161-66.
35. Hlavacková P, Janura M. Kinematic analysis of postural changes in bipedal stance at application of stimulus from external environment and modification of visual scene in patients with anterior cruciate ligament reconstruction. Acta Universitatis Palackianae Olomucensis, Gymnica. 2007;37:23-29.
36. Huang M-T, Lee H-H, Lin C-F, Tsai Y-J, Liao J-C. How does knee pain affect trunk and knee motion during badminton forehand lunges? Journal of Sports Sciences. 2014;32:690-700.
37. Huang Y-L, Mulligan CMS, Johnson ST, Pollard C, Hannigan K, Stutzenberger L, Norcross MF. Explosive quadriceps strength symmetry and landing mechanics limb symmetry after anterior cruciate ligament reconstruction in females. Journal of Athletic Training. 2021;56:912-21.
38. Kalytczak MM, Lucareli PRG, Dos Reis AC, Bley AS, Biasotto-Gonzalez DA, Correa JCF, Politti F. Kinematic and electromyographic analysis in patients with patellofemoral pain syndrome during single leg triple hop test. Gait and Posture. 2016;49:246-51.
39. Lai CP, Li GS, Hung CY, Li JP, Lai CH, Chou SW. Low trunk strengthening effects on patellofemoral pain syndrome. Medicine and Science in Sports and Exercise. 2012;44:280.
40. Levinger P, Nagano H, Downie C, Hayes A, Sanders KM, Cicuttini F, Begg R. Biomechanical balance response during induced falls under dual task conditions in people with knee osteoarthritis. Gait and Posture. 2016;48:106-12.
41. Liikavainio T, Bragge T, Hakkarainen M, Karjalainen PA, Arokoski JP. Gait and muscle activation changes in men with knee osteoarthritis. The Knee. 2010;17:69-76.
42. Lin C-F, Hua S-H, Huang M-T, Lee H-H, Liao J-C. Biomechanical analysis of knee and trunk in badminton players with and without knee pain during backhand diagonal lunges. Journal of Sports Sciences. 2015;33:1429-39.
43. Markstrom JL, Schelin L, Hager CK. A novel standardized side hop test reliably evaluates landing mechanics for anterior cruciate ligament reconstructed persons and controls. Sports Biomechanics. 2021;20:213-29.
44. Maryama ADD, Rasyiqah ARN. Proximal stability assessment of knee osteoarthritis patients. Medicine and Health-Kuala Lumpur. 2018;13:145-57.
45. Mazloum V, Sobhani V. The comparison of core muscles strength and dynamic balance in patients with patellofemoral pain syndrome and healthy individuals. Journal of Zanjan University of Medical Sciences and Health Services. 2017;25:107-19.
46. McCelland J, Webster K, Whitehead T, Feller J. Altered trunk movements during landing in people with anterior cruciate ligament reconstruction. Journal of Science and Medicine in Sport. 2015;19:e60.
47. Motealleh A, Maroufi N, Sarrafzadeh J, Sanjari MA, Saleh N. Comparative evaluation of core and knee extensor mechanism muscle activation patterns in a stair stepping task in healthy controls and Patellofemoral pain patients. Journal of Rehabilitation Sciences and Research. 2014;1:84-91.
48. Motealleh A, Yoosefinejad AK, Ghoddosi M, Azhdari N, Pirouzi S. Trunk postural control during unstable sitting differs between patients with patellofemoral pain syndrome and healthy people. The Knee. 2019;26:26-32.
49. Noehen B, Norber J, Sanchez Z, Cunningham T, Pohl M. The role of hip and trunk mechanics in female runners with patellofemoral pain. Medicine and Science in Sports and Exercise. 2011;43:S805.
50. O’Malley E, Richter C, King E, Strike S, Moran K, Franklyn-Miller A, Moran R. Countermovement jump and isokinetic dynamometry as measures of rehabilitation status after anterior cruciate ligament reconstruction. Journal of Athletic Training. 2018;53:687-95.
51. Papadonikolakis A, Lance C, Stergiou N, Georgoulis AD, Soucacos PN. Compensatory mechanisms in anterior cruciate ligament deficiency. Knee Surgery, Sports Traumatology, Arthroscopy. 2003;11:235-43.
52. Pater ML, Rosenblatt NJ, Grabiner MD. Knee osteoarthritis negatively affects the recovery step following large forward-directed postural perturbations. Journal of Biomechanics. 2016;49:1128-33.
53. Pedroso M, de Almeida AC, Aily J, Gonçalves G, Felinto JD, Ferrari RJ, de Noronha M, Mattiello S. Women with knee osteoarthritis have more IMAT and poor muscle quality in trunk muscles than healthy women. Osteoarthritis and Cartilage. 2019;27:S259-S260.
54. Perraton L, Clark R, Pua Y, Crossley K, Bryant A. Modifiable lower limb biomechanics associated with worse knee function after anterior cruciate ligament reconstruction. Journal of Science and Medicine in Sport. 2014;18:e91.
55. Petrella M, Serrao PRMS, Selistre LA, Gonçalves GH, Mattiello SM. Knee coactivaton and trunk flexion strategies during sit to stand movement performed by individuals with mild and moderate knee osteoarthritis. Osteoarthritis and Cartilage. 2019;27:S130.
56. Phillips HJ. Kinematic and kinetic differences in the trunk, pelvis, and lower extremities in women with and without patellofemoral pain when descending stairs. Seton Hall University [PhD Thesis]. 2006.
57. Poston GR, Schmitt LC, Ithurburn MP, Hugentobler JA, Thomas S, Paterno MV. Reduced 2-D frontal plane motion during single-limb landing is associated with risk of future anterior cruciate ligament graft rupture after anterior cruciate ligament reconstruction and return to sport: A pilot study. J Orthop Sports Phys Ther. 2021;51:82-7.
58. Preece SJ, Alghamdi W, Jones R. Could increased trunk flexion underlie alterations in knee muscle activity in people with knee OA? Osteoarthritis and Cartilage. 2019;27:S116-S117.
59. Preece SJ, Alghamdi W. Inter-individual variation in hip flexor length may explain differences in trunk flexion during walking in people with knee osteoarthritis. Osteoarthritis and Cartilage. 2020;28:S244-S245.
60. Reed-Jones RJ, Vallis LA. Proprioceptive deficits of the lower limb following anterior cruciate ligament deficiency affect whole body steering control. Experimental Brain Research. 2007;182:249-60.
61. Rodrigues R, da Rocha ES, Klein KD, Sonda FC, Pompeo KD, Frasson VB, Vaz MA. Proximal and distal muscle thickness is different in women with patellofemoral pain but is not associated with knee frontal plane projection angle. Journal of Bodywork & Movement Therapies. 2021;25:205-11.
62. Sagawa Y, Bonnefoy-Mazure A, Armand S, Hoffmeyer P, Suva D, Turcot K. Individuals with knee osteoarthritis exhibit altered movement patterns during the sit-to-stand task. Movement & Sport Sciences. 2017;98:39-49.
63. Severin AC, Burkett BJ, McKean MR, Wiegand AN, Sayer MGL. Limb symmetry during double-leg squats and single-leg squats on land and in water in adults with long-standing unilateral anterior knee pain. BMC Sports Science, Medicine & Rehabilitation. 2017;9:1-9.
64. Shi D, Li N, Wang Y, Jiang S, Li S, Zhu W. Gait modification strategies of trunk over left stance phase in patients with right anterior cruciate ligament deficiency. International Journal of Clinical and Experimental Medicine. 2015;8:13424-34.
65. Shirazi ZR, Moghaddam MB, Motealleh A. Comparative evaluation of core muscle recruitment pattern in response to sudden external perturbations in patients with patellofemoral pain syndrome and healthy subjects. Archives of Physical Medicine and rehabilitation. 2014;95:1383-89.
66. Silva A, Politti F, Novello A, Ferreira C, Rabelo N, Akalan NE, Lucareli P. Kinematic sensitivity and specificity to detect differences between women with patellofemoral pain and healthy women during the lateral step down test? Gait and Posture. 2017;57:291-92.
67. Stalter B, Holliday R, Van Zant S. Core strength assessment in athletes, ACL-injured, and control subjects: Implications for ACL injury risk. Medicine and Science in Sports and Exercise. 2006;38:S297.
68. Staples JR, Schafer KA, Smith MV, Motley J, Halstead M, Blackman A, Haas A, Steger-May K, Matava MJ, Wright RW, Brophy RH. Decreased postural control in patients undergoing anterior cruciate ligament reconstruction compared to healthy controls. Journal of Sport Rehabilitation. 2020;29:920-25.
69. Stensdotter A, Guerra JB, Hager-Ross C. Limb support in response to balance provocations in women with patellofemoral pain. Advances in Physiotherapy. 2009;11:97-103.
70. Tecco S, Colucci C, Calvisi V, Orso CA, Salini V, Festa F. Influence of knee pathology on body posture and muscle activity of head, neck and trunk muscles. Minerva Stomatologica. 2005;54:611-33.
71. Tecco S, Salini V, Calvisi V, Colucci C, Orso CA, Festa F, D’Attillio M. Effects of anterior cruciate ligament (ACL) injury on postural control and muscle activity of head, neck and trunk muscles. Journal of oral rehabilitation. 2006;33:576-87.
72. Tecco S, Salini V, Teté S, Festa F. Effects of Anterior Cruciate Ligament (ACL) injury on muscle activity of head, neck and trunk muscles: A cross-sectional evaluation. Cranio - Journal of Craniomandibular Practice. 2007;25:177-85.
73. Tengman E, Grip H, Stensdotter AK, Hager CK. Anterior cruciate ligament injury about 20 years post-treatment: A kinematic analysis of one-leg hop. Scandinavian Journal of Medicine & Science in Sports. 2015;25:818-27.
74. Ukishiro K, Ohkoshi Y, Ino T, Kawakami K, Suzuki S, Miura K, Ohsumi Y, Kotake S, Yoshida T, Maeda T, Suzuki K. Kinematics and kinetics of modular unlinked bicompartmental knee arthroplasty for medial and patellofemoral compartment arthritis. Journal of Orthopaedic Research. 2017;35.
75. Van Criekinge T, De Grave PW, Luyckx T, Claeys K. Does hamstrings strength determine trunk control after total knee arthroplasty? Gait and Posture. 2021;90:283-84.
76. Viggiano D, Corona K, Cerciello S, Vasso m, Schiavone-Panni A. The kinematic control during the backward gait and knee proprioception: Insights from lesions of the anterior cruciate ligament. Journal of Human Kinetics. 2014;41:51-57.
77. Wada S, Murakami H, Tajima G, Maruyama M, Sugawara A, Oikawa S, Chida Y, Doita M. Analysis of characteristics required for gait evaluation of patients with knee osteoarthritis using a wireless accelerometer. The Knee. 2021;32:37-45.
78. Werner DM, Barrios JA. Trunk muscle endurance in individuals with and without a history of anterior cruciate ligament reconstruction. Journal of Strength and Conditioning Research. 2021;35:118-23.
79. Willson JD. Hip strength and lower extremity mechanics in females with and without patellofemoral pain. University of Delaware [PhD Thesis]. 2007.
80. Willson JD, Binder-Macleod S, Davis IS. Lower extremity jumping mechanics of female athletes with and without patellofemoral pain before and after exertion. American Journal Sports Medicine. 2008;36:1587–96.
81. Willson JD, Davies IS. Lower extremity strength and mechanics during jumping in women with patellofemoral pain. Journal of Sport Rehabilitation. 2009;18:76-90.
82. Yamazaki J, Muneta T, Ju YJ, Sekiya I. Differences in kinematics of single leg squatting between anterior cruciate ligament-injured patients and healthy controls. Knee Surgery, Sports Traumatology, Arthroscopy. 2010;18:56-63.
83. Zazulak BT, Hewett TE, Reeves NP, Goldberg B, Cholweicki J. Deficits in neuromuscular control of the trunk predict knee injury risk: A prospective biomechanical-epidemiologic study. The American Journal of Sports Medicine. 2007;35:1123-30.

**Additional file 1C** Summary of included studies

| **Study** | **Knee disorders** | **Participants** | | **Task** | **Measurement method** | **Outcomes** | **Results summary** |
| --- | --- | --- | --- | --- | --- | --- | --- |
| Alvim et al. 2019 | PFP | **PFP group = 11**  M/F = 0/11  Range (Age) = NR (23.5±2 years)  Body mass = 55.4±4.9 Kg  Height = 166±4 cm  BMI = NR | **Control group = 10**  M/F = 0/10  Range (Age) = NR (23.2±4 years)  Body mass = 59.3±5.8 Kg  Height = 163±6 cm  BMI = NR | Single leg triple hop test (jumping) | 3D kinematics | Trunk flexion/extension (lumbar extension) (º), lateral trunk lean (lumbar bending) (º), trunk rotation (lumbar rotation) (º) | Greater trunk flexion (negative lumbar flexion) in those with PFP |
|  |  |  |  |  |  |  |  |
| Armitano et al. 2017 | ACLR | **ACLR group = 17**  M/F = NR  Range (Age) = NR (23.5±2.73 years)  Body mass = 77.4±13.7 Kg  Height = 172±9 cm  BMI = NR | **Control group = 17**  M/F = NR  Range (Age) = NR  (25±2.44 years)  Body mass = 75.7±14.9 Kg  Height = 173±10 cm  BMI = NR | Walking | 3D accelerometers | Acceleration patterns of lower trunk: time domains with root mean square of the acceleration signal (average acceleration amplitude); frequency domain with Welch’s average (frequency at which peak power frequency occurred); signal regularity with approximate entropy (degree of regularity of the accelerometer signals); and segmental gain (estimation of the degree of attenuation or gain between the trunk-neck and neck-head combinations) with a transfer function to RMS, frequency, and approximate entropy data | No significant differences between groups were found for RMS acceleration, approximate entropy and segmental gain in the antero-posterior, medio-lateral and vertical directions of the trunk. A diminished ability to attenuate frequency oscillations in the antero-posterior and medio-lateral directions were exhibited by the ACLR group compared to the control group |
|  |  |  |  |  |  |  |  |
| Asay et al. 2009 | Knee OA^+^  [KL 1-4] | **OA group = 23**  M/F = 8/15  Range (Age) = NR (61.8±7.3)  Body mass = 71.9±15.2 Kg  Height = 166±9 cm  BMI = NR  **Less severe OA group = 12**  M/F = 3/9  Range (Age) = NR (60.6±7 years)  Body mass = 72±15.1 Kg  Height = 166±7 cm  BMI = NR  **More severe OA group = 11**  M/F = 5/6  Range (Age) = NR (63.2±7.8 years)  Body mass = 71.9±16.1 Kg  Height = 166±10 cm  BMI = NR | **Control group = 20**  M/F = 9/11  Range (Age) = NR (53.7±12 years)  Body mass = 72.3±12.6 Kg  Height = 170±13 cm  BMI = NR | Stair ascent | 3D kinematics | Trunk flexion (forward trunk lean), during stance phase (°) | Individuals with more severe knee OA had greater trunk flexion than controls. No differences were found between those patients with less severe knee OA and controls |
|  |  |  |  |  |  |  |  |
| Azma et al. 2015 | Knee OA  [KL NR] | **OA group = 16**  M/F = 0/16  Range (Age) = NR (NR)  Body mass = 82.3±20 Kg  Height = 158±11 cm  BMI = 27.4±5.6 Kg/m^2^ | **Control group = 10**  M/F = 0/10  Range (Age) = NR (NR)  Body mass = 69.8±11 Kg  Height = 161±9 cm  BMI = 24±3.2 Kg/m^2^ | Walking | 3D kinematics | Lateral trunk lean at first and second peaks vertical ground reaction force, and midstance phases (°) | No significant differences between groups were found |
|  |  |  |  |  |  |  |  |
| Baellow et al. 2020 | PFP | **PFP group = 15**  M/F = 0/15  Range (Age) = NR (22.3±3.4)  Body mass = 65.6±13.7 Kg  Height = 166±6 cm  BMI = NR | **Control group = 15**  M/F = 0/15  Range (Age) = NR (20.3±1.3)  Body mass = 67.7±9.5 Kg  Height = 169±5 cm  BMI = NR | Drop vertical jump | 3D kinematics | Trunk flexion (º); lateral trunk lean (lateral trunk flexion) (º); Trunk rotation (º); time to peak for trunk flexion, lateral lean and rotation (s) | No significant differences between groups were found for trunk kinematics excursion in the sagittal, frontal and transverse planes. PFP group took less time to reach peak lateral trunk lean than the control group |
|  |  |  |  |  |  |  |  |
| Barker-Davies et al. 2019 | PT | **PT group = 21**  M/F = 21/0  Range (Age) = 18-55 (30.5±5.9 years)  Body mass = 88.4±10.8 Kg  Height = 178±8 cm  BMI = 27.7±3.2 Kg/m^2^ | **Control group = 22**  M/F = 22/0  Range (Age) = 18-55 (34±6.5 years)  Body mass = 85.3±9.2 Kg  Height = 179±6 cm  BMI = 26.8±2.7 Kg/m^2^ | Small knee bend, single leg squat and single leg decline squat | 3D kinematics | Trunk flexion at peak knee flexion (°) | No significant differences between groups were found for trunk flexion during all tasks |
|  |  |  |  |  |  |  |  |
| Bazett-Jones et al. 2013 | PFP | **PFP group = 19**  M/F = 10/9  Range (Age) = 18-40 (26±5.5 years)  Body mass = 77.3±14 Kg  Height = 174±8 cm  BMI = NR | **Control group = 19**  M/F = 10/9  Range (Age) = 18-40 (24.3±4.3 years)  Body mass = 70.2±11.3 Kg  Height = 174±9 cm  BMI = NR | Running | 3D kinematics | Trunk flexion during stance phase (°), ipsilateral and contralateral trunk lean (trunk bending) during stance phase (°) | No significant differences between groups were found for trunk kinematics in sagittal and frontal planes before the fatigue protocol |
|  |  |  |  |  |  |  |  |
| Bechard et al. 2012 | Knee OA  [KL 2-4] | **OA group = 20**  M/F = 12/8  Range (Age) = 44-72 (55±8 years)  Body mass = 87.2±11.8 Kg  Height = 174±10 cm  BMI = 28.9±3 Kg/m^2^ | **Control group = 20**  M/F = 8/12  Range (Age) = 37-62 (51±8 years)  Body mass = 78.3±12.4 Kg  Height = 174±6 cm  BMI = 25.9±3.2 Kg/m^2^ | Walking | 3D kinematics | Lateral trunk lean (maximum lateral trunk lean), relative to the pelvis coordinates system (°) at initial contact | Individuals with knee OA walked with greater ipsilateral trunk lean than controls |
|  |  |  |  |  |  |  |  |
| Bell et al. 2014 | ACLR | **Bone-patellar tendon-bone group = 34**  M/F = 5/29  Range (Age) = 18-25 (19.2±1.8 years)  Body mass = 69.59±11.6Kg  Height = 169±7 cm  BMI = NR  **Ipsilateral semitendinous and gracilis autograph group = 21**  M/F = 0/21  Range (Age) = 18-25 (19.1±1.7 years)  Body mass = 67.3±13.1 Kg  Height = 166±6 cm  BMI = NR  **ACLR group = 55**  M/F = 12/8  Range (Age) = 18-25 (NR)  Body mass = NR  Height = NR  BMI = NR | **Control group = 51**  M/F = 7/44  Range (Age) = 18-25 (19.8±1.4 years)  Body mass = 63.5±8.6 Kg  Height = 168±7 cm  BMI = NR | Single leg squat | Electromagnetic tracking sensors | Trunk flexion (forward trunk flexion) (°) and lateral trunk lean at peak knee flexion (lateral trunk flexion) (°) | The semitendinous and gracilis autograph group performed the task with more trunk flexion and ipsilateral trunk lean compared to the bone-patellar tendon-bone group and control group |
|  |  |  |  |  |  |  |  |
| Bjerke et al. 2014 | TKA | **TKA group = 23**  M/F = 11/12  Range (Age) = NR (57.6±5.8 years)  Body mass = 88±15.1 Kg  Height = 172±0.1 cm  BMI = 29.9±5 Kg/m^2^ | **Control group = 23**  M/F = 10/13  Range (Age) = NR (54.7±7.4 years)  Body mass = 74.3±15.5 Kg  Height = 173±0.1 cm  BMI = 24.5±3.1 Kg/m^2^ | Stair ascent | 3D kinematics | Trunk flexion (forward trunk lean) (º), relative to the pelvis coordinates system | No significant differences between groups were found for trunk flexion |
|  |  |  |  |  |  |  |  |
| Bley et al. 2014 | PFP | **PFP group = 20**  M/F = 0/20  Range (Age) = 18-35 (23.5±2.1 years)  Body mass = 55.3±4.8 Kg  Height = 165±4 cm  BMI = 20.2±1.8 Kg/m^2^ | **Control group = 20**  M/F = 0/20  Range (Age) = 18-35 (23.1±3.3 years)  Body mass = 55.9±7.1 Kg  Height = 162±6 cm  BMI = 21.3±2.7 Kg/m^2^ | Single leg triple hop test (jumping) | 3D kinematics | Trunk flexion (°) and lateral trunk lean (°) | Individuals with PFP showed greater trunk flexion and ipsilateral trunk lean than controls |
|  |  |  |  |  |  |  |  |
| Boekesteijn et al. 2021 | Knee OA  [KL 3-4] | **OA group = 25**  M/F = 12/13  Range (Age) = 61-67 (64±NR years)  Body mass = 84±NR Kg  Height = 172±NR cm  BMI = 28.5±NR Kg/m^2^ | **Control group = 27**  M/F = 13/14  Range (Age) = 63-68 (66±NR years)  Body mass = 76±NR Kg  Height = 172±NR cm  BMI = 25.7±NR Kg/m^2^ | Walking | Inertial sensor system | Trunk flexion (º), lateral trunk lean (º) and trunk rotation (°) | Individuals with knee OA showed greater lateral trunk lean compared to controls. No significant differences between groups were found for trunk kinematics in the sagittal and transverse plane |
|  |  |  |  |  |  |  |  |
| Boggess et al. 2018 | ACLR | **ACLR group = 11**  M/F = NR  Range (Age) = NR (22.7±3.5 years)  Body mass = 60.6±8.5 Kg  Height = 168±8 cm  BMI = NR | **Control group = 11**  M/F = NR  Range (Age) = NR (19.7±3.7 years)  Body mass = 64.2±12 Kg  Height = 167±6 cm  BMI = NR | Running | 3D kinematics and kinetics | Lateral trunk lean (trunk-pelvis lumbar bending) (°), trunk extension (trunk-pelvis lumbar extension) (°) during stance phase | No significant differences between groups were found for ipsilateral trunk lean and for trunk extension |
|  |  |  |  |  |  |  |  |
| Boonstra et al. 2008 | TKA | **TKA group = 28**  M/F = NR/60%  Range (Age) = NR (65.5±8.9 years)  Body mass = NR  Height = NR  BMI = 29.7±5.2 Kg/m^2^ | **Control group = 31**  M/F = NR/60%  Range (Age) = NR (65.4±8.6 years)  Body mass = NR  Height = NR  BMI = 28.4±3.8 Kg/m^2^ | Sit-to-stand | 3D accelerometers | Trunk flexion (forward flexion of the upper body) (°) and trunk angular velocity (upper body angular extension velocity) (°/s) | No significant differences between groups were found for trunk flexion and trunk angular velocity |
|  |  |  |  |  |  |  |  |
| Bramah et al. 2018 | PFP | **PFP group = 18**  M/F = NR  Range (Age) = NR (34.5±9.4 years)  Body mass = 64.4±9.6 Kg  Height = 173±8 cm  BMI = 21.3±1.9 Kg/m^2^ | **Control group = 36**  M/F = 15/21  Range (Age) = NR (33.2±8.4 years)  Body mass = 60.8±8.4 Kg  Height = 171±7 cm  BMI = 20.6±1.8 Kg/m^2^ | Running | 3D kinematics | Trunk flexion (trunk forward lean) (°) and lateral trunk lean (°) at initial contact; and at midstance phase | No significant differences between groups were found for trunk flexion and for ipsilateral trunk lean |
|  |  |  |  |  |  |  |  |
| Claudon et al. 2012 | PFP | **PFP group = 23**  M/F = 11/12  Range (Age) = NR (32.5±15.3 years)  Body mass = 75.6±12.9 Kg  Height = 172±5 cm  BMI = 25.6±4.6 Kg/m^2^ | **Control group = 22**  M/F = 10/12  Range (Age) = NR (24.9±6.5 years)  Body mass = 66.4±9.7 Kg  Height = 173±10 cm  BMI = 22.2±2.4 Kg/m^2^ | Walking | 3D kinematics | Trunk flexion (trunk forward bending) at peak knee extensor moment (°) | PFP group showed greater trunk flexion compared to the control group |
|  |  |  |  |  |  |  |  |
| Creaby et al. 2012 | Knee OA^++^  [KL 2-4] | **Uni-pain/Uni-x-ray group = 11**  M/F = 6/5  Range (Age) = >50 (64.5±7.6 years)  Body mass = 83.4±18.9 Kg  Height = 163±11 cm  BMI = 31.1±3.8 Kg/m^2^  **Uni-pain/Bi-x-ray group = 22**  M/F = 10/12  Range (Age) = >50 (65.1±9.4 years)  Body mass = 74.4±16.5 Kg  Height = 166±10 cm  BMI = 26.8±5 Kg/m^2^  **Bi-pain/Bi-x-ray group = 56**  M/F = 29/24  Range (Age) = >50  (64.5±8 years)  Body mass = 78.6±14.8 Kg  Height = 168±8 cm  BMI = 27.9±4.1 Kg/m^2^ | **Control group = 31**  M/F = 11/20  Range (Age) = >50  (63.8±8 years)  Body mass = 71.3±12.6 Kg  Height = 167±9 cm  BMI = 25.5±3.7 Kg/m^2^ | Walking | 3D kinematics | Lateral trunk lean (trunk lean toward the ipsilateral limb) (°) | Ipsilateral trunk lean was greater in all knee OA groups compared to the control group |
|  |  |  |  |  |  |  |  |
| Davies et al. 2018 | ACLR and ACLD | **ACLR group = 30**  M/F = 21/9  Range (Age) = 18-50 (30.5±9.7 years)  Body mass = 80±10 Kg  Height = 174±7 cm  BMI = 26.5±3.6 Kg/m^2^  **ACLD group = 28**  M/F = 23/5  Range (Age) = 18-50 (31±7.8 years)  Body mass = 81.9±14.8 Kg  Height = 178±9 cm  BMI = 25.9±4 Kg/m^2^ | **Control group = 32**  M/F = 22/10  Range (Age) = 18-50 (27.5±6.8 years)  Body mass = 75.3±17.3 Kg  Height = 174±11 cm  BMI = 24.6±3.6 Kg/m^2^ | Walking, running, single leg squat and single leg hop for distance | 3D kinematics | Lateral thorax lean (thorax obliquities) (°) during stance phase of walking and running, landing phase of single leg hop for distance and entire single leg squat movement | Significant differences found for individuals with ACLD during landing phase of the single leg hop for distance moving through a smaller range of lateral thorax lean (thorax obliquities) than controls; which was reported as a minimal frontal plane motion different from the control group |
|  |  |  |  |  |  |  |  |
| Dos Reis et al. 2015 | PFP | **PFP group = 20**  M/F = 0/20  Range (Age) = 18-35 (23.5±2.1 years)  Body mass = 55.3±4.8 Kg  Height = 171±13 cm  BMI = 20.2±1.8 Kg/m^2^ | **Control group = 20**  M/F = 0/20  Range (Age) = 18-35 (23.1±3.3 years)  Body mass = 55.9±7.1 Kg  Height = 165±12 cm  BMI = 21.3±2.7 Kg/m^2^ | Single leg triple hop test | 3D kinematics | Trunk flexion (°) (anterior trunk lean), lateral trunk lean (°) and trunk rotation (°) and time to peak angles (% of weight-bearing phase) for the weight-bearing period between the first and second hops (from initial contact to toe-off phases) | PFP group exhibited greater trunk flexion and ipsilateral trunk lean, but less ipsilateral trunk rotation than the control group. PFP group also exhibited a faster time to peak contralateral trunk rotation and a slower time to peak trunk flexion and ipsilateral trunk lean |
|  |  |  |  |  |  |  |  |
| Fenner et al. 2014 | TKA | **TKA group = 18**  M/F = 10/8  Range (Age) = NR (67.8±8.1 years)  Body mass = 73.4±10.9 Kg  Height = 168±9 cm  BMI = 25.8±2.9 Kg/m^2^ | **Control group = 20**  M/F = 12/8  Range (Age) = NR (66.1±6.4 years)  Body mass = 69.9±6.4. Kg  Height = 169±8 cm  BMI = 24.5±2.6 Kg/m^2^ | Walking and stair ascent | 3D kinematics | Trunk flexion (maximum anterior trunk tilt, anterior trunk tilt at initial contact, range of motion of trunk tilt during stance phase) (º), lateral trunk lean (initial trunk lean, maximum ipsilateral and contralateral lean) (º) and trunk rotation (initial contralateral trunk rotation, maximum ipsilateral trunk rotation, range of motion of trunk rotation during stance phase) (º) | Greater trunk flexion during stair ascent in TKA group compared to the control group. Greater ipsilateral trunk rotation during stance phase of walking and stair ascent in TKA group compared to the control group. No differences between groups for lateral trunk lean were found |
|  |  |  |  |  |  |  |  |
| Fenner et al. 2017 | TKA | **TKA group = 15**  M/F = 7/8  Range (Age) = NR (66.8±7.4 years)  Body mass = 73.3±10.7 Kg  Height = 168±9 cm  BMI = 25.9±2.8 Kg/m^2^ | **Control group = 17**  M/F = 10/7  Range (Age) = NR (66.6±6.8 years)  Body mass = 70.6±8.9 Kg  Height = 168±8 cm  BMI = 25±2.2 Kg/m^2^ | Walking and stair descent | 3D kinematics | Trunk flexion (maximum anterior trunk tilt, range of motion of trunk tilt during stance phase) (º), lateral trunk lean (maximum ipsilateral and contralateral lean) (º) and trunk rotation (maximum ipsilateral trunk rotation, range of motion of trunk rotation during stance phase) (º) | Greater ipsilateral trunk rotation during walking in TKA group compared to the control group. Individuals in TKA group also performed stair descend task with a more extension of the trunk compared to the control group. No differences between groups were found for lateral trunk lean |
|  |  |  |  |  |  |  |  |
| Fryer et al. 2019 | ACLR | **ACLR group = 130**  M/F = 36/94  Range (Age) = 13-26 (17.3±2.4 years)  Body mass = NR  Height = NR  BMI = NR | **Control group = 56**  M/F = 15/41  Range (Age) = 11-23 (17.2±2.5 years)  Body mass = NR  Height = NR  BMI = NR | Single leg landing task (landing phase) | 3D kinematics | Lateral trunk lean (frontal plane trunk excursion) | Greater ipsilateral lean (toward the involved limb) in individuals who have undergone ACLR compared to controls |
|  |  |  |  |  |  |  |  |
| Fu et al. 2021 | Knee OA  [KL 2-3] | **OA group = 30**  M/F = 4/26  Range (Age) = NR (58.6±5.6 years)  Body mass = 59.1±7.5 Kg  Height = 160±5 cm  BMI = 23±2.5 Kg/m^2^ | **Control group = 30**  M/F = 8/22  Range (Age) = NR (59.3±5.1 years)  Body mass = 59±9.6 Kg  Height = 160±6 cm  BMI = 22.8±2.4 Kg/m^2^ | 30-s Chair Stand Test | 3D kinematics | Trunk flexion (º) | No significant differences between groups were found for trunk kinematics in the sagittal plane |
|  |  |  |  |  |  |  |  |
| Fukuda et al. 2021a | ACLD | **ACLD group = 56**  M/F = NR  Range (Age) = NR (19.4±5.1 years)  Body mass = 61.6±11 Kg  Height = 166±7 cm  BMI = NR | **Control group = 46**  M/F = NR  Range (Age) = NR (22.9±2.9 years)  Body mass = 58.2±10.2 Kg  Height = 163±7 cm  BMI = NR | Single leg squat | 3D kinematics | Lateral trunk lean (º) | No significant differences between groups were found for trunk kinematics in the frontal plane |
|  |  |  |  |  |  |  |  |
| Fukuda et al. 2021b | ACLR | **ACLR group = 54**  M/F = NR  Range (Age) = NR (21.1±6.7 years)  Body mass = 61.7±10.9 Kg  Height = 165±8 cm  BMI = NR | **Control group = 44**  M/F = NR  Range (Age) = NR (22.6±1.4 years)  Body mass = 59.5±10.1 Kg  Height = 164±7 cm  BMI = NR | Single leg hop test  (landing phase) | 3D kinematics | Trunk flexion (º), lateral trunk lean (º) and trunk rotation (º) | No significant differences between groups were found for trunk kinematics in the sagittal and frontal planes. Lower trunk rotation in individuals with ACLR was found compared to controls |
|  |  |  |  |  |  |  |  |
| Glaviano et al. 2019 | PFP | **PFP group = 16**  M/F = 0/16  Range (Age) = NR (21.7±4.5 years)  Body mass = 67±13.9 Kg  Height = 166±5.9 cm  BMI = NR | **Control group = 9**  M/F = 0/9  Range (Age) = NR  (20.8±1.4 years)  Body mass = 66.6±8.6. Kg  Height = 168±6.5 cm  BMI = NR | Single leg squat | 3D kinematics | Trunk flexion (º) and lateral trunk lean (º) | Greater ipsilateral trunk lean in those with PFP presenting with elevated fear avoidance beliefs compared to controls |
|  |  |  |  |  |  |  |  |
| Haghighat et al. 2021 | PFP | **PFP group = 17**  M/F = 0/17  Range (Age) = 18-35 (25.9±3.9 years)  Body mass = 59.7±10.8 Kg  Height = 163±5 cm  BMI = NR | **Control group = 17**  M/F = 0/17  Range (Age) = 18-35 (24.1±3.9 years)  Body mass = 56.3±5.7 Kg  Height = 161±6 cm  BMI = NR | Running | 3D kinematics | Trunk flexion (forward flexion) (°) and lateral trunk lean (lateral flexion toward the stance leg) (°) | No significant differences between groups were found for trunk kinematics in the sagittal and frontal planes |
|  |  |  |  |  |  |  |  |
| Hálfdanardóttir et al. 2018 | Knee OA  [KL 2-3] | **OA group = 17**  M/F = 17/0  Range (Age) = 40-59 (50.4±6.2 years)  Body mass = NR  Height = NR  BMI = 28.3±3.1 Kg/m^2^ | **Control group = 14**  M/F = 14/0  Range (Age) = 40-65 (49.7±7.4 years)  Body mass = NR  Height = NR  BMI = 27.1±7.4 Kg/m^2^ | Walking | 3D kinematics | Lateral trunk lean (°) | Controls initiated the initial contact with greater lateral trunk lean towards the stance limb. Controls also showed an earlier transition (17% of stance phase) to more trunk lean away the stance limb indicating a delayed transition (21% of stance phase) for OA group |
|  |  |  |  |  |  |  |  |
| Hart et al. 2015 | Knee OA  [KL 1-4] | **OA group = 19**  M/F = 79%/21%  Range (Age) = NR (37±7 years)  Body mass = 80±10 Kg  Height = 172±6 cm  BMI = 27±3 Kg/m^2^ | **Control group = 25**  M/F = 56%/44%  Range (Age) = 18-40 (31±6 years)  Body mass = 69±11 Kg  Height = 173±10 cm  BMI = 23±2 Kg/m^2^ | Walking | 3D kinematics | Trunk flexion (°), lateral trunk lean (lateral bend) (°) and trunk rotation (axial rotation) during stance phase (°) | No significant differences between groups were found for trunk kinematics in the sagittal, frontal and transverse planes |
|  |  |  |  |  |  |  |  |
| Hébert et al. 1994 | PFP | **PFP group (female) = 9**  Range (Age) = NR (21±3.7 years)  Body mass = 60±4.6 Kg  Height = 160±4 cm  BMI = NR  **PFP group (male) = 2**  Range (Age) = NR (27.5±4.9 years)  Body mass = 88.9±21.2 Kg  Height = 180±5 cm  BMI = NR  **PFP group = 11**  M/F = 2/9  Range (Age) = NR (NR)  Body mass = NR  Height = NR  BMI = NR | **Control group (female) = 7**  Range (Age) = NR (24.3±2.9 years)  Body mass = 54.5±6.3 Kg  Height = 170±5 cm  BMI = NR  **Control group (male) = 4**  Range (Age) = NR (25±3.6 years)  Body mass = 72.6±8.2 Kg  Height = 170±6 cm  BMI = NR  **Control group = 11**  M/F = 4/7  Range (Age) = NR (NR)  Body mass = NR  Height = NR  BMI = NR | Squatting | 2D kinematics | Trunk flexion displacement (°) in three different squat techniques (natural, imposed and tip toes) | No significant differences between groups were found for trunk flexion displacement |
|  |  |  |  |  |  |  |  |
| Hewett et al. 2009 | ACLD | **ACLD group = 17**  M/F = 7/10  Range (Age) = NR (NR)  Body mass = NR  Height = NR  BMI = NR | **Control group = 6**  M/F = 0/6  Range (Age) = NR (NR)  Body mass = NR  Height = NR  BMI = NR | Landing and cutting tasks | 2D kinematics | Trunk flexion displacement (°) and lateral trunk lean displacement (°) at initial contact, 50 ms, 100 ms, 150 ms and 200 ms post contact | Greater ipsilateral trunk lean and lower trunk flexion displacements in ACL-deficient women compared to injury-free women at initial contact |
|  |  |  |  |  |  |  |  |
| Ho et al. 2021 | PFP | **PFP group = 7**  M/F = 2/5  Range (Age) = 18-40 (28±8.2 years)  Body mass = 65.2±12.9 Kg  Height = 172±12 cm  BMI = 21.7±2.6 Kg/m^2^ | **Control group = 5**  M/F = 2/3  Range (Age) = 18-40 (27.4±7.8 years)  Body mass = 75.7±12.6 Kg  Height = 175±8.9 cm  BMI = 24.4±2.6 Kg/m^2^ | Running | 3D kinematics | Trunk flexion (°) during stance phase | No significant differences between groups were found for trunk kinematics in the sagittal plane |
|  |  |  |  |  |  |  |  |
| Hunt et al. 2010 | Knee OA^†^  [KL 2-4] | **Mild OA group = 25**  M/F = 10/15  Range (Age) = NR (61.2±7.7 years)  Body mass = 73.7±14 Kg  Height = 165±8 cm  BMI = NR  **Moderate OA group = 25**  M/F = 14/11  Range (Age) = NR (63.6±8.4 years)  Body mass = 78.4±14.8 Kg  Height = 167±8 cm  BMI = NR  **Severe OA group = 25**  M/F = 13/12  Range (Age) = NR (68±6.6 years)  Body mass = 81.7±20.2 Kg  Height = 168±9 cm  BMI = NR  **OA group = 75**  M/F = 37/38  Range (Age) = NR (NR years)  Body mass = NR  Height = NR  BMI = NR | **Control group = 20**  M/F = 5/15  Range (Age) = NR (63.2±12.4 years)  Body mass = 69.3±12.1 Kg  Height = 165±6 cm  BMI = NR | Walking | 3D kinematics | Lateral trunk lean (°) during stance phase | Individuals with severe knee OA exhibited significantly more ipsilateral trunk lean than controls and those with mild and moderate knee OA. Also, individuals with moderate knee OA exhibited significantly more contralateral trunk lean (lean toward the non-tested limb) compared to controls and individuals with severe knee OA |
|  |  |  |  |  |  |  |  |
| Kean et al. 2013 | Knee OA  [KL 2-4] | **OA group = 30**  M/F = 13/17  Range (Age) = ≥40 (63.3±9.7 years)  Body mass = NR  Height = NR  BMI = 28.6±3.6 Kg/m^2^ | **Control group = 30**  M/F = 14/16  Range (Age) = ≥40 (55.3±8.9 years)  Body mass = NR  Height = NR  BMI = 28.7±2.6 Kg/m^2^ | Walking | 3D kinematics | Lateral trunk lean (°) at time of peak knee adduction moment | No significant changes were found for lateral trunk lean |
|  |  |  |  |  |  |  |  |
| Kotsifaki et al. 2021a | ACLR | **ACLR group = 26**  M/F = 26/0  Range (Age) = 18-35 (23.2±3.4 years)  Body mass = 71.4±12.1 Kg  Height = 173±NR cm  BMI = 23.3±2.3 Kg/m^2^ | **Control group = 23**  M/F = 23/0  Range (Age) = 18-35 (28.3±4.4 years)  Body mass = 76.1±7.4 Kg  Height = 178±6.9 cm  BMI = 23.9±1.6 Kg/m^2^ | Single leg hop for distance (landing phase) | 3D kinematics | Trunk flexion (°) | Greater trunk flexion was found in those with ACLR compared to controls |
|  |  |  |  |  |  |  |  |
| Kotsifaki et al. 2021b | ACLR | **ACLR group = 24**  M/F = 24/0  Range (Age) = 18-35 (23.4±3.4 years)  Body mass = 72.5±11.8 Kg  Height = 175±10 cm  BMI = 23.3±2.3 Kg/m^2^ | **Control group = 23**  M/F = 23/0  Range (Age) = 18-35 (28.3±4.4 years)  Body mass = 76.1±7.4 Kg  Height = 178±6 cm  BMI = 23.9±1.6 Kg/m^2^ | Single leg triple hop distance | 3D kinematics | Trunk flexion (º) during 2 rebounding phases (landing then jumping) and a final landing phase | Greater trunk flexion was found in those with ACLR compared to controls during the 2^nd^ rebound and final landing phases |
|  |  |  |  |  |  |  |  |
| Kotsifaki et al. 2022 | ACLR | **ACLR group = 26**  M/F = 26/0  Range (Age) = 18-35 (23.2±3.4 years)  Body mass = 71.4±12.1 Kg  Height**^‡^** = 173 (166-182) cm  BMI = 23.3±2.3 Kg/m^2^ | **Control group = 22**  M/F = 22/0  Range (Age) = 18-35 (28.7±3.8 years)  Body mass = 75.7±7.1 Kg  Height**^‡^** = 177.4 (6.1) cm  BMI = 24±1.6 Kg/m^2^ | Single leg vertical jump and single leg drop jump | 3D kinematics | Trunk flexion (º) during jumping and landing of single leg vertical jump and single leg drop jump | No significant differences between groups were found for trunk kinematics in the sagittal plane during single leg vertical jump. Greater trunk flexion was found in individuals with ACLR during single leg drop jump |
|  |  |  |  |  |  |  |  |
| Kuwahara et al. 2019 | Knee OA and TKA  [KL 3-4] | **OA and TKA group = 14**  M/F = 2/12  Range (Age) = NR (74.8±6.2 years)  Body mass = 58±9.2 Kg  Height = 152±5 cm  BMI = 25±3.7 Kg/m^2^ | **Control group = 11**  M/F = 5/6  Range (Age) = NR (74.4±6.1 years)  Body mass = 57.5±10.6 Kg  Height = 158±6 cm  BMI = 22.8±3.3 Kg/m^2^ | Walking | 3D kinematics | Trunk flexion (anterior tilt) (°) during stance phase | Greater trunk flexion was found in those with knee OA compared to controls. No significant differences between groups were found for trunk flexion |
|  |  |  |  |  |  |  |  |
| Lessi and Serrão 2017 | ACLR | **ACLR group = 20**  M/F = 13/7  Range (Age) = 18-35 (25.1±4.2 years)  Body mass = 75.1±10.2 Kg  Height = 173±10 cm  BMI = NR | **Control group = 20**  M/F = 13/7  Range (Age) = 18-35 (23.6±2.9 years)  Body mass = 72.8±11.5 Kg  Height = 173±10 cm  BMI = NR | Single leg drop vertical jump (landing phase) | 3D kinematics | Trunk flexion (°) and lateral trunk lean (°) | No significant differences between groups were found for trunk flexion and lateral trunk lean considering analyses before fatigue |
|  |  |  |  |  |  |  |  |
| Li et al. 2013 | TKA | **TKA group = 14**  M/F = NR  Range (Age) = NR (67±7 years)  Body mass = 88.9±15.9 Kg  Height = 171±10 cm  BMI = NR | **Control group = 14**  M/F = NR  Range (Age) = NR (67±7 years)  Body mass = 80.5±10.2 Kg  Height = 169±8 cm  BMI = NR | Walking | 3D kinematics and kinetics | Trunk flexion (back flexion) (°) and net back extension moment (%BW*HT) during ipsilateral foot strike, contralateral toe-off, contralateral foot strike and ipsilateral toe-off | TKA individuals walked with significantly increased back flexion during all walking phases than controls. Also, back extension moment was greater in the TKA individuals than controls throughout stance, with significant differences in early stance |
|  |  |  |  |  |  |  |  |
| Linley et al. 2010 | Knee OA  [KL 1-4] | **OA group = 40**  M/F = 17/23  Range (Age) = ≥ 40 (63±10 years)  Body mass = 82.3±20 Kg  Height = 173±11 cm  BMI = 27.4±5.5 Kg/m^2^ | **Control group = 40**  M/F = 17/23  Range (Age) = ≥ 40 (64±9 years)  Body mass = 69.7±11 Kg  Height = 170±9 cm  BMI = 24±3.2 Kg/m^2^ | Walking | 3D kinematics | Lateral trunk lean (thoracic tilt in the frontal plane) (°) during 20-80% of the stance phase for the affected limb; lateral trunk lean (°) at first and second phases of stance; lateral trunk lean (°) at midstance (50%) | No significant differences between groups were found for lateral trunk lean |
|  |  |  |  |  |  |  |  |
| Magalhães et al. 2013 | Knee OA  [KL 2-3] | **OA group = 40**  M/F = 0/40  Range (Age) = NR (69.4±8 years)  Body mass = NR  Height = 154±5 cm  BMI = 31.3±5.6 Kg/m^2^ | **Control group = 40**  M/F = 0/40  Range (Age) = NR (70.1±8 years)  Body mass = NR  Height = 153±5 cm  BMI = 27.3±5.1 Kg/m^2^ | Walking | 3D kinematics | Lateral trunk lean (trunk inclination) (°) | Greater ipsilateral trunk lean was found in those with knee OA compared to controls |
|  |  |  |  |  |  |  |  |
| Markstrom et al. 2018 | ACLD and ACLR | **ACLD group = 34**  M/F = 21/13  Range (Age) = NR (47.6±5.9 years)  Body mass = NR  Height = NR  BMI = 28.7±4.3 Kg/m^2^  **ACLR group = 32**  M/F = 20/12  Range (Age) = NR (45.5±4.6 years)  Body mass = NR  Height = NR  BMI = 27.1±3.3 Kg/m^2^  **ACL group = 66**  M/F = 41/25  Range (Age) = NR (NR)  Body mass = NR  Height = NR  BMI = NR | **Control group = 33**  M/F = 22/11  Range (Age) = NR (46.7±5 years)  Body mass = NR  Height = NR  BMI = 24.6±2.5 Kg/m^2^ | One-leg vertical hop | 3D kinematics | Trunk flexion (trunk forward flexion) (°) during take-off and landing phases | ACLR group demonstrated greater trunk flexion than ACLD and control groups during take-off and landing phases |
|  |  |  |  |  |  |  |  |
| Markstrom et al. 2020 | ACLR | **ACLR group = 32**  M/F = 8/24  Range (Age) = 17-34 (24.1±4.5 years)  Body mass = 70.3±10.3 Kg  Height = 172±8 cm  BMI = NR | **Control group = 32**  M/F = 8/24  Range (Age) = 17-34 (22.9±3.1 years)  Body mass = 66.9±8.1 Kg  Height = 171±8 cm  BMI = NR | Standardized rebound side hop (landing phase) | 3D kinematics | Trunk flexion (°) and lateral trunk lean (lateral bending) (°) | ACLR group demonstrated greater trunk flexion than the control group during landing phase with the reconstructed limb |
|  |  |  |  |  |  |  |  |
| Markstrom et al. 2021 | ACLR | **ACLR group (high-fear) = 21**  M/F = 10/11  Range (Age) = 17-34 (24±3.9 years)  Body mass = 72.3±10.3 Kg  Height = 173±9 cm  BMI = 23.9±2.2 Kg/m^2^  **ACLR group (low-fear) = 17**  M/F = 8/9  Range (Age) = 17-34 (25.5±5.8 years)  Body mass = 74.6±12.2 Kg  Height = 176±8 cm  BMI = 24±3.2 Kg/m^2^  **ACLR group = 38**  M/F = 18/20  Range (Age) = 17-34 (NR)  Body mass = NR  Height = NR  BMI = NR | **Control group = 39**  M/F = 7/32  Range (Age) = 17-34 (22.4±3 years)  Body mass = 65.6±7.8 Kg  Height = 171±7 cm  BMI = 22.5±2 Kg/m^2^ | Standardized rebound side hop (landing phase) | 3D kinematics | Trunk flexion (°) and lateral trunk lean (lateral bending) (°) | ACLR group with high fear of reinjury demonstrated greater trunk flexion than controls during landing phase. No significant differences for trunk sagittal plane kinematics were found between ACLR group with low fear of reinjury and controls. No significant differences were found across groups for trunk frontal plane kinematics |
|  |  |  |  |  |  |  |  |
| Meireles et al. 2019 | Knee OA  [KL 2-3] | **OA group = 5**  M/F = 3/2  Range (Age) = NR (52.8±11 years)  Body mass = 83.8±14.8Kg  Height = 170±11 cm  BMI = NR | **Control group = 8**  M/F = 4/4  Range (Age) = NR (51±13.4 years)  Body mass = 74.1±13.7 Kg  Height = 166±10 cm  BMI = NR | Stair ascent and stair descent | 3D kinematics | Trunk flexion (°), lateral trunk lean (°) and trunk rotation (°) during step-over-step at controlled speed, step-over step at self-selected speed and step-by-step at self-selected speed^††^ | Individuals with knee OA had significantly greater trunk flexion during stair ascent than controls. No significant differences for the other variables were found |
|  |  |  |  |  |  |  |  |
| Nakagawa et al. 2012a | PFP | **PFP group (female) = 20**  Range (Age) = 18-35 (22.4±3.2 years)  Body mass = 61.6±7.8 Kg  Height = 166±6 cm  BMI = NR  **PFP group (male) = 20**  Range (Age) = 18-35 (23.5±3.2 years)  Body mass = 77.5±9.1 Kg  Height = 181±5 cm  BMI = NR  **PFP group = 40**  M/F = 20/20  Range (Age) = 18-35 (NR)  Body mass = NR  Height = NR  BMI = NR | **Control group (female) = 20**  Range (Age) = 18-35 (21.9±2.7 years)  Body mass = 58.5±7 Kg  Height = 163±5 cm  BMI = NR  **Control group (male) = 20**  Range (Age) = 18-35 (23.2±3.9 years)  Body mass = 75.3±10.1 Kg  Height = 178±5 cm  BMI = NR  **Control group = 40**  M/F = 20/20  Range (Age) = 18-35 (NR)  Body mass = NR  Height = NR  BMI = NR | Forward step-down task | 3D kinematics | Lateral trunk lean (maximum excursion of ipsilateral trunk lean) (°) at 15°, 30°, 45°, and 60° of knee flexion during the downward and upward phases | PFP group showed significant greater ipsilateral trunk lean compared to the control group (except at 15° and 30° of knee flexion in the downward phase) |
|  |  |  |  |  |  |  |  |
| Nakagawa et al. 2012b | PFP | **PFP group (female) = 20**  Range (Age) = 18-35 (22.3±3.1 years)  Body mass = 61.1±7.5 Kg  Height = 166±5 cm  BMI = NR  **PFP group (male) = 20**  Range (Age) = 18-35 (24.2±4.4 years)  Body mass = 77±9.6 Kg  Height = 180±5 cm  BMI = NR  **PFP group = 40**  M/F = 20/20  Range (Age) = 18-35 (NR)  Body mass = NR  Height = NR  BMI = NR | **Control group (female) = 20**  Range (Age) = 18-35 (21.8±2.6 years)  Body mass = 59.4±7.3 Kg  Height = 163±7 cm  BMI = NR  **Control group (male) = 20**  Range (Age) = 18-35 (23.5±3.8 years)  Body mass = 74.6±9.1 Kg  Height = 176±6 cm  BMI = NR  **Control group = 40**  M/F = 20/20  Range (Age) = 18-35 (NR)  Body mass = NR  Height = NR  BMI = NR | Single leg squat | 3D kinematics | Lateral trunk lean (maximum excursion of ipsilateral trunk lean) (°) | PFP group showed a significant greater ipsilateral trunk lean compared to the control group |
|  |  |  |  |  |  |  |  |
| Nakagawa et al. 2015 | PFP | **PFP group = 30**  M/F = 10/20  Range (Age) = 18-35 (22.7±3.4 years)  Body mass = 65.3±10.3 Kg  Height = 171±9 cm  BMI = NR | **Control group = 30**  M/F = 10/20  Range (Age) = 18-35 (22.3±3 years)  Body mass = 63.3±9.8 Kg  Height = 168±8 cm  BMI = NR | Single leg squat | 3D kinematics | Lateral trunk lean (°) | PFP group showed greater ipsilateral trunk lean compared to the control group |
|  |  |  |  |  |  |  |  |
| Noehren et al. 2012 | PFP | **PFP group = 16**  M/F = 0/16  Range (Age) = 18-45 (27±6 years)  Body mass = 57.4±4.6 Kg  Height = 164±5 cm  BMI = NR | **Control group = 16**  M/F = 0/16  Range (Age) = 18-45 (25±4 years)  Body mass = 58.7±6.5 Kg  Height = 165±7 cm  BMI = NR | Running | 3D kinematics | Lateral trunk lean (°) | No significant differences between groups were found for lateral trunk lean |
|  |  |  |  |  |  |  |  |
| Noehren et al. 2014 | ACLR | **ACLR group = 20**  M/F = 0/20  Range (Age) = 16-40 (21.1±5.9 years)  Body mass = NR  Height = NR  BMI = NR | **Control group = 20**  M/F = 0/20  Range (Age) = 16-40 (22.8±3.1 years)  Body mass = NR  Height = NR  BMI = NR | Running | 3D kinematics | Trunk flexion (°) (forward trunk lean) during the first 75% of stance phase and lateral trunk lean (°) at initial contact (trunk side bending) | ACLR group showed greater trunk lean towards the ipsilateral side and trunk flexion compared to the control group |
|  |  |  |  |  |  |  |  |
| Novello et al. 2018 | PFP | **PFP group = 34^‡^**  M/F = 0/34  Range (Age) = 18-35 (23 [20-31 years])  Body mass = 58 (52-62) Kg  Height = 160 (155-165) cm  BMI = 22.4 (20-24) Kg/m^2^ | **Control group = 34^‡^**  M/F = 0/34  Range (Age) = 18-35 (26 [23-28 years])  Body mass = 55 (51-61) Kg  Height = 161 (160-170) cm  BMI = 20.5 (19-23) Kg/m^2^ | Stair descent | 3D kinematics | Lateral trunk lean (°) at initial contact and load response; lateral trunk lean (°) range of motion (ROM) during stance phase | No significant differences were found between groups for lateral trunk lean |
|  |  |  |  |  |  |  |  |
| Nunes et al. 2020 | PFP | **PFP group = 28**  M/F = 0/28  Range (Age) = 18-35 (NR)  Body mass = NR  Height = NR  BMI = NR  **Experimental PFP group = 14**  M/F = 0/14  Range (Age) = 18-35 (24.3±2.7 years)  Body mass = 59.6±6.8 Kg  Height = 162±5 cm  BMI = NR  **Sham PFP group = 14**  M/F = 0/14  Range (Age) = 18-35 (24.1±5.1 years)  Body mass = 57.3±8.1 Kg  Height = 164±7 cm  BMI = NR | **Control group = 28**  M/F = 0/28  Range (Age) = 18-35 (NR)  Body mass = NR  Height = NR  BMI = NR  **Experimental control group = 14**  M/F = 0/14  Range (Age) = 18-35 (22.3±2.5 years)  Body mass = 60.3±7.9 Kg  Height = 166±6 cm  BMI = NR  **Sham control group = 14**  M/F = 0/14  Range (Age) = 18-35 (23.4±2.8 years)  Body mass = 59.5±5.9 Kg  Height = 163±6 cm  BMI = NR | Single leg squat and single leg drop vertical jump | 3D kinematics | Trunk flexion (°) and lateral trunk lean (trunk inclination) (°) at peak knee flexion | Descriptive data only |
|  |  |  |  |  |  |  |  |
| Oberlander et al. 2012 | ACLD | **ACLD group = 12**  M/F = NR  Range (Age) = NR (27±6 years)  Body mass = 83.2±12.6 kg  Height = 182±10 cm  BMI = NR | **Control group = 13**  M/F = NR  Range (Age) = NR (26±2 years)  Body mass = 79±7.2 Kg  Height = 182±8 cm  BMI = NR | Single leg hop test (landing phase) | 3D kinematics | Trunk flexion (°) | ACLD group showed more flexed trunk position during landing phase compared to the contralateral limb, but not to controls |
|  |  |  |  |  |  |  |  |
| Petrella et al. 2019 | Knee OA^††^  [KL 2-3] | **OA group = 41**  M/F = NR  Range (Age) = NR (NR years)  Body mass = NR  Height = NR  BMI = NR  **Mild OA group = 25**  M/F = 40%/60%  Range (Age) = 40-65 (56.4±5.3 years)  Body mass = 74.1±8.7 Kg  Height = 167±8 cm  BMI = 26.7±3.1 Kg/m^2^  **Moderate OA group = 16**  M/F = 50%  Range (Age) = 40-65 (56.8±6 years)  Body mass = 84.7±13.5 Kg  Height = 166±9 cm  BMI = 30.4±3.5 Kg/m^2^ | **Control group = 21**  M/F = 53%/47%  Range (Age) = 40-65 (55.8±6.5 years)  Body mass = 70.6±13.7 Kg  Height = 165±9 cm  BMI = 25.9±4.1 Kg/m^2^ | Sit-to-stand | 3D kinematics | Trunk flexion (°) during the phases of the task: Leaning Phase (1) - from the start of the trial to the maximum hip flexion; Momentum Phase (2) - from maximum  hip flexion to maximum ankle flexion; and Extension Phase (3) - from maximum dorsiflexion to the end of the trial | Greater trunk flexion in those with moderate knee OA during phases 2 and 3 of the task compared to controls |
|  |  |  |  |  |  |  |  |
| Pozzi et al. 2016 | TKA | **TKA group = 20**  M/F = 8/11  Range (Age) = 61-83 (70±6 years)  Body mass = 85.4±17.7 Kg  Height = 168±6 cm  BMI = 30±5 Kg/m^2^ | **Control group = 20**  M/F = 9/10  Range (Age) = 51-81 (67±8 years)  Body mass = 73.6±16.5 Kg  Height = 167±9 cm  BMI = 25.8±3.7 Kg/m^2^ | Step up and over task | 3D kinematics | Trunk flexion (°) at peak total support moment during propulsive, lowering and weight acceptance phases of the task | Greater trunk flexion in those with unilateral TKA may occur at each peak total support moment during the phases of the task compared to controls |
|  |  |  |  |  |  |  |  |
| Preece et al. 2019 | Knee OA  [KL NR] | **OA group = 27**  M/F = 17/10  Range (Age) = NR (56±9 years)  Body mass = NR  Height = NR  BMI = 28±3 Kg/m^2^ | **Control group = 19**  M/F = 13/6  Range (Age) = NR (54±11 years)  Body mass = NR  Height = NR  BMI = 27±3 Kg/m^2^ | Walking | 3D kinematics | Trunk flexion (°) and lateral trunk lean (°) | Greater trunk flexion in those with knee OA compared to controls. No significant differences were found between groups for lateral trunk lean |
|  |  |  |  |  |  |  |  |
| Preece and Alghamdi 2021 | Knee OA  [KL 1-4] | **OA group = 20**  M/F = 7/13  Range (Age) = NR (56±9 years)  Body mass = 81±14  Height = 170±7  BMI = 28.7±4.9 Kg/m^2^ | **Control group = 20**  M/F = 7/13  Range (Age) = NR (57±9 years)  Body mass = 80±11  Height = 170±6  BMI = 27.4±3.9 Kg/m^2^ | Walking | 3D kinematics | Trunk flexion (°) during 15-25% of stance phase | Individuals with knee OA walked with greater trunk flexion than controls |
|  |  |  |  |  |  |  |  |
| Saari et al. 2004 | TKA | **TKA group = 28^‡^**  M/F = 8/21  Age (range) = 61-80 years  Body mass (range) = 64-86 Kg  Height (range) = 162-170 cm  BMI (range) = 27.1-30.3 Kg/m^2^ | **Control group = 16^‡^**  M/F = 7/9  Age (median/range) = 69/50-87 years  Body mass (median/range) = 73/59-98 Kg  Height (median/range) = 166/150-180 cm  BMI (median/range) = 26.4/21.9-29.8 Kg/m^2^ | Rise and sit down on a chair | 3D kinematics | Trunk flexion (°) | No significant differences were found between groups for trunk flexion |
|  |  |  |  |  |  |  |  |
| Sagawa et al. 2017 | Knee OA  [KL 3-4] | **OA group = 101**  M/F = 45/56  Range (Age) = NR (NR years)  Body mass = NR  Height = NR  BMI = NR  **C-STS group = 24**  M/F = NR  Range (Age) = NR (NR)  Body mass = 83.5±18.1 Kg  Height = 168±10 cm  BMI = 29.3±5.2 Kg/m^2^  **IC-STS group = 59**  M/F = NR  Range (Age) = NR (NR)  Body mass = 83.6±16.7 Kg  Height = 165±10 cm  BMI = 31.8±5.9 Kg/m^2^  **SI-STS group = 18**  M/F = NR  Range (Age) = NR (NR years)  Body mass = 82.7±11.7 Kg  Height = 162±11 cm  BMI = 31.6±4.7 Kg/m^2^ | **Control group = 27**  M/F = NR  Range (Age) = NR (NR)  Body mass = 67.3±10.4 Kg  Height = 168±10 cm  BMI = 23.6±2.4 Kg/m^2^ | Sit-to-stand | 3D kinematics | Trunk flexion (trunk flexion max) (°) and lateral trunk lean (trunk obliquity max on the non-affected side) (°) | Knee OA groups performed the task with compensation strategies of the trunk kinematics in the sagittal and frontal plane (greater angular displacement) compared to the control group |
|  |  |  |  |  |  |  |  |
| Scarneo-Miller et al. 2019 | ACLR | **ACLR group = 23**  M/F = 14/9  Range (Age) = 13-40 (21±3 years)  Body mass = 76.2±9.9 Kg  Height = 174±7.2 cm  BMI = NR | **Control group = 23**  M/F = 14/9  Range (Age) = 13-40 (21±3 years)  Body mass = 75±10.5 Kg  Height = 173±9.2 cm  BMI = NR | Single leg squat and jump-cut | Electromagnetic tracking sensors | Trunk flexion (°), lateral trunk flexion (°) and trunk rotation during both tasks | Lower trunk flexion were reported in those who have undergone ACLR compared to controls during single leg squat and jump-cut tasks |
|  |  |  |  |  |  |  |  |
| Scattone et al. 2017 | PT | **PT group = 7**  M/F = 7/0  Range (Age) = 15-30 (18±1 years)  Body mass = 80.2±7.8 Kg  Height = 189±5 cm  BMI = NR | **Control group = 7**  M/F = 7/0  Range (Age) = 15-30 (16.2±1 years)  Body mass = 82.2±10.9 Kg  Height = 196±10 cm  BMI = NR | Bilateral drop landing | 3D kinematics | Trunk flexion (°) at foot contact and while landing (°) at self-selected, extended, and flexed trunk positions | Greater trunk flexion was showed in those with PT compared to controls |
|  |  |  |  |  |  |  |  |
| Schwane et al. 2015 | PFP | **PFP group = 20**  M/F = 0/20  Range (Age) = 18-35 (22.2±3.1 years)  Body mass = 63.5±13.6 Kg  Height = 164.5±9.2 cm  BMI = NR | **Control group = 20**  M/F = 0/20  Range (Age) = 18-35 (21±2.6 years)  Body mass = 63.8±12.7 Kg  Height = 164.5±7.1 cm  BMI = NR | Stair descent | 3D kinematics | Trunk flexion (°) lateral trunk lean (°) and trunk rotation (°) during the stance phase | No significant differences were found between groups for trunk kinematics in the sagittal, frontal, and transverse planes |
|  |  |  |  |  |  |  |  |
| Setuain et al. 2015a | ACLR | **ACLR group = 6**  M/F = 6/0  Range (Age) = NR (27.6±1.2 years)  Body mass = 92±3.4 Kg  Height = 188.5±2.3 cm  BMI = NR | **Control group = 16**  M/F = 16/0  Range (Age) = 18-35 (24.1±1.2 years)  Body mass = 89.8±2.4 Kg  Height = 188±1.8 cm  BMI = NR | Vertical jump test battery: 50 cm vertical bilateral drop jump, 20 cm vertical unilateral drop jump, and vertical unilateral countermovement jump | Single inertial sensor unit | Antero-posterior axis angular excursion (°), medio-lateral axis angular excursion (°) and vertical axis angular excursion (°) of the trunk during initial absorption, propulsive and final absorption phases of the 50 cm vertical bilateral drop jump and 20 cm vertical unilateral drop jump tests; and during propulsive and final absorption phases of the vertical unilateral countermovement jump test | Greater angular excursions around the vertical axis (Z-axis) for controls while performing a 20 cm vertical unilateral drop jump during all three pre-defined jumping phases. Greater medio-lateral (X-axis) trunk displacement in the final absorption phase of the vertical unilateral countermovement jump in those individuals who have undergone ACLR compared with controls |
|  |  |  |  |  |  |  |  |
| Setuain et al. 2015b | ACLR | **ACLR group = 6**  M/F = 0/6  Range (Age) = NR (26.4±1.4 years)  Body mass = 61.8±1.4 Kg  Height = 169±1.6 cm  BMI = NR | **Control group = 15**  M/F = 15/0  Range (Age) = 18-35 (25.1±1.4 years)  Body mass = 69.5±1.8 Kg  Height = 175±1.4 cm  BMI = NR | Vertical jump test battery: 50 cm vertical bilateral drop jump, 20 cm vertical unilateral drop jump, and vertical unilateral countermovement jump | Single inertial sensor unit | Antero-posterior axis angular excursion (°), medio-lateral axis angular excursion (°) and vertical axis angular excursion (°) of the trunk during initial absorption, propulsive and final absorption phases of the 50 cm vertical bilateral drop jump and 20 cm vertical unilateral drop jump tests; and during propulsive and final absorption phases of the vertical unilateral countermovement jump test | Greater angular excursions around the medio-lateral (X-axis) and antero-posterior (Y-axis) axis for controls while performing vertical unilateral countermovement jump |
|  |  |  |  |  |  |  |  |
| Shi et al. 2016 | ACLD | **ACLD group = 36**  M/F = 36/0  Range (Age) = 24-32 (28±3.7 years)  Body mass = 68.7±6.9 Kg  Height = 174±5 cm  BMI = NR | **Control group = 36**  M/F = 36/0  Range (Age) = 24-32 (27.8±3.7 years)  Body mass = 68.3±6.6 Kg  Height = 173±5 cm  BMI = NR | Walking, stair ascent and descent | 3D kinematics | Trunk flexion (anterior/posterior lean) (°), lateral trunk lean (°) and trunk rotation (anterior and posterior rotation) (°) during stance phase | Overall, different trunk strategies were performed by individuals with ACLD compared to controls during walking and stair negotiation |
|  |  |  |  |  |  |  |  |
| Sipprell et al. 2012 | ACLD | **ACLD group = 20**  M/F = 7/13  Range (Age) = NR (NR)  Body mass = NR  Height = NR  BMI = NR | **Control group = 20**  M/F = 7/13  Range (Age) = NR (NR)  Body mass = NR  Height = NR  BMI = NR | One-leg landing | 2D kinematics | Trunk flexion displacement (°) | Smaller trunk flexion displacement was found in those with ACLD compared to controls |
|  |  |  |  |  |  |  |  |
| Slater et al. 2020 | ACLR | **ACLR group (female) = 22**  Range (Age) = NR (20.1±2.4 years)  Body mass = 64.2±6.9 Kg  Height = 167±6.2 cm  BMI = NR  **ACLR group (male) = 11**  Range (Age) = NR (19.6±2.0 years)  Body mass = 76.4±13.1 Kg  Height = 176±9.6 cm  BMI = NR  **ACLR group = 33**  M/F = 11/22  Range (Age) = NR (NR)  Body mass = NR  Height = NR  BMI = NR | **Control group (female) = 18**  Range (Age) = NR (20.1±1.8 years)  Body mass = 66.1±8.4 Kg  Height = 168±6.5 cm  BMI = NR  **Control group (Men) = 11**  Range (Age) = NR (20.0±1.1 years)  Body mass = 76.2±9.2 Kg  Height = 179±7.5 cm  BMI = NR  **Control group = 29**  M/F = 11/18  Range (Age) = NR (NR)  Body mass = NR  Height = NR  BMI = NR | Running | 3D kinematics | Trunk flexion (°), lateral trunk lean (°) and trunk rotation (°), calculated for each % of the cycle (stance phase) before and after exercise (change score was calculated by subtracting pre-exercise from post-exercise) | Greater contralateral trunk lean in women who have undergone ACLR compared to injury-free women, from terminal stance through midswing phases, after exercise. Greater ipsilateral trunk rotation in women who have undergone ACLR compared to injury-free women, from midstance to midswing phases, after exercise. Greater contralateral trunk lean in men who have undergone ACLR compared to injury-free men at loading, midswing and late swing phases, after exercise |
|  |  |  |  |  |  |  |  |
| Smeets et al. 2019 | ACLR | **ACLR group = 21**  M/F = 15/6  Range (Age) = 16-40 (23.8±4.2 years)  Body mass = 76.4±11.3 Kg  Height = 178±9.4 cm  BMI = NR | **Control group = 21**  M/F = 15/6  Range (Age) = 16-40 (21.5±1.5 years)  Body mass = 72.5±9.3 Kg  Height = 179±9.4 cm  BMI = NR | Landing tasks (single leg hop for distance, medial and lateral hop, vertical hop with 90° of medial or lateral rotation) | 3D kinematics and kinetics | Trunk flexion (thorax flexion) (°) and trunk flexion moment (Nm) from initial contact until 500 milliseconds after initial contact of the landing phase of the hop tasks, relative to the pelvis coordinates system | No significant differences were found between groups in a pre-fatigue condition |
|  |  |  |  |  |  |  |  |
| Sparkes et al. 2019 | Knee OA  [KL 2-3] | **OA group = 10**  M/F = 2/8  Range (Age) = 45-65 (58.6±6 years)  Body mass = 91.5±15 Kg  Height = 171±8 cm  BMI = 30.8±3.1 Kg/m^2^ | **Control group = 8**  M/F = 2/6  Range (Age) = 45-65 (59.6±3.8 years)  Body mass = 87.5±16 Kg  Height = 170±12 cm  BMI = 29.8±3.3 Kg/m^2^ | Walking, stair ascent and descent | 3D kinematics | Trunk flexion (°), lateral trunk lean (°) and trunk rotation (°) during stance phase | Greater trunk flexion and trunk rotation were found in those with knee OA compared to controls while ascending stairs. Lower trunk rotation was found in those with knee OA compared to controls while descending stairs. No significant differences were found between groups during walking |
|  |  |  |  |  |  |  |  |
| Sritharan et al. 2020 | ACLR | **ACLR group = 65**  M/F = 41/24  Range (Age) = 18-50 (28.2±6.4 years)  Body mass = 78.6±14.7 Kg  Height = 176±10 cm  BMI = NR | **Control group = 32**  M/F = 18/17  Range (Age) = 18-50 (25.2±4.8 years)  Body mass = 68±11.4 Kg  Height = 170±10 cm  BMI = NR | Single leg forward hop task | 3D kinematics and kinetics | Trunk flexion (lumbar flexion) (º), trunk flexion torque (%BW*HT) (lumbar flexion torque) | Greater trunk flexion and trunk flexion torque were reported in those who have undergone ACLR compared to controls |
|  |  |  |  |  |  |  |  |
| Staab et al. 2014 | Knee OA  [KL 3-4] | **OA group = 12**  M/F = 10/2  Range (Age) = NR (44.4±7.6 years)  Body mass = 75.5±11.1 Kg  Height = 171±6.5 cm  BMI = 26.9±3.2 Kg/m^2^ | **Control group = 7**  M/F = 6/1  Range (Age) = NR (41.7±8.8 years)  Body mass = 73.7±9.8 Kg  Height = 175±7.2 cm  BMI = 26.1±2.9 Kg/m^2^ | Walking | 3D accelerometers | Lateral trunk lean (º) and angular velocity (º/s) | Greater lateral trunk lean in those with knee OA compared to controls. A more asymmetric lateral trunk lean acceleration in individuals with knee OA compared to controls was also reported |
|  |  |  |  |  |  |  |  |
| Tanaka et al. 2008 | Knee OA  [KL 2-4] | **OA group = 12**  M/F = 0/12  Range (Age) = NR (NR)  Body mass = NR  Height = NR  BMI = NR  **Unilateral OA group = 6**  M/F = 0/6  Range (Age) = NR (68.7±8.1 years)  Body mass = 56.8±8.4 Kg  Height = 157±7 cm  BMI = 22.9±2.7 Kg/m^2^  **Bilateral OA group = 6**  M/F = 0/6  Range (Age) = NR (72.3±3.9 years)  Body mass = 58.5±9.5 Kg  Height = 149±7 cm  BMI = 26.4±4.9 Kg/m^2^ | **Control group = 5**  M/F = 0/5  Range (Age) = NR (72±10.8 years)  Body mass = 46.9±7.3 Kg  Height = 144±4 cm  BMI = 22.4±2.4 Kg/m^2^ | Walking | 3D kinematics | Lateral trunk lean (°) during stance phase | No significant differences were found between groups for lateral trunk lean |
|  |  |  |  |  |  |  |  |
| Trigsted et al. 2017 | ACLR | **ACLR group = 33**  M/F = 0/33  Range (Age) = 18-25 (18.8±1.7 years)  Body mass = 66.7±8.9 Kg  Height = 167.8±6.6 cm  BMI = NR | **Control group = 31**  M/F = 0/31  Range (Age) = 18-25 (19.2±1 years)  Body mass = 60.9±6.3 Kg  Height = 166±5.4 cm  BMI = NR | Single hop for distance | Electromagnetic tracking system | Trunk flexion (°) and lateral trunk lean (°) during loading phase of landing (defined as initial contact to peak knee flexion) | No significant differences were found between groups for trunk kinematics in the sagittal and frontal planes |
|  |  |  |  |  |  |  |  |
| Turcot et al. 2012 | Knee OA  [KL 4] | **OA group = 25**  M/F = 14/11  Range (Age) = NR (69±5 years)  Body mass = 83.3±15.4 Kg  Height = 169±8 cm  BMI = 29.1±4.8 Kg/m^2^ | **Control group = 20**  M/F = 10/10  Range (Age) = NR (67±7 years)  Body mass = 67.4±10.6 Kg  Height = 170±10 cm  BMI = 23.2±2.4 Kg/m^2^ | Sit-to-stand | 3D kinematics | Trunk flexion (°) and lateral trunk lean (°) | Greater trunk flexion and contralateral trunk lean were found in those with knee OA compared to controls |
|  |  |  |  |  |  |  |  |
| Turcot et al. 2013 | Knee OA  [KL 4] | **OA group = 60**  M/F = 27/33  Range (Age) = NR (NR)  Body mass = NR  Height = NR  BMI = NR  **Varus group = 46**  M/F = 24/22  Range (Age) = NR (68±6 years)  Body mass = NR  Height = NR  BMI = 30.7±5.3 Kg/m^2^  **Varus group = 14**  M/F = 3/11  Range (Age) = NR (69±8 years)  Body mass = NR  Height = NR  BMI = 29.3±4.3 Kg/m^2^ | **Control group = 26**  M/F = 11/15  Range (Age) = NR (66±8 years)  Body mass = NR  Height = NR  BMI = 23.5±2.4 Kg/m^2^ | Walking | 3D kinematics | Trunk flexion (°), lateral trunk lean (obliquity) (°), trunk rotation (°) during stance phase | Greater trunk flexion and ipsilateral trunk lean in those with knee OA and knee varus compared to controls |
|  |  |  |  |  |  |  |  |
| Turcot et al. 2015 | Knee OA  [KL 4] | **OA group = 87**  M/F = 41/46  Range (Age) = NR (69±7 years)  Body mass = NR  Height = NR  BMI = 31.4±5.7 Kg/m^2^ | **Control group = 25**  M/F = 12/13  Range (Age) = NR (68±6 years)  Body mass = NR  Height = NR  BMI = 24.8±3.6 Kg/m^2^ | Quiet standing task | 3D kinematics | Trunk flexion (°) and lateral trunk lean (°) | Greater trunk flexion was found in those with knee OA compared to controls |
|  |  |  |  |  |  |  |  |
| Van der Straaten et al. 2020a | Knee OA  [KL 3-4] | **OA group = 19**  M/F = 12/7  Range (Age) = 50-75 (65.1±5.2 years)  Body mass = 79.8±8.4 Kg  Height = 175±8 cm  BMI = 26±2.2 Kg/m^2^ | **Control group = 12**  M/F = 6/6  Range (Age) = 50-75 (59.8±7 years)  Body mass = 74.3±14.9 Kg  Height = 171±10 cm  BMI = 25.1±3.4 Kg/m^2^ | Unipodal stance task | Inertial sensor system  3D kinematics | Trunk flexion (°), lateral trunk lean (°), and trunk rotation (°) | Greater contralateral trunk lean was found in those with knee OA compared to controls based on inertial sensors and three-dimensional system analyses. Greater trunk extension was found in those with knee OA compared to controls based on camera-based three-dimensional analysis system; whereas greater trunk extension was found for controls compared to individuals with knee OA based on inertial sensors analysis. Greater ipsilateral trunk rotation was found in those with knee OA compared to controls based on three-dimensional system analysis; whereas no differences between groups were found based on inertial sensors analysis |
|  |  |  |  |  |  |  |  |
| Van der Straaten et al. 2020b | Knee OA  [KL 3-4] | **OA group = 19**  M/F = 12/7  Range (Age) = 50-75 (65.1±5.2 years)  Body mass = 79.8±8.4 Kg  Height = 175±8 cm  BMI = 26±2.2 Kg/m^2^ | **Control group = 12**  M/F = 6/6  Range (Age) = 50-75 (59.8±7 years)  Body mass = 74.3±14.9 Kg  Height = 171±10 cm  BMI = 25.1±3.4 Kg/m^2^ | Walking, forward lunge, sideward lunge, stair ascent, stair descent, single leg squat and sit-to-stand tasks | Inertial sensor system  3D kinematics | Trunk flexion (°), lateral trunk lean (°), and trunk rotation (°) | Only lower trunk rotation was reported to be found in individuals with knee OA based on camera-based and inertial sensor three-dimensional analysis systems during stance and swing phase of walking |
|  |  |  |  |  |  |  |  |
| Waiteman et al. 2021 | PFP | **PFP_Crepitus_ group = 29**  M/F = 0/29  Range (Age) = 18-35 (22.8±2.8 years)  Body mass = 65±8.6 Kg  Height = 161±5 cm  BMI = 24.9±3.4 Kg/m^2^  Whatling**PFP group = 57**  M/F = 0/57  Range (Age) = 18-35 (NR years)  Body mass = NR  Height = NR  BMI = NR | **Control_Crepitus_ group = 17**  M/F = 0/17  Range (Age) = 18-35 (22.1±3.5 years)  Body mass = 55.7±7.1 Kg  Height = 160±7 cm  BMI = 21.6±2.1 Kg/m^2^  **Control_NOCrepitus_ group = 29**  M/F = 0/29  Range (Age) = 18-35 (22.3±2.9 years)  Body mass = 57.1±6.2 Kg  Height = 161±5 cm  BMI = 22±2.9 Kg/m^2^  **Control group = 46**  M/F = 0/46  Range (Age) = 18-35 (NR years)  Body mass = NR  Height = NR  BMI = NR | Stair ascent | 3D kinematics | Trunk flexion (°) | No significant differences were found between groups for trunk kinematics in the sagittal plane |
|  |  |  |  |  |  |  |  |
| Whatling et al. 2020 | Knee OA  [KL 2-4] | **OA group = 18**  M/F = 16/2  Range (Age) = 18-80 (51.2±7 years)  Body mass = 90.1±22.5 Kg  Height = 170±10 cm  BMI = 29.5±5.8 Kg/m^2^ | **Control group = 18**  M/F = 10/8  Range (Age) = 18-80 (34.6±11.2 years)  Body mass = 70.2±12.9 Kg  Height = 170±10 cm  BMI = 24.5±4.1 Kg/m^2^ | Walking | 3D kinematics | Lateral trunk lean (trunk sway range of motion) (°) | Greater ipsilateral trunk lean was found in those with knee OA before high tibial osteotomy, but not after the surgery procedure, compared to controls |

^+^ Groups divided as less severe (*Kellgren-Lawrence* grade 1 and 2) and more severe (*Kellgren-Lawrence* grade 3 and 4).

^++^ Groups divided as Uni-pain/Uni-xray group (individuals with unilateral pain and structural osteoarthritis), Uni-pain/Bi-xray group (individuals with unilateral pain but bilateral structural osteoarthritis), and Bi-pain/Bi-xray group (individuals with bilateral pain and structural osteoarthritis).

^†^ Groups divided as mild (*Kellgren-Lawrence* grade 2), moderate (*Kellgren-Lawrence* grade 3), and severe (*Kellgren-Lawrence* grade 4).

^††^ Groups divided as mild (*Kellgren-Lawrence* grade 2) and moderate (*Kellgren-Lawrence* grade 3).

**^‡^** Data expressed as median (interquartile interval) or range.

*ACLD* anterior cruciate ligament deficiency, *ACLR* anterior cruciate ligament reconstruction, *BMI* body mass index, *M/F* male/female, *NR* not reported, *PFP* patellofemoral pain, *OA* osteoarthritis, *PT* patellar tendinopathy, *TKA* total knee arthroplasty, *KL* *Kellgren-Lawrence* grade.

**Additional file 1D** Methodological quality assessment of using the Downs and Black checklist

| **Study** | **Aim**  **(1)** | **Outcomes**  **(2)** | **Participants**  **(3)** | **Confounders**  **(5)** | **Findings**  **(6)** | **Random variability**  **(7)** | **Probability**  **(10)** | **External validity**  **(11)** | **External validity**  **(12)** | **Blinding**  **(15)** | **Statistical test**  **(18)** | **Accurate outcomes**  **(20)** | **Confounding adjustment**  **(25)** | **Power**  **(27)** | **Score** | **Quality** |
| --- | --- | --- | --- | --- | --- | --- | --- | --- | --- | --- | --- | --- | --- | --- | --- | --- |
| **Knee osteoarthritis** | | | | | | | | | | | | | | | |  |
| Asay et al. 2009 | Yes | Yes | Yes | No/UTD | Yes | Yes | Yes | No/UTD | No/UTD | No/UTD | Yes | No/UTD | No/UTD | No/UTD | 7 | MQ |
| Azma et al. 2015 | No/UTD | No/UTD | No/UTD | No/UTD | Yes | Yes | Yes | No/UTD | No/UTD | No/UTD | Yes | No/UTD | No/UTD | No/UTD | 4 | LQ |
| Bechard et al. 2012 | Yes | Yes | Yes | Yes/partially | Yes | Yes | Yes | No/UTD | No/UTD | No/UTD | Yes | Yes | No/UTD | Yes | 10 | MQ |
| Boekesteijn et al. 2021 | Yes | No/UTD | Yes | Yes/partially | Yes | Yes | Yes | No/UTD | No/UTD | No/UTD | Yes | Yes | No/UTD | No/UTD | 8 | MQ |
| Creaby et al. 2012 | Yes | Yes | Yes | Yes/partially | Yes | Yes | Yes | No/UTD | No/UTD | No/UTD | Yes | Yes | Yes | No/UTD | 10 | MQ |
| Fu et al. 2021 | Yes | Yes | Yes | Yes | Yes | Yes | Yes | No/UTD | No/UTD | No/UTD | Yes | Yes | No/UTD | Yes | 11 | HQ |
| Hálfdanardóttir et al. 2018 | Yes | Yes | Yes | Yes/partially | Yes | Yes | Yes | No/UTD | No/UTD | No/UTD | Yes | Yes | Yes | No/UTD | 10 | MQ |
| Hart et al. 2015 | Yes | No/UTD | Yes | Yes/partially | Yes | Yes | Yes | No/UTD | No/UTD | No/UTD | Yes | Yes | Yes | Yes | 10 | MQ |
| Hunt et al. 2010 | Yes | Yes | Yes | Yes/partially | Yes | Yes | Yes | No/UTD | No/UTD | No/UTD | Yes | Yes | No/UTD | Yes | 10 | MQ |
| Kean et al. 2013 | Yes | Yes | Yes | Yes/partially | Yes | Yes | Yes | No/UTD | No/UTD | No/UTD | Yes | Yes | No/UTD | No/UTD | 9 | MQ |
| Kuwahara et al. 2019^††^ | Yes | Yes | Yes | Yes/partially | Yes | Yes | Yes | No/UTD | No/UTD | No/UTD | Yes | Yes | No/UTD | No/UTD | 9 | MQ |
| Linley et al. 2010 | Yes | Yes | Yes | Yes/partially | Yes | Yes | Yes | No/UTD | No/UTD | No/UTD | Yes | Yes | No/UTD | No/UTD | 9 | MQ |
| Magalhães et al. 2013 | Yes | Yes | Yes | Yes/partially | Yes | Yes | Yes | No/UTD | No/UTD | No/UTD | Yes | Yes | No/UTD | No/UTD | 9 | MQ |
| Meireles et al. 2019 | Yes | No/UTD | Yes | Yes/partially | Yes | Yes | Yes | No/UTD | No/UTD | No/UTD | Yes | Yes | Yes | No/UTD | 9 | MQ |
| Petrella et al. 2019 | Yes | Yes | Yes | Yes/partially | Yes | Yes | Yes | No/UTD | No/UTD | No/UTD | Yes | Yes | No/UTD | Yes | 10 | MQ |
| Preece et al. 2019 | Yes | Yes | No/UTD | Yes/partially | Yes | Yes | Yes | No/UTD | No/UTD | No/UTD | Yes | Yes | No/UTD | No/UTD | 8 | MQ |
| Preece and Alghamdi 2021 | Yes | Yes | Yes | Yes/partially | Yes | Yes | Yes | No/UTD | No/UTD | No/UTD | Yes | Yes | No/UTD | No/UTD | 9 | MQ |
| Sagawa et al. 2017 | Yes | No/UTD | No/UTD | Yes/partially | Yes | Yes | Yes | No/UTD | No/UTD | No/UTD | Yes | Yes | No/UTD | No/UTD | 7 | MQ |
| Sparkes et al. 2019 | Yes | Yes | Yes | Yes | Yes | Yes | Yes | No/UTD | No/UTD | No/UTD | Yes | Yes | Yes | No/UTD | 11 | HQ |
| Staab et al. 2014 | Yes | No/UTD | No/UTD | Yes/partially | Yes | Yes | No/UTD | No/UTD | No/UTD | No/UTD | Yes | Yes | No/UTD | No/UTD | 6 | MQ |
| Tanaka et al. 2008 | Yes | Yes | No/UTD | Yes/partially | Yes | Yes | No/UTD | No/UTD | No/UTD | No/UTD | Yes | Yes | No/UTD | No/UTD | 7 | MQ |
| Turcot et al. 2012 | Yes | Yes | Yes | Yes/partially | Yes | Yes | Yes | No/UTD | No/UTD | No/UTD | Yes | Yes | No/UTD | No/UTD | 9 | MQ |
| Turcot et al. 2013 | Yes | No/UTD | Yes | Yes/partially | Yes | Yes | Yes | No/UTD | No/UTD | No/UTD | Yes | Yes | No/UTD | No/UTD | 8 | MQ |
| Turcot et al. 2015 | Yes | Yes | Yes | Yes/partially | Yes | Yes | Yes | No/UTD | No/UTD | No/UTD | Yes | Yes | No/UTD | No/UTD | 9 | MQ |
| Van der Straaten et al. 2020a | Yes | Yes | Yes | Yes/partially | Yes | Yes | Yes | No/UTD | No/UTD | No/UTD | Yes | Yes | No/UTD | Yes | 10 | MQ |
| Van der Straaten et al. 2020b | Yes | Yes | Yes | Yes/partially | Yes | Yes | Yes | No/UTD | No/UTD | No/UTD | Yes | Yes | No/UTD | Yes | 10 | MQ |
| Whatling et al. 2020 | Yes | No/UTD | Yes | Yes/partially | Yes | Yes | Yes | No/UTD | No/UTD | No/UTD | Yes | Yes | No/UTD | No/UTD | 8 | MQ |
| **Total Knee Arthroplasty** | | | | | | | | | | | | | | | | |
| Bjerke et al. 2014 | No/UTD | Yes | Yes | Yes | Yes | Yes | Yes | No/UTD | No/UTD | No/UTD | Yes | Yes | No/UTD | No/UTD | 9 | MQ |
| Boonstra et al. 2008 | Yes | Yes | Yes | Yes | Yes | Yes | Yes | No/UTD | No/UTD | No/UTD | Yes | Yes | Yes | No/UTD | 11 | HQ |
| Fenner et al. 2014 | Yes | No/UTD | Yes | Yes/partially | Yes | Yes | Yes | No/UTD | No/UTD | No/UTD | Yes | Yes | No/UTD | No/UTD | 8 | MQ |
| Fenner et al. 2017 | Yes | No/UTD | Yes | Yes/partially | Yes | Yes | Yes | No/UTD | No/UTD | No/UTD | Yes | Yes | No/UTD | No/UTD | 8 | MQ |
| Kuwahara et al. 2019^††^ | Yes | Yes | Yes | Yes/partially | Yes | Yes | Yes | No/UTD | No/UTD | No/UTD | Yes | Yes | No/UTD | No/UTD | 9 | MQ |
| Li et al. 2013 | Yes | No/UTD | No/UTD | Yes | Yes | Yes | Yes | No/UTD | No/UTD | No/UTD | Yes | Yes | Yes | No/UTD | 9 | MQ |
| Pozzi et al. 2016 | Yes | Yes | Yes | Yes/partially | Yes | Yes | Yes | No/UTD | No/UTD | No/UTD | Yes | Yes | Yes | No/UTD | 10 | MQ |
| Saari et al. 2004 | No/UTD | Yes | No/UTD | Yes/partially | Yes | Yes | No/UTD | No/UTD | No/UTD | No/UTD | Yes | Yes | No/UTD | No/UTD | 6 | MQ |
| **Patellofemoral pain** | | | | | | | | | | | | | | | |  |
| Alvim et al. 2019 | Yes | No/UTD | No/UTD | Yes | Yes | No/UTD | No/UTD | No/UTD | No/UTD | No/UTD | Yes | Yes | Yes | No/UTD | 7 | MQ |
| Baellow et al. 2020 | Yes | Yes | Yes | Yes/partially | Yes | Yes | Yes | No/UTD | No/UTD | No/UTD | Yes | Yes | No/UTD | No/UTD | 9 | MQ |
| Bazett-Jones et al. 2013 | Yes | Yes | Yes | Yes/partially | Yes | Yes | Yes | No/UTD | No/UTD | No/UTD | Yes | Yes | Yes | No/UTD | 10 | MQ |
| Bley et al. 2014 | Yes | Yes | Yes | Yes | Yes | Yes | Yes | No/UTD | No/UTD | No/UTD | Yes | Yes | Yes | Yes | 12 | HQ |
| Bramah et al. 2018 | Yes | Yes | Yes | Yes | Yes | Yes | Yes | No/UTD | No/UTD | No/UTD | Yes | Yes | Yes | Yes | 12 | HQ |
| Claudon et al. 2012 | Yes | Yes | Yes | Yes | Yes | Yes | Yes | No/UTD | No/UTD | No/UTD | Yes | Yes | Yes | No/UTD | 11 | HQ |
| Dos Reis et al. 2015 | Yes | No/UTD | Yes | Yes | Yes | Yes | Yes | No/UTD | No/UTD | No/UTD | Yes | Yes | Yes | Yes | 11 | HQ |
| Glaviano et al. 2019 | Yes | Yes | Yes | Yes | Yes | Yes | Yes | No/UTD | No/UTD | No/UTD | Yes | Yes | No/UTD | No/UTD | 10 | MQ |
| Haghighat et al. 2021 | Yes | Yes | Yes | Yes | Yes | Yes | Yes | No/UTD | No/UTD | No/UTD | Yes | Yes | Yes | Yes | 12 | HQ |
| Hébert et al. 1994 | No/UTD | No/UTD | No/UTD | Yes/partially | Yes | No/UTD | No/UTD | No/UTD | No/UTD | No/UTD | Yes | Yes | No/UTD | No/UTD | 4 | LQ |
| Ho et al. 2021 | Yes | Yes | Yes | Yes | Yes | Yes | Yes | No/UTD | No/UTD | No/UTD | Yes | Yes | Yes | Yes | 12 | HQ |
| Nakagawa et al. 2012a | Yes | Yes | Yes | Yes | Yes | Yes | Yes | No/UTD | No/UTD | No/UTD | Yes | Yes | Yes | Yes | 12 | HQ |
| Nakagawa et al. 2012b | Yes | Yes | Yes | Yes/partially | Yes | Yes | Yes | No/UTD | No/UTD | No/UTD | Yes | Yes | Yes | Yes | 11 | HQ |
| Nakagawa et al. 2015 | Yes | Yes | No/UTD | Yes/partially | Yes | Yes | Yes | No/UTD | No/UTD | No/UTD | Yes | Yes | No/UTD | No/UTD | 8 | MQ |
| Noehren et al. 2012 | Yes | Yes | No/UTD | Yes | Yes | Yes | Yes | No/UTD | No/UTD | No/UTD | Yes | Yes | Yes | No/UTD | 10 | MQ |
| Novello et al. 2018 | Yes | No/UTD | Yes | Yes | Yes | Yes | Yes | No/UTD | No/UTD | No/UTD | Yes | Yes | Yes | No/UTD | 10 | MQ |
| Nunes et al. 2020 | Yes | Yes | Yes | Yes | Yes | Yes | Yes | No/UTD | No/UTD | Yes | Yes | Yes | No/UTD | Yes | 12 | HQ |
| Schwane et al. 2015 | Yes | Yes | Yes | Yes | Yes | Yes | Yes | No/UTD | No/UTD | No/UTD | Yes | Yes | Yes | Yes | 12 | HQ |
| Waiteman et al. 2021 | Yes | Yes | Yes | Yes | Yes | Yes | Yes | No/UTD | No/UTD | No/UTD | Yes | Yes | Yes | Yes | 12 | HQ |
| **Patellofemoral Tendinopathy** | | | | | | | | | | | | | | | | |
| Barker-Davies et al. 2019 | Yes | Yes | Yes | Yes/partially | Yes | Yes | Yes | No/UTD | No/UTD | No/UTD | Yes | Yes | Yes | Yes | 11 | HQ |
| Scattone et al. 2017 | Yes | Yes | Yes | Yes | Yes | Yes | Yes | No/UTD | No/UTD | No/UTD | Yes | Yes | Yes | Yes | 12 | HQ |
| **Anterior cruciate ligament deficiency** | | | | | | | | | | | | | | | |  |
| Davies et al. 2018^†^ | Yes | Yes | No/UTD | Yes/partially | Yes | Yes | Yes | No/UTD | No/UTD | No/UTD | Yes | Yes | No/UTD | No/UTD | 8 | MQ |
| Fukuda et al. 2021a | Yes | Yes | Yes | Yes | No/UTD | Yes | No/UTD | No/UTD | No/UTD | No/UTD | Yes | Yes | No/UTD | Yes | 9 | MQ |
| Hewett et al. 2009 | Yes | Yes | No/UTD | No/UTD | Yes | Yes | Yes | No/UTD | No/UTD | No/UTD | Yes | Yes | No/UTD | No/UTD | 7 | MQ |
| Markstrom et al. 2018^†^ | Yes | Yes | Yes | Yes/partially | Yes | Yes | Yes | No/UTD | No/UTD | No/UTD | Yes | Yes | No/UTD | Yes | 10 | MQ |
| Oberlander et al. 2012 | Yes | Yes | No/UTD | Yes | Yes | Yes | No/UTD | No/UTD | No/UTD | No/UTD | Yes | Yes | Yes | No/UTD | 9 | MQ |
| Shi et al. 2016 | No/UTD | Yes | Yes | Yes/partially | Yes | Yes | Yes | No/UTD | No/UTD | No/UTD | Yes | Yes | No/UTD | No/UTD | 8 | MQ |
| Sipprell et al. 2012 | Yes | Yes | No/UTD | No/UTD | Yes | Yes | Yes | No/UTD | No/UTD | Yes | Yes | Yes | No/UTD | Yes | 9 | MQ |
| **Anterior cruciate ligament reconstruction** | | | | | | | | | | | | | | | |  |
| Armitano et al. 2017 | Yes | No/UTD | Yes | Yes | Yes | Yes | Yes | No/UTD | No/UTD | No/UTD | Yes | Yes | Yes | No/UTD | 10 | MQ |
| Bell et al. 2014 | Yes | No/UTD | Yes | Yes/partially | Yes | Yes | Yes | No/UTD | No/UTD | No/UTD | Yes | Yes | No/UTD | Yes | 9 | MQ |
| Boggess et al. 2018 | Yes | Yes | Yes | Yes | Yes | Yes | Yes | No/UTD | No/UTD | No/UTD | Yes | Yes | Yes | No/UTD | 11 | MQ |
| Davies et al. 2018^†^ | Yes | Yes | No/UTD | Yes/partially | Yes | Yes | Yes | No/UTD | No/UTD | No/UTD | Yes | Yes | No/UTD | No/UTD | 8 | MQ |
| Fryer et al. 2019 | Yes | Yes | Yes | Yes | Yes | Yes | Yes | No/UTD | No/UTD | No/UTD | Yes | Yes | Yes | No/UTD | 11 | HQ |
| Fukuda et al. 2021b | Yes | Yes | Yes | Yes | Yes | Yes | Yes | No/UTD | No/UTD | No/UTD | Yes | Yes | No/UTD | Yes | 11 | HQ |
| Kotsifaki et al. 2021a | Yes | Yes | Yes | Yes | Yes | Yes | Yes | No/UTD | No/UTD | No/UTD | Yes | Yes | No/UTD | Yes | 11 | HQ |
| Kotsifaki et al. 2021b | Yes | Yes | Yes | Yes/partially | Yes | Yes | Yes | No/UTD | No/UTD | No/UTD | Yes | Yes | No/UTD | No/UTD | 9 | MQ |
| Kotsifaki et al. 2022 | Yes | Yes | Yes | Yes/partially | Yes | Yes | Yes | No/UTD | No/UTD | No/UTD | Yes | Yes | No/UTD | No/UTD | 9 | MQ |
| Lessi and Serrão 2017 | Yes | Yes | Yes | Yes | Yes | Yes | Yes | No/UTD | No/UTD | No/UTD | Yes | Yes | Yes | Yes | 12 | HQ |
| Markstrom et al. 2018^†^ | Yes | Yes | Yes | Yes/partially | Yes | Yes | Yes | No/UTD | No/UTD | No/UTD | Yes | Yes | No/UTD | Yes | 10 | MQ |
| Markstrom et al. 2020 | Yes | Yes | Yes | Yes | Yes | Yes | Yes | No/UTD | No/UTD | No/UTD | Yes | Yes | Yes | No/UTD | 11 | HQ |
| Markstrom et al. 2021 | Yes | Yes | Yes | Yes/partially | Yes | Yes | Yes | No/UTD | No/UTD | No/UTD | Yes | Yes | Yes | No/UTD | 10 | MQ |
| Noehren et al. 2014 | Yes | Yes | No/UTD | Yes | Yes | Yes | Yes | No/UTD | No/UTD | No/UTD | Yes | Yes | Yes | No/UTD | 10 | MQ |
| Setuain et al. 2015a | Yes | No/UTD | No/UTD | Yes/partially | No/UTD | Yes | No/UTD | No/UTD | No/UTD | No/UTD | Yes | Yes | No/UTD | No/UTD | 5 | LQ |
| Scarneo-Miller et al. 2019 | Yes | Yes | No/UTD | Yes | Yes | Yes | Yes | No/UTD | No/UTD | No/UTD | Yes | Yes | Yes | Yes | 11 | HQ |
| Setuain et al. 2015b | Yes | No/UTD | Yes | Yes | Yes | Yes | Yes | No/UTD | No/UTD | No/UTD | Yes | Yes | No/UTD | No/UTD | 9 | MQ |
| Slater et al. 2020 | Yes | Yes | No/UTD | Yes | Yes | Yes | Yes | No/UTD | No/UTD | No/UTD | Yes | Yes | No/UTD | No/UTD | 9 | MQ |
| Smeets et al. 2019 | Yes | No/UTD | No/UTD | Yes/partially | Yes | Yes | Yes | No/UTD | No/UTD | No/UTD | Yes | Yes | No/UTD | No/UTD | 7 | MQ |
| Sritharan et al. 2020 | Yes | No/UTD | Yes | Yes | Yes | Yes | Yes | No/UTD | No/UTD | No/UTD | Yes | Yes | No/UTD | No/UTD | 9 | MQ |
| Trigsted et al. 2017 | Yes | Yes | No/UTD | Yes/partially | Yes | Yes | Yes | No/UTD | No/UTD | No/UTD | Yes | Yes | No/UTD | Yes | 9 | MQ |

^†^ Study included individuals with anterior cruciate ligament deficiency and reconstruction.

^††^ Study included individuals with tibiofemoral osteoarthritis and total knee arthroplasty.

*HQ* high quality, *LQ* low quality, *MQ* moderate quality, *UTD* unable to determine.

**Additional file 1E** Qualitative synthesis of unpooled data

**Knee OA versus controls**

Trunk kinematics in the sagittal plane

Two moderate quality studies [1,2] reported greater trunk flexion in individuals with knee OA during walking, while 1 high [3] and 2 moderate [4,5] quality studies reported no differences between groups. One moderate quality study [4] reported no differences between groups for trunk kinematics in the sagittal plane during squatting tasks. One moderate quality study [6] reported greater trunk flexion in individuals with knee OA during stair ascent and descent, while another moderate quality study [4] reported no differences between groups. One moderate quality study [7] reported greater trunk angle in the sagittal plane in individuals with knee OA during quite standing, while 1 moderate quality study [8] reported conflicting findings during a unipodal stance task (i.e., greater trunk extension in individuals with knee OA using a camera-based system, but lower trunk extension using an inertial sensor system).

Trunk kinematics in the frontal plane

During walking, 2 moderate quality studies [5,9] reported greater ipsilateral trunk lean in individuals with knee OA, 1 moderate quality study [10] reported lower ipsilateral trunk lean in individuals with knee OA, and 7 studies (1 high [3], 5 moderate [1,4,10–12], 1 low quality [13]) reported no differences between groups. One moderate quality study [4] reported no differences between groups for trunk kinematics in the frontal plane during squatting tasks. One high quality study [3] reported lower lateral trunk lean (side not specified) in individuals with knee OA during stair descent, but not during stair ascent; two other moderate quality studies [4,6] reported no differences between groups during stair ascent and descent. One moderate quality study [7] reported no differences between groups for lateral trunk lean during a quiet standing task. One moderate quality study [8] reported greater contralateral trunk lean in individuals with knee OA during a unipodal stance task.

Trunk kinematics in the transverse plane

One moderate quality study [4] reported lower trunk rotation (side not specified) in individuals with knee OA during walking, while 2 studies (1 high [3], 1 moderate [5] quality) reported no differences between groups. One moderate quality study [4] reported no difference between groups for trunk kinematics in the transverse plane during squatting tasks. During stair negotiation, 1 high quality study [3] reported greater trunk rotation (side not specified) in individuals with knee OA during stair ascent but lower trunk rotation (side not specified) during stair descent; two moderate quality studies [4,6] reported no differences between groups. One moderate quality study [8] reported greater trunk rotation in individuals with knee OA during a unipodal stance task using a camera-based system, but no differences between groups using an inertial sensor system.

**TKA versus controls**

Trunk kinematics in the sagittal plane

One moderate quality study [14] reported no differences between groups for trunk flexion during walking. Two studies (1 high [15], 1 moderate [16] quality) reported no differences between groups for trunk flexion during sit-to-stand. Two moderate quality studies [14,17] reported no differences between groups for trunk flexion during stepping tasks.

Trunk kinematics in the frontal plane

One moderate quality study [14] reported no differences between groups for ipsilateral trunk lean during walking and contralateral trunk lean during stair ascent.

Trunk kinematics in the transverse plane

One moderate quality study [14] reported no differences between groups for contralateral trunk rotation during walking and stair ascent.

**PFP versus controls**

Trunk kinematics in the sagittal plane

One high quality study [18] reported greater trunk flexion in individuals with PFP during walking, while two high quality studies [19,20] reported no differences between groups during running. Three studies (1 high [21], 1 moderate [22], 1 low quality [23]) reported no differences between groups for trunk flexion during squatting tasks [23–25] and landing from a single leg drop vertical jump [21]. One high [26] and 1 moderate [27] quality studies reported greater trunk flexion in individuals with PFP during take-off phase of a single leg triple hop task.

Trunk kinematics in the frontal plane

Two studies (1 high [19] and 1 moderate [28] quality) reported no differences between groups for lateral trunk lean during running. One high quality study [21] reported no differences between groups for ipsilateral trunk lean during single leg squat. One moderate quality study [29] reported no differences between groups for contralateral trunk lean during stair descent. One high quality study [21] reported greater ipsilateral trunk lean in individuals with PFP during landing from a single leg drop vertical jump. One high quality study [26] reported greater ipsilateral trunk lean in individuals with PFP during take-off phase of a single leg triple hop task, while one moderate quality study [27] reported no differences between groups in the same task.

Trunk kinematics in the transverse plane

One high quality study [24] reported no differences between groups for ipsilateral and contralateral trunk rotation during stair descent. One moderate quality study [27] reported no differences between groups for trunk kinematics in the transverse plane during take-off phase of a single leg triple hop task.

**ACLR versus controls**

Trunk kinematics in the sagittal plane

One moderate quality study [30] reported greater trunk flexion in individuals with ACLR during running, while two studies (1 high [31] and 1 moderate [32] quality) reported no differences between groups. During single leg squat, one moderate quality study [33] reported lower trunk flexion in individuals with ACLR. One moderate quality study [34] reported greater trunk flexion in individuals with ipsilateral semitendinosus and gracilis autograft during single leg squat, while no differences between groups were reported for those with ipsilateral bone–patellar tendon–bone autograft. Two moderate quality studies [35,36] reported greater trunk flexion in individuals with ACLR during landing and jumping of single leg drop jump [36] and during first and second rebounds (landing and jumping) of single leg triple hop for distance [35]; while 2 moderate quality studies [33,37] reported no differences between groups during different landing tasks.

Trunk kinematics in the frontal plane

One moderate quality study [30] reported greater ipsilateral trunk lean in individuals with ACLR during running, while one moderate quality study [32] reported greater contralateral trunk lean. One high [31] and 1 moderate [38] quality studies reported no differences between groups for lateral trunk lean during walking and running. During single leg squat, one moderate quality study [34] reported greater ipsilateral trunk lean in individuals with ipsilateral semitendinosus and gracilis autograft, while no differences between groups were reported for those with ipsilateral bone–patellar tendon–bone autograft. Two moderate quality studies [33,38] reported no differences between groups for contralateral trunk lean during single leg squat. Two moderate quality studies [33,38] reported no differences between groups for contralateral trunk lean during landing from a single leg hop for distance [38] and a single leg jump and cut [33].

Trunk kinematics in the transverse plane

One moderate quality study [32] reported greater ipsilateral trunk rotation in individuals with ACLR during running. One moderate quality study [33] reported no differences between groups for trunk rotation (side not specified) during single leg squat. One high quality study [39] reported lower trunk rotation (side not specified) in individuals with ACLR during landing of single leg hop for distance, while one moderate quality study [33] reported no differences between groups during landing from a single leg jump and cut.

Trunk kinetics

One moderate quality study [40] reported greater trunk flexion moment in individuals with ACLR during landing of single leg hop for distance, while another moderate quality study [37] reported no differences between groups during different landing tasks.

**References**

1. Preece SJ, Algarni AS, Jones RK. Trunk flexion during walking in people with knee osteoarthritis. Gait Posture. 2019;72:202–5.

2. Preece SJ, Alghamdi W. Increased trunk flexion may underlie elevated knee flexor activity in people with knee osteoarthritis. Knee. 2021;33:216–25.

3. Sparkes V, Whatling GM, Biggs P, Khatib N, Al-Amri M, Williams D, et al. Comparison of gait, functional activities, and patient-reported outcome measures in patients with knee osteoarthritis and healthy adults using 3D motion analysis and activity monitoring: An exploratory case-control analysis. Orthop Res Rev. 2019;11:129–40.

4. Van Der Straaten R, Wesseling M, Jonkers I, Vanwanseele B, Bruijnes AKBD, Malcorps J, et al. Functional movement assessment by means of inertial sensor technology to discriminate between movement behaviour of healthy controls and persons with knee osteoarthritis. J Neuroeng Rehabil. 2020;17:65.

5. Turcot K, Armand S, Lübbeke A, Fritschy D, Hoffmeyer P, Suvà D. Does knee alignment influence gait in patients with severe knee osteoarthritis? Clin Biomech. 2013;28:34–9.

6. Meireles S, Reeves ND, Jones RK, Smith CR, Thelen DG, Jonkers I. Patients with medial knee osteoarthritis reduce medial knee contact forces by altering trunk kinematics, progression speed, and stepping strategy during stair ascent and descent: A pilot study. J Appl Biomech. 2019;35:280–9.

7. Turcot K, Sagawa Y, Hoffmeyer P, Suvà D, Armand S. Multi-joint postural behavior in patients with knee osteoarthritis. Knee. 2015;22:517–21.

8. van der Straaten R, Wesseling M, Jonkers I, Vanwanseele B, Bruijnes AKBD, Malcorps J, et al. Discriminant validity of 3D joint kinematics and centre of mass displacement measured by inertial sensor technology during the unipodal stance task. PLoS One. 2020;15:e0232513.

9. Magalhães CMB, Resende RA, Kirkwood RN. Increased hip internal abduction moment and reduced speed are the gait strategies used by women with knee osteoarthritis. J Electromyogr Kinesiol. 2013;23:1243–9.

10. Hálfdanardóttir F, Ramsey DK, Briem K. Timing of frontal plane trunk lean, not magnitude, mediates frontal plane knee joint loading in patients with moderate medial knee osteoarthritis. Adv Orthop. 2018;20:2018:4526872.

11. Kean CO, Bennell KL, Wrigley T V., Hinman RS. Modified walking shoes for knee osteoarthritis: Mechanisms for reductions in the knee adduction moment. J Biomech. 2013;46:2060–6.

12. Linley HS, Sled EA, Culham EG, Deluzio KJ. A biomechanical analysis of trunk and pelvis motion during gait in subjects with knee osteoarthritis compared to control subjects. Clin Biomech. 2010;25:1003–10.

13. Azma K, Mahdavi E, Hosseini A, Naseh I, Gholestanpour A. The effect of lateral trunk motion on the severity of knee osteoarthritis. Biomed Pharmacol J. 2015;8:139–45.

14. Fenner V, Behrend H, Kuster MS. Whole body gait function during stair ascending and level walking in patients following total knee arthroplasty. Int J Phys Med Rehabil. 2014;2:1–9.

15. Boonstra MC, De Waal Malefijt MC, Verdonschot N. How to quantify knee function after total knee arthroplasty? Knee. 2008;15:390–5.

16. Saari T, Tranberg R, Zügner R, Uvehammer J, Kärrholm J. The effect of tibial insert design on rising from a chair; motion analysis after total knee replacement. Clin Biomech. 2004;19:951–6.

17. Pozzi F, Marmon AR, Snyder-Mackler L, Zeni J. Lower leg compensatory strategies during performance of a step up and over task in patient six-months after total knee arthroplasty. Gait Posture. 2016;49:41–6.

18. Claudon B, Poussel M, Billon-Grumillier C, Beyaert C, Paysant J. Knee kinetic pattern during gait and anterior knee pain before and after rehabilitation in patients with patellofemoral pain syndrome. Gait Posture. 2012;36:139–43.

19. Bramah C, Preece SJ, Gill N, Herrington L. Is there a pathological gait associated with common soft tissue running injuries? Am J Sports Med. 2018;46:3023–31.

20. Ho K-Y, Barrett T, Clark Z, DuVall C, Fox T, Howden C, et al. Comparisons of trunk and knee mechanics during various speeds of treadmill running between runners with and without patellofemoral pain: a preliminary study. J Phys Ther Sci. 2021;33:737–41.

21. Nunes GS, Wolf DF, dos Santos DA, de Noronha M, Serrão FV. Acute effects of hip mobilization with movement technique on pain and biomechanics in females with patellofemoral pain: A randomized, placebo-controlled trial. J Sport Rehabil. 2020;29:707–15.

22. Glaviano NR, Baellow A, Saliba S. Elevated fear avoidance affects lower extremity strength and squatting kinematics in women with patellofemoral pain. Athl Train Sport Heal Care. 2019;11:192–200.

23. Hebert LJ, Gravel D, Arsenault a B, Tremblay G. Patellofemoral pain syndrome: the possible role of an inadequate neuromuscular mechanism. Clin Biomech. 1994;9:93–7.

24. Schwane BG, Goerger BM, Goto S, Blackburn T, Aguilar AJ, Padua DA. Trunk and lower extremity kinematics during stair descent in women with or without patellofemoral pain. J Athl Train. 2015;50:704–12.

25. Nakagawa TH, Moriya ÉTU, MacIel CD, Serrão FV. Frontal plane biomechanics in males and females with and without patellofemoral pain. Med Sci Sports Exerc. 2012;44:1747–55.

26. Bley AS, Correa JCF, Reis AC Dos, Rabelo NDDA, Marchetti PH, Lucareli PRG. Propulsion phase of the single leg triple hop test in women with patellofemoral pain syndrome: A biomechanical study. PLoS One. 2014;9:e97606.

27. Alvim FC, Muniz AM de S, Lucareli PRG, Menegaldo LL. Kinematics and muscle forces in women with patellofemoral pain during the propulsion phase of the single leg triple hop test. Gait Posture. 2019;73:108–15.

28. Bazett-Jones DM, Cobb SC, Huddleston WE, O’Connor KM, Armstrong BSR, Earl-Boehm JE. Effect of patellofemoral pain on strength and mechanics after an exhaustive run. Med Sci Sports Exerc. 2013;45:1331–9.

29. Novello A de A, Garbelotti S, Rabelo ND dos A, Ferraz AN, Bley AS, Correa JCF, et al. Descending stairs: Good or bad task to discriminate women with patellofemoral pain? Gait Posture. 2018;65:26–32.

30. Noehren B, Abraham A, Curry M, Johnson D, Ireland ML. Evaluation of proximal joint kinematics and muscle strength following ACL reconstruction surgery in female athletes. J Orthop Res. 2014;23:1305–10.

31. Boggess G, Morgan K, Johnson D, Ireland ML, Reinbolt JA, Noehren B. Neuromuscular compensatory strategies at the trunk and lower limb are not resolved following an ACL reconstruction. Gait Posture. 2018;60:81–7.

32. Slater L V., Blemker SS, Hertel J, Saliba SA, Weltman AL, Hart JM. Sex affects gait adaptations after exercise in individuals with anterior cruciate ligament reconstruction. Clin Biomech. 2020;71:189–95.

33. Scarneo-Miller SE, Sorge JE, Beltz EM, Martinez JC, Root HJ, Burland JP, et al. The relationship between single-limb squat and jump-cut kinematics. Sport Biomech. 2019;11:1–12.

34. Bell DR, Kulow SM, Stiffler MR, Smith MD. Squatting mechanics in people with and without anterior cruciate ligament reconstruction: The influence of graft type. Am J Sports Med. 2014;42:2979–87.

35. Kotsifaki A, Van Rossom S, Whiteley R, Korakakis V, Bahr R, Sideris V, et al. Symmetry in triple hop distance hides asymmetries in knee function after ACL reconstruction in athletes at return to sports. Am J Sports Med. 2021;50:441–50.

36. Kotsifaki A, Van Rossom S, Whiteley R, Korakakis V, Bahr R, Sideris V, et al. Single leg vertical jump performance identifies knee function deficits at return to sport after ACL reconstruction in male athletes. Br J Sports Med. 2022;

37. Smeets A, Vanrenterghem J, Staes F, Vandenneucker H, Claes S, Verschueren S. Are anterior cruciate ligament-reconstructed athletes more vulnerable to fatigue than uninjured athletes? Med Sci Sports Exerc. 2020;52:345–53.

38. Davies JL, Button K, Sparkes V, van Deursen RW. Frontal plane movement of the pelvis and thorax during dynamic activities in individuals with and without anterior cruciate ligament injury. Knee. 2018;25:997–1008.

39. Fukuda W, Kawamura K, Yokoyama S, Kataoka Y, Ikeno Y, Chikaishi N, et al. Joint movement variability during landing in patients with anterior cruciate ligament reconstruction. J Sports Med Phys Fitness. 2021;61:1629–35.

40. Sritharan P, Perraton LG, Munoz MA, Pivonka P, Bryant AL. Muscular coordination of single-leg hop landing in uninjured and anterior cruciate ligament-reconstructed individuals. J Appl Biomech. 2020;36:235–43.

**Additional file 1F** Unpooled data of included studies

**Table F1** Trunk kinematics, knee OA versus controls

|  |  | **Sagittal plane - Flexion** | | | | | |
| --- | --- | --- | --- | --- | --- | --- | --- |
|  | Authors | Variable of interest | Knee OA | N | Controls | N | SMD (95% CI) |
| **Stair ascent** | | | | | | | |
|  | MQ Meireles et al. 2019  [KL 2-3] | Trunk flexion at first peak knee contact force | 23.71±3.31 | 10 | 18.1±3.26 | 16 | 1.66 (.73 to 2.59) |
| **Stair descent** | | | | | | | |
|  | MQ Meireles et al. 2019  [KL 2-3] | Trunk flexion at first peak knee contact force | 11.5±3.64 | 10 | 0.01±9.29 | 16 | 1.45 (.55 to 2.35) |
| **Walking** | | | | | | | |
|  | HQ Sparkes et al. 2019  [KL 2-3] | Trunk flexion RoM (stance phase) | NR | 8 | NR | 8 | UTD |
|  | MQ Preece and Alghamdi 2021  [KL 1-4] | Mean trunk flexion (15-25% stance phase) | 4.2±NR | 20 | 1.6±NR | 20 | UTD |
|  |  | **Sagittal plane – Other** | | | | | |
|  | Authors | Variable of interest | Knee OA | N | Controls | N | SMD (95% CI) |
| **Forward lunge** | | | | | | | |
|  | MQ Van der Straaten et al. 2020b  [KL 3-4] | Trunk angle in the sagittal plane | Waveform analysis | 19 | Waveform analysis | 12 | UTD |
| **Quiet standing task** | | | | | | | |
|  | MQ Turcot et al. 2015  [KL 4] | Mean trunk angle in the sagittal plane | 1.4±6.3 | 87 | -1.5±4.7 | 25 | .48 (.03 to .93) |
| **Sideward lunge** | | | | | | | |
|  | MQ Van der Straaten et al. 2020b  [KL 3-4] | Trunk angle in the sagittal plane | Waveform analysis | 19 | Waveform analysis | 12 | UTD |
| **Single leg squat** | | | | | | | |
|  | MQ Van der Straaten et al. 2020b  [KL 3-4] | Trunk angle in the sagittal plane | Waveform analysis | 19 | Waveform analysis | 12 | UTD |
| **Sit-to-stand** | | | | | | | |
|  | MQ Van der Straaten et al. 2020b  [KL 3-4] | Trunk angle in the sagittal plane | Waveform analysis | 19 | Waveform analysis | 12 | UTD |
| **Stair ascent** | | | | | | | |
|  | MQ Van der Straaten et al. 2020b  [KL 3-4] | Trunk angle in the sagittal plane (stance and swing phases) | Waveform analysis | 19 | Waveform analysis | 12 | UTD |
| **Stair descent** | | | | | | | |
|  | MQ Van der Straaten et al. 2020b  [KL 3-4] | Trunk angle in the sagittal plane (stance and swing phases) | Waveform analysis | 19 | Waveform analysis | 12 | UTD |
| **Unipodal stance task** | | | | | | | |
|  | MQ Van der Straaten et al. 2020a  [KL 3-4] | Trunk angle in the sagittal plane | Waveform analysis | 19 | Waveform analysis | 12 | UTD |
| **Walking** | | | | | | | |
|  | MQ Preece et al. 2019  [KL NR] | Trunk angle in the sagittal plane (stance phase) | Waveform analysis | 27 | Waveform analysis | 19 | UTD |
|  | MQ Turcot et al. 2013  [KL 4] | Mean trunk angle in the sagittal plane | 5.39±7.21 | 60 | 3.31±4.81 | 26 | .31 (-.15 to .78) |
|  | MQ Van der Straaten et al. 2020b  [KL 3-4] | Trunk angle in the sagittal plane (stance and swing phases) | Waveform analysis | 19 | Waveform analysis | 12 | UTD |
|  |  | **Frontal plane – Ipsilateral lean** | | | | | |
|  | Authors | Variable of interest | Knee OA | N | Controls | N | SMD (95% CI) |
| **Stair ascent** | | | | | | | |
|  | MQ Meireles et al. 2019  [KL 2-3] | Ipsilateral trunk lean at first peak knee contact force | 3.06±2.14 | 10 | 0.83±2.92 | 16 | .81 (-.01 to 1.64) |
| **Stair descent** | | | | | | | |
|  | MQ Meireles et al. 2019  [KL 2-3] | Ipsilateral trunk lean at first peak knee contact force | 0.57±2.01 | 10 | -1.0±2.42 | 16 | .67 (-.15 to 1.48) |
| **Walking** | | | | | | | |
|  | MQ Hálfdanardóttir et al. 2018  [KL 2-3] | Ipsilateral trunk lean at IC | 0.00±1.0 | 17 | 1.4±1.4 | 14 | -1.14 (-1.91 to -.37) |
|  | MQ Linley et al. 2010  [KL 1-4] | Ipsilateral trunk lean at first peak of VGRF | 1.9±2.0 | 40 | 1.9±1.6 | 40 | .00 (-.44 to .44) |
|  | MQ Linley et al. 2010  [KL 1-4] | Ipsilateral trunk lean at second peak of VGRF | 3.3±3.5 | 40 | 1.9±5.3 | 40 | .31 (-.13 to .75) |
|  | MQ Linley et al. 2010  [KL 1-4] | Ipsilateral trunk lean at midstance point (50% of stance phase) | 0.9±2.2 | 40 | 0.8±1.8 | 40 | .05 (-.39 to .49) |
|  | MQ Kean et al. 2013  [KL 2-4] | Ipsilateral trunk lean at peak knee adduction moment | 1.88±2.53 | 30 | 0.91±2.09 | 30 | .41 (-.10 to .92) |
|  |  | **Frontal plane – Contralateral lean** | | | | | |
|  | Authors | Variable of interest | Knee OA | N | Controls | N | SMD (95% CI) |
| **Walking** | | | | | | | |
|  | MQ Hálfdanardóttir et al. 2018**^‡*^**  [KL 2-3] | Contralateral trunk lean (end of stance phase) | 2.50±1.32 | 17 | 2.80±1.63 | 14 | -.20 (-.91 to .51) |
|  |  | **Frontal plane – Other** | | | | | |
|  | Authors | Variable of interest | Knee OA | N | Controls | N | SMD (95% CI) |
| **Forward lunge** | | | | | | | |
|  | MQ Van der Straaten et al. 2020b  [KL 3-4] | Trunk lean in the frontal plane | Waveform analysis | 19 | Waveform analysis | 12 | UTD |
| **Quiet standing task** | | | | | | | |
|  | MQ Turcot et al. 2015  [KL 4] | Mean lateral trunk lean | NR | 87 | NR | 25 | UTD |
| **Unipodal standing task** | | | | | | | |
|  | MQ Van der Straaten et al. 2020a  [KL 3-4] | Lateral trunk lean | Waveform analysis | 19 | Waveform analysis | 12 | UTD |
| **Sideward lunge** | | | | | | | |
|  | MQ Van der Straaten et al. 2020b  [KL 3-4] | Lateral trunk lean | Waveform analysis | 19 | Waveform analysis | 12 | UTD |
| **Single leg squat** | | | | | | | |
|  | MQ Van der Straaten et al. 2020b  [KL 3-4] | Lateral trunk lean | Waveform analysis | 19 | Waveform analysis | 12 | UTD |
| **Sit-to-stand** | | | | | | | |
|  | MQ Van der Straaten et al. 2020b  [KL 3-4] | Lateral trunk lean | Waveform analysis | 19 | Waveform analysis | 12 | UTD |
| **Stair ascent** | | | | | | | |
|  | HQ Sparkes et al. 2019  [KL 2-3] | Lateral trunk lean RoM (stance phase) | 7.98±3.6 | 8 | 5.15±2.5 | 8 | .86 (-.18 to 1.90) |
|  | MQ Van der Straaten et al. 2020b  [KL 3-4] | Lateral trunk lean (stance and swing phases) | Waveform analysis | 19 | Waveform analysis | 12 | UTD |
| **Stair descent** | | | | | | | |
|  | HQ Sparkes et al. 2019  [KL 2-3] | Lateral trunk lean RoM (stance phase) | 4.53±1 | 8 | 7.98±3.6 | 8 | -1.23 (-2.33 to -.14) |
|  | MQ Van der Straaten et al. 2020b  [KL 3-4] | Lateral trunk lean (stance and swing phases) | Waveform analysis | 19 | Waveform analysis | 12 | UTD |
| **Walking** | | | | | | | |
|  | LQ Azma et al. 2015  [KL NR] | Lateral trunk lean at first peak of VGRF | 1.9±0.2 | 16 | 1.9±1.6 | 10 | .00 (-.79 to .79) |
|  | LQ Azma et al. 2015  [KL NR] | Mean lateral trunk lean (midstance) | 0.9±2.2 | 16 | 0.8±1.8 | 10 | .05 (-.74 to .84) |
|  | MQ Linley et al. 2010  [KL 1-4] | Mean lateral trunk lean at 20-80% of stance | 2.8±3.3 | 40 | 1.8±5.4 | 40 | .22 (-.22 to .66) |
|  | MQ Magalhães et al. 2013  [KL 2-3] | Lateral trunk lean (stance phase) | Waveform analysis | 40 | Waveform analysis | 40 | UTD |
|  | MQ Preece et al. 2019  [KL NR] | Lateral trunk lean (stance phase) | Waveform analysis | 27 | Waveform analysis | 19 | UTD |
|  | HQ Sparkes et al. 2019  [KL 2-3] | Lateral trunk lean RoM (stance phase) | NR | 8 | NR | 8 | UTD |
|  | MQ Staab et al. 2014  [KL 3-4] | Lateral trunk lean symmetry (stance phase) | 9.4±2.2 | 12 | 4.3±1.9 | 7 | 2.32 (1.07 to 3.57) |
|  | MQ Turcot et al. 2013  [KL 4] | Mean lateral trunk lean (stance phase) | 1.39±2.0 | 60 | 0.31±0.35 | 26 | .63 (.16 to 1.10) |
|  | MQ Van der Straaten et al. 2020b  [KL 3-4] | Lateral trunk lean (stance and swing phases) | Waveform analysis | 19 | Waveform analysis | 12 | UTD |
|  |  | **Transverse plane – Other** | | | | | |
|  | Authors | Variable of interest | Knee OA | N | Controls | N | SMD (95% CI) |
| **Forward lunge** | | | | | | | |
|  | MQ Van der Straaten et al. 2020b  [KL 3-4] | Trunk rotation | Waveform analysis | 19 | Waveform analysis | 12 | UTD |
| **Unipodal stance task** | | | | | | | |
|  | MQ Van der Straaten et al. 2020a  [KL 3-4] | Trunk rotation | Waveform analysis | 19 | Waveform analysis | 12 | UTD |
| **Sideward lunge** | | | | | | | |
|  | MQ Van der Straaten et al. 2020b  [KL 3-4] | Trunk rotation | Waveform analysis | 19 | Waveform analysis | 12 | UTD |
| **Single leg squat** | | | | | | | |
|  | MQ Van der Straaten et al. 2020b  [KL 3-4] | Trunk rotation | Waveform analysis | 19 | Waveform analysis | 12 | UTD |
| **Sit-to-stand** | | | | | | | |
|  | MQ Van der Straaten et al. 2020b  [KL 3-4] | Trunk rotation | Waveform analysis | 19 | Waveform analysis | 12 | UTD |
| **Stair ascent** | | | | | | | |
|  | MQ Meireles et al. 2019  [KL 2-3] | Trunk rotation at first peak knee contact force | NR | 10 | NR | 16 | UTD |
|  | HQ Sparkes et al. 2019  [KL 2-3] | Trunk rotation RoM (stance phase) | 20.26±7.96 | 8 | 13.04±1.86 | 8 | 1.18 (.09 to 2.27) |
|  | MQ Van der Straaten et al. 2020b  [KL 3-4] | Trunk rotation (stance and swing phases) | Waveform analysis | 19 | Waveform analysis | 12 | UTD |
| **Stair descent** | | | | | | | |
|  | MQ Meireles et al. 2019  [KL 2-3] | Trunk rotation at first peak knee contact force | NR | 10 | NR | 16 | UTD |
|  | HQ Sparkes et al. 2019  [KL 2-3] | Trunk rotation RoM (stance phase) | 5.15±2.5 | 8 | 20.27±7.96 | 8 | -2.42 (-3.80 to -1.05) |
|  | MQ Van der Straaten et al. 2020b  [KL 3-4] | Trunk rotation (stance and swing phases) | Waveform analysis | 19 | Waveform analysis | 12 | UTD |
| **Walking** | | | | | | | |
|  | HQ Sparkes et al. 2019  [KL 2-3] | Trunk rotation RoM (stance phase) | NR | 8 | NR | 8 | UTD |
|  | MQ Turcot et al. 2013  [KL 4] | Mean trunk rotation (stance phase) | 0.75±2.92 | 60 | 0.9±0.49 | 26 | -.06 (-.52 to .40) |
|  | MQ Van der Straaten et al. 2020b  [KL 3-4] | Trunk rotation (stance and swing phases) | Waveform analysis | 19 | Waveform analysis | 12 | UTD |

Data reported as mean±SD

The shaded area represents between-groups comparisons with significant difference

**^‡^** Data supplied by author

* Signals are inverted for standardization purpose

^†^ Hálfdanardóttir et al. 2018: data combined for left and right side of controls

*NR* not reported, *OA* osteoarthritis, *KL* *Kellgren-Lawrence* grade, *UTD* unable to determine, *RoM* range of motion, *VGRF* vertical ground reaction force.

**Table F2** Trunk kinematics, TKA versus controls

|  |  | **Sagittal plane - Flexion** | | | | | |
| --- | --- | --- | --- | --- | --- | --- | --- |
|  | Authors | Variable of interest | TKA | N | Controls | N | SMD (95% CI) |
| **Set up and over task** | | | | | | | |
|  | MQ Pozzi et al. 2016 | Trunk flexion at each peak of the total support moment (weight acceptance) | 5.86±11.70 | 19 | 2.84±8.18 | 19 | .29 (-.35 to .93) |
| **Sit-to-stand** | | | | | | | |
|  | HQ Boonstra et al. 2008**^‡‡^** | Peak trunk flexion | 35.98±9.8 | 28 | 36.19±9.22 | 31 | -.02 (-.53 to .49) |
|  | HQ Boonstra et al. 2008**^‡‡^** | Peak trunk flexion angular velocity | 61.43±28.0 | 28 | 77.64±17.93 | 31 | -.69 (-1.21 to -.16) |
|  | MQ Saari et al. 2004 | Peak trunk flexion | Data as median | 29 | Data as median | 16 | UTD |
| **Stair ascent** | | | | | | | |
|  | MQ Fenner et al. 2014 | Trunk flexion at IC | 14.9±5.0 | 18 | 13.7±6.6 | 20 | .20 (-.44 to .84) |
| **Walking** | | | | | | | |
|  | MQ Fenner et al. 2014 | Trunk flexion at IC | 2.2±3.3 | 18 | 1.3±3.8 | 20 | .25 (-.39 to .89) |
|  | MQ Li et al. 2013 | Trunk flexion (stance phase) | NR | 14 | NR | 40 | UTD |
|  |  | **Frontal plane – Ipsilateral lean** | | | | | |
|  | Authors | Variable of interest | TKA | N | Controls | N | SMD (95% CI) |
| **Walking** | | | | | | | |
|  | MQ Fenner et al. 2014 | Ipsilateral trunk lean at IC | 0.2±2.2 | 18 | 0.4±1.9 | 20 | -.10 (-.73 to .54) |
|  |  | **Frontal plane – Contralateral lean** | | | | | |
|  | Authors | Variable of interest | TKA | N | Controls | N | SMD (95% CI) |
| **Stair ascent** | | | | | | | |
|  | MQ Fenner et al. 2014 | Contralateral trunk lean at IC | 0.6±2.9 | 18 | 0.4±2.6 | 20 | .07 (-.57 to .71) |
|  |  | **Transverse plane – Contralateral rotation** | | | | | |
|  | Authors | Variable of interest | TKA | N | Controls | N | SMD (95% CI) |
| **Stair ascent** | | | | | | | |
|  | MQ Fenner et al. 2014 | Contralateral trunk rotation at IC | 6.1±3.6 | 18 | 6.6±3.8 | 20 | -.13 (-.77 to .51) |
| **Walking** | | | | | | | |
|  | MQ Fenner et al. 2014 | Contralateral trunk rotation at IC | 1.9±2.8 | 18 | 1.8±2.1 | 20 | .04 (-.60 to .68) |

Data reported as mean±SD

The shaded area represents between-groups comparisons with significant difference

**^‡‡^** Data extracted from graphs

*IC* initial contact, *NR* not reported, *RoM* range of motion, *TKA* total knee arthroplasty, *UTD* unable to determine.

**Table F3** Trunk kinematics, PFP versus controls

|  |  | **Sagittal plane - Flexion** | | | | | |
| --- | --- | --- | --- | --- | --- | --- | --- |
|  | Authors | Variable of interest | PFP | N | Controls | N | SMD (95% CI) |
| **Drop vertical jump** | | | | | | | |
|  | MQ Baellow et al. 2020 | Time to peak trunk flexion (landing) | 0.19±0.03 | 15 | 0.19±0.01 | 15 | .00 (-.72 to .72) |
| **Running** | | | | | | | |
|  | HQ Bramah et al. 2018**^‡^** | Trunk flexion at IC | 4.78±3.96 | 18 | 3.9±2.9 | 36 | .26 (-.30 to .83) |
|  | HQ Ho et al. 2021 | Trunk flexion at peak PFJ stress | 4.0±3.7 | 7 | 4.5±5.1 | 5 | -.11 (-1.26 to 1.04) |
| **Single leg drop vertical jump** | | | | | | | |
|  | HQ Nunes et al. 2020 | Trunk flexion at peak knee flexion angle (landing) | 14±11.3 | 28 | 18.6±11.57 | 28 | -.40 (-.93 to .13) |
| **Single leg squat** | | | | | | | |
|  | MQ Glaviano et al. 2019 (1) | Trunk flexion RoM | 17.1±7.7 | 9 | 15.8±5.7 | 9 | .18 (-.74 to 1.11) |
|  | MQ Glaviano et al. 2019 (2) | Trunk flexion RoM | 12.1±5.4 | 7 | 15.8±5.7 | 9 | -.63 (-1.65 to .39) |
|  | HQ Nunes et al. 2020 | Trunk flexion at peak knee flexion angle (stance phase) | 18.05±8.08 | 28 | 16.09±9.13 | 28 | .13 (-.30 to .75) |
| **Single leg triple hop task** | | | | | | | |
|  | HQ Bley et al. 2014 | Peak trunk flexion (jumping) | 14.3±4.7 | 20 | 8.8±3.4 | 20 | 1.31 (.62 to 2.00) |
|  | HQ Dos Reis et al. 2015 | Time to peak trunk flexion (landing) | 69±3 | 20 | 61±3 | 20 | 2.61 (1.75 to 3.48) |
| **Squatting** | | | | | | | |
|  | LQ Hébert et al. 1994**^‡^** | Two-dimensional trunk flexion displacement | 43.5±6.7 | 11 | 41.5±4.1 | 11 | .35 (-.50 to 1.19) |
| **Walking** | | | | | | | |
|  | HQ Claudon et al. 2012 | Trunk flexion at peak KEM | 9.1±2.7 | 23 | 5.8±3.2 | 22 | 1.10 (.47 to 1.73) |
|  |  | **Sagittal plane – Other** | | | | | |
| **Single leg triple hop task** | | | | | | | |
|  | MQ Alvim et al. 2019 | Trunk sagittal plane angle (jumping) | Waveform analysis | 11 | Waveform analysis | 10 | UTD |
|  |  | **Frontal plane – Ipsilateral lean** | | | | | |
|  | Authors | Variable of interest | PFP | N | Controls | N | SMD (95% CI) |
| **Drop vertical jump** | | | | | | | |
|  | MQ Baellow et al. 2020 | Time to peak ipsilateral trunk lean (landing) | 0.11±0.04 | 15 | 0.12±0.07 | 15 | -.17 (-.89 to .55) |
| **Running** | | | | | | | |
|  | HQ Bramah et al. 2018**^‡^** | Ipsilateral trunk lean at IC | 3.09±2.55 | 18 | 2.5±1.8 | 36 | .28 (-.29 to .85) |
| **Single leg drop vertical jump** | | | | | | | |
|  | HQ Nunes et al. 2020 | Ipsilateral trunk lean at peak knee flexion angle (landing) | 8.55±4.24 | 28 | 5.65±4.03 | 28 | .69 (.15 to 1.23) |
| **Single leg squat** | | | | | | | |
|  | HQ Nunes et al. 2020 | Ipsilateral trunk lean at peak knee flexion (landing) | 7.55±3.3 | 28 | 6.2±3.29 | 28 | .40 (-.13 to .93) |
| **Single leg triple hop task** | | | | | | | |
|  | HQ Bley et al. 2014 | Peak ipsilateral trunk lean (jumping) | 5.6±4.7* | 20 | -6.5±4.4* | 20 | 2.61 (1.74 to 3.47) |
|  | HQ Dos Reis et al. 2015 | Time to peak ipsilateral trunk lean (landing) | 38±1 | 20 | 28±2 | 20 | 6.20 (4.64 to 7.76) |
|  |  | **Frontal plane – contralateral lean** | | | | | |
|  | Authors | Variable of interest | PFP | N | Controls | N | SMD (95% CI) |
| **Running** | | | | | | | |
|  | MQ Bazett-Jones et al. 2013 | Peak contralateral trunk lean (stance phase) | 2.7±4.1 | 19 | 2.5±2.2 | 19 | .06 (-.58 to .70) |
| **Stair descent** | | | | | | | |
|  | MQ Novello et al. 2018 | Contralateral trunk lean at IC | 2.6±2.4 | 34 | 2.8±2.0 | 34 | -.09 (-.57 to .39) |
|  | MQ Novello et al. 2018 | Contralateral trunk lean in load response | 1.1±2.2 | 34 | 1.4±2.4 | 34 | -.13 (-.60 to .35) |
|  |  | **Frontal plane - Other** | | | | | |
| **Single leg triple hop task** | | | | | | | |
|  | MQ Alvim et al. 2019 | Lateral trunk lean (jumping) | Waveform analysis | 11 | Waveform analysis | 10 | UTD |
|  |  | **Transverse plane – Ipsilateral rotation** | | | | | |
|  | Authors | Variable of interest | PFP | N | Controls | N | SMD (95% CI) |
| **Drop vertical jump** | | | | | | | |
|  | MQ Baellow et al. 2020 | Time to peak ipsilateral trunk rotation (landing) | 0.08±0.05 | 15 | 0.09±0.05 | 15 | -.19 (-.91 to .52) |
| **Single leg triple hop task** | | | | | | | |
|  | HQ Dos Reis et al. 2015 | Time to peak ipsilateral trunk rotation (landing) | 12±1 | 20 | 17±5 | 20 | -1.36 (-2.05 to -.66) |
| **Stair descent** | | | | | | | |
|  | HQ Schwane et al. 2015 | Ipsilateral trunk rotation RoM (stance phase) | 3±3.2 | 20 | 3.6±4.3 | 20 | -.16 (-.78 to .47) |
|  |  | **Transverse plane – Contralateral rotation** | | | | | |
|  | Authors | Variable of interest | PFP | N | Controls | N | SMD (95% CI) |
| **Stair descent** | | | | | | | |
|  | HQ Schwane et al. 2015 | Contralateral trunk rotation RoM (stance phase) | 5.5±4.6 | 20 | 4.7±4.3 | 20 | .18 (-.45 to .80) |
|  |  | **Transverse plane – Other** | | | | | |
|  | Authors | Variable of interest | PFP | N | Controls | N | SMD (95% CI) |
| **Single leg triple hop task** | | | | | | | |
|  | MQ Alvim et al. 2019 | Trunk rotation (landing) | Waveform analysis | 11 | Waveform analysis | 10 | UTD |

Data reported as mean±SD

The shaded area represents between-groups comparisons with significant difference

**^‡^** Data supplied by author

* Signals are inverted for standardization purpose

(1) PFP group with elevated fear avoidance beliefs, (2) PFP group with low fear avoidance beliefs

*IC* initial contact, *KEM* knee extensor moment, *NR* not reported, *PFJ* patellofemoral joint, *PFP* patellofemoral pain, *RoM* range of motion, *UTD* unable to determine.

**Table F4** Trunk kinematics, PT versus controls

|  |  | **Sagittal plane - Flexion** | | | | | |
| --- | --- | --- | --- | --- | --- | --- | --- |
|  | Authors | Variable of interest | PT | N | Controls | N | SMD (95% CI) |
| **Decline squat** | | | | | | | |
|  | MQ Barker-Davies et al. 2019 | Trunk flexion at peak knee flexion angle | 17.1±9.1 | 21 | 18.1±9.4 | 22 | -.11 (-.70 to .49) |
| **Single leg squat** | | | | | | | |
|  | MQ Barker-Davies et al. 2019 | Trunk flexion at peak knee flexion anglr | 11.9±4.6 | 21 | 16.4±11.1 | 22 | -.52 (-1.12 to .09) |
| **Small knee bend** | | | | | | | |
|  | MQ Barker-Davies et al. 2019 | Trunk flexion at peak knee flexion angle | 8.5±4.8 | 21 | 5.8±5.3 | 22 | .52 (-.09 to 1.13) |
| **Drop vertical landing** | | | | | | | |
|  | HQ Scattone et al. 2017 | Trunk flexion at IC | 7.7±5 | 7 | 3.8±6.4 | 7 | .64 (-.45 to 1.72) |
|  | HQ Scattone et al. 2017 | Peak trunk flexion | 28.5±10.4 | 7 | 24.9±5 | 7 | .41 (-.65 to 1.48) |

Data reported as mean±SD

*IC* initial contact, *PT* patellar tendinopathy.

**Table F5** Trunk kinematics, ACLD versus controls

|  |  | **Sagittal plane - Flexion** | | | | | |
| --- | --- | --- | --- | --- | --- | --- | --- |
|  | Authors | Variable of interest | ACLD | N | Controls | N | SMD (95% CI) |
| **Landing and cutting tasks** | | | | | | | |
|  | MQ Hewett et al. 2009 | Two-dimensional trunk flexion displacement | 1.6±9.3 | 10 | 14±7.3 | 6 | -1.36 (-2.50 to -.21) |
| **Single leg landing** | | | | | | | |
|  | MQ Sipprell et al. 2012 | Two-dimensional trunk flexion displacement | 4±14 | 20 | 16±13 | 20 | -.87 (-1.52 to -.22) |
| **Single leg vertical hop** | | | | | | | |
|  | MQ Markstrom et al. 2018 | Peak trunk flexion (jumping) | 37.7±11 | 34 | 39.1±9.6 | 33 | -.13 (-.61 to .35) |
|  | MQ Markstrom et al. 2018 | Peak trunk flexion (landing) | 23.8±7.7 | 34 | 23.6±9.9 | 33 | .02 (-.46 to .50) |
| **Stair ascent** | | | | | | | |
|  | MQ Shi et al. 2016 | Peak trunk flexion (stance phase) | 7.9±0.7 | 36 | 7±0.7 | 36 | 1.27 (.76 to 1.78) |
|  |  | **Sagittal plane - Other** | | | | | |
|  | Authors | Variable of interest | ACLD | N | Controls | N | SMD (95% CI) |
| **Single leg hop for distance** | | | | | | | |
|  | MQ Oberlander et al. 2012 | Mean trunk angle in the sagittal plane (landing) | NR | 12 | NR | 13 | UTD |
|  |  | **Sagittal plane - Extension** | | | | | |
|  | Authors | Variable of interest | ACLD | N | Controls | N | SMD (95% CI) |
| **Stair descent** | | | | | | | |
|  | MQ Shi et al. 2016 | Peak trunk extension (stance phase) | 1.13±0.7 | 36 | 2.3±0.4 | 36 | -2.03 (-2.60 to -1.46) |
| **Walking** | | | | | | | |
|  | MQ Shi et al. 2016 | Peak trunk extension (stance phase) | -0.8±0.3 | 36 | -0.8±0.5 | 36 | .00 (-.46 to .46) |
|  |  | **Frontal plane –Ipsilateral lean** | | | | | |
|  | Authors | Variable of interest | ACLD | N | Controls | N | SMD (95% CI) |
| **Single leg squat** | | | | | | | |
|  | MQ Fukuda et al. 2021a | Ipsilateral trunk lean RoM | 7.6±4.5 | 56 | 5.8±3.4 | 46 | .44 (.05 to .84) |
|  |  | **Frontal plane – Contralateral lean** | | | | | |
|  | Authors | Variable of interest | ACLD | N | Controls | N | SMD (95% CI) |
| **Single leg hop for distance** | | | | | | | |
|  | MQ Davies et al. 2018 | Peak contralateral trunk lean (landing) | NR | 20 | NR | 32 | UTD |
|  | MQ Davies et al. 2018 | Contralateral trunk lean RoM (landing) | 11±4 | 20 | 19±3 | 32 | -2.31 (-3.03 to -1.58) |
| **Single leg squat** | | | | | | | |
|  | MQ Davies et al. 2018 | Peak contralateral trunk lean (landing) | NR | 28 | NR | 32 | UTD |
|  | MQ Davies et al. 2018 | Contralateral trunk lean RoM (landing) | NR | 28 | NR | 32 | UTD |
| **Stair ascent** | | | | | | | |
|  | MQ Shi et al. 2016 | Peak contralateral trunk lean (stance phase) | 1.9±0.2 | 36 | 0.7±0.3 | 36 | 4.66 (3.75 to 5.56) |
| **Stair descent** | | | | | | | |
|  | MQ Shi et al. 2016 | Peak contralateral trunk lean (stance phase) | 3.9±0.3 | 36 | 3.2±0.1 | 36 | 3.10 (2.40 to 3.79) |
| **Running** | | | | | | | |
|  | MQ Davies et al. 2018 | Peak contralateral trunk lean (stance phase) | NR | 28 | NR | 32 | UTD |
|  | MQ Davies et al. 2018 | Contralateral trunk lean RoM (stance phase) | NR | 28 | NR | 32 | UTD |
| **Walking** | | | | | | | |
|  | MQ Davies et al. 2018 | Peak contralateral trunk lean (stance phase) | NR | 28 | NR | 32 | UTD |
|  | MQ Davies et al. 2018 | Contralateral trunk lean RoM (stance phase) | NR | 28 | NR | 32 | UTD |
|  | MQ Shi et al. 2016 | Peak contralateral trunk lean (stance phase) | 1.5±0.2 | 36 | 1.7±0.1 | 36 | -1.25 (-1.76 to -.74) |
|  |  | **Frontal plane – Other** | | | | | |
|  | Authors | Variable of interest | ACLD | N | Controls | N | SMD (95% CI) |
| **Landing and cutting tasks** | | | | | | | |
|  | MQ Hewett et al. 2009 | Two-dimensional lateral trunk displacement | 11.1±2 | 10 | -5.5±9.5 | 6 | 2.66 (1.19 to 4.13) |
|  |  | **Transverse plane – Ipsilateral rotation** | | | | | |
|  | Authors | Variable of interest | ACLD | N | Controls | N | SMD (95% CI) |
| **Walking** | | | | | | | |
|  | MQ Shi et al. 2016 | Peak ipsilateral trunk rotation (stance phase) | 5.5±0.6 | 36 | 1.6±0.7 | 36 | 5.92 (4.82 to 7.01) |
|  |  | **Transverse plane – Contralateral rotation** | | | | | |
| **Stair ascent** | | | | | | | |
|  | MQ Shi et al. 2016 | Peak contralateral trunk rotation (stance phase) | -24.6±0.8 | 36 | -32.2±0.7 | 36 | 10.00 (8.26 to 11.74) |
| **Stair descent** | | | | | | | |
|  | MQ Shi et al. 2016 | Peak contralateral trunk rotation (stance phase) | -25.9±1.1 | 36 | -33.6±1.0 | 36 | 7.25 (5.94 to 8.55) |

Data reported as mean±SD

The shaded area represents between-groups comparisons with significant difference

*ACLD* anterior cruciate ligament deficiency, *NR* not reported, *RoM* range of motion, *UTD* unable to determine.

**Table F6** Trunk kinematics, ACLR versus controls

|  |  | **Sagittal plane - Flexion** | | | | | |
| --- | --- | --- | --- | --- | --- | --- | --- |
|  | Authors | Variable of interest | ACLR | N | Controls | N | SMD (95% CI) |
| **Running** | | | | | | | |
|  | MQ Armitano et al. 2017 | Acceleration pattern of the lower trunk (stance phase) | NR | 17 | NR | 17 | UTD |
|  | MQ Noehren et al. 2014 | Peak trunk flexion (stance phase) | 6.3±4.9* | 20 | 2.1±3.5* | 20 | .97 (.31 to 1.63) |
| **Single leg drop jump** | | | | | | | |
|  | MQ Kotsifaki et al. 2022 | Peak trunk flexion (landing and jumping) | 27.8±8.4 | 26 | 21.1±9.1 | 22 | .76 (.17 to 1.34) |
| **Single leg squat** |  |  |  |  |  |  |  |
|  | MQ Bell et al. 2014 (1) | Trunk flexion at peak knee flexion angle | 19.35±14.25 | 34 | 17.85±15.64 | 51 | .10 (-.34 to .53) |
|  | MQ Bell et al. 2014 (2) | Trunk flexion at peak knee flexion angle | 28.90±11.78 | 21 | 17.85±15.64 | 51 | .75 (.22 to 1.27) |
|  | MQ Scarneo-Miller et al. 2019 | Peak trunk flexion | NR | 23 | NR | 23 | UTD |
|  | MQ Scarneo-Miller et al. 2019 | Trunk flexion RoM | 11.67±6.45 | 23 | 16.74±7.99 | 23 | -.69 (-1.28 to -.09) |
| **Single leg jump and cut** | | | | | | | |
|  | MQ Scarneo-Miller et al. 2019 | Trunk flexion RoM (landing) | NR | 23 | NR | 23 | UTD |
| **Single leg triple hop distance** | | | | | | | |
|  | MQ Kotsifaki et al. 2021b | Peak trunk flexion (1^st^ and 2^nd^ rebounds) | 44.6±9.07 | 24 | 35.6±8.3 | 26 | 1.01 (.40 to 1.62) |
|  |  | **Sagittal plane - Other** | | | | | |
| **Lateral hop** | | | | | | | |
|  | MQ Smeets et al. 2020 | Trunk angle in the sagittal plane (landing) | Waveform analysis | 21 | Waveform analysis | 21 | UTD |
| **Medial hop** | | | | | | | |
|  | MQ Smeets et al. 2020 | Trunk angle in the sagittal plane (landing) | Waveform analysis | 21 | Waveform analysis | 21 | UTD |
| **Running** | | | | | | | |
|  | HQ Bogges et al. 2018 | Mean trunk angle in the sagittal plane (stance phase) | 0.5±9.4 | 11 | 0.1±6.7 | 11 | .05 (-.79 to .88) |
|  | MQ Slater et al. 2020 | Trunk angle in the sagittal plane (stance and swing phases) | Waverform analysis | 33 | Waverform analysis | 29 | UTD |
| **Single leg hop for distance** | | | | | | | |
|  | MQ Smeets et al. 2020 | Trunk angle in the sagittal plane (landing) | Waveform analysis | 21 | Waveform analysis | 21 | UTD |
| **Vertical hop with medial or lateral rotation** | | | | | | | |
|  | MQ Smeets et al. 2020 | Trunk angle in the sagittal plane (landing) | Waveform analysis | 21 | Waveform analysis | 21 | UTD |
|  |  | **Frontal plane – Ipsilateral lean** | | | | | |
|  | Authors | Variable of interest | ACLR | N | Controls | N | SMD (95% CI) |
| **Running** | | | | | | | |
|  | MQ Noehren et al. 2014 | Contralateral trunk lean at IC | -2±2.7 | 20 | -3.7±1.9 | 20 | .71 (.07 to 1.36) |
| **Single leg squat** | | | | | | | |
|  | MQ Bell et al. 2014 (1) | Ipsilateral trunk lean at peak knee flexion angle | 0.62±5.74 | 34 | 2.09±5.64 | 51 | -.26 (-.69 to .18) |
|  | MQ Bell et al. 2014 (2) | Ipsilateral trunk lean at peak knee flexion angle | 6.4±6.07 | 21 | 2.09±5.64 | 51 | .74 (.22 to 1.26) |
|  |  | **Frontal plane – Contralateral lean** | | | | | |
|  | Authors | Variable of interest | ACLR | N | Controls | N | SMD (95% CI) |
| **Running** | | | | | | | |
|  | MQ Davies et al. 2018 | Peak contralateral trunk lean (stance phase) | NR | 30 | NR | 32 | UTD |
|  | MQ Davies et al. 2018 | Contralateral trunk lean RoM (stance phase) | NR | 30 | NR | 32 | UTD |
| **Single leg hop for distance** | | | | | | | |
|  | MQ Davies et al. 2018 | Peak contralateral trunk lean (landing) | NR | 30 | NR | 32 | UTD |
|  | MQ Davies et al. 2018 | Contralateral trunk lean RoM (landing) | NR | 30 | NR | 32 | UTD |
| **Single leg squat** | | | | | | | |
|  | MQ Davies et al. 2018 | Peak contralateral trunk lean | NR | 30 | NR | 32 | UTD |
|  | MQ Davies et al. 2018 | Contralateral trunk lean RoM | NR | 30 | NR | 32 | UTD |
| **Walking** | | | | | | | |
|  | MQ Davies et al. 2018 | Peak contralateral trunk lean (stance phase) | NR | 30 | NR | 32 | UTD |
|  | MQ Davies et al. 2018 | Contralateral trunk lean RoM (stance phase) | NR | 30 | NR | 32 | UTD |
|  |  | **Frontal plane – Other** | | | | | |
|  | Authors | Variable of interest | ACLR | N | Controls | N | SMD (95% CI) |
| **Running** | | | | | | | |
|  | HQ Bogges et al. 2018 | Mean lateral trunk lean (stance phase) | 8.5±2.6 | 11 | 5.6±5.6 | 11 | .64 (-.22 to 1.50) |
|  | MQ Slater et al. 2020 | Lateral trunk lean (stance and swing phases) | Waveform analysis | 33 | Waveform analysis | 29 | UTD |
| **Single leg jump and cut** | | | | | | | |
|  | MQ Scarneo-Miller et al. 2019 | Peak lateral trunk lean (landing) | NR | 23 | NR | 23 | UTD |
|  | MQ Scarneo-Miller et al. 2019 | Lateral trunk lean RoM (landing) | NR | 23 | NR | 23 | UTD |
| **Single leg squat** | | | | | | | |
|  | MQ Scarneo-Miller et al. 2019 | Peak lateral trunk lean (landing) | NR | 23 | NR | 23 | UTD |
|  | MQ Scarneo-Miller et al. 2019 | Lateral trunk lean RoM (landing) | NR | 23 | NR | 23 | UTD |
| **Vertical unilateral countermovement jump** | | | | | | | |
|  | LQ Setuain et al. 2015a | Lateral trunk lean RoM (jumping) | -5.00±4.37 | 16 | -2.42±2.21 | 28 | -.80 (-1.44 to -.16) |
|  | MQ Setuain et al. 2015b | Lateral trunk lean RoM (jumping) | 1.37±3.43 | 8 | -6.1±1.92 | 15 | 2.85 (1.60 to 4.10) |
|  | LQ Setuain et al. 2015a | Lateral trunk lean RoM (landing) | 5.16±2.11 | 16 | 1.00±1.67 | 28 | 2.22 (1.44 to 3.00) |
|  | MQ Setuain et al. 2015b | Lateral trunk lean RoM (landing) | 0.29±3.64 | 8 | 7.26±1.24 | 15 | -2.88 (-4.13 to -1.63) |
| **50 cm vertical bilateral drop jump** | | | | | | | |
|  | LQ Setuain et al. 2015a | Lateral trunk lean RoM (jumping) | 1.06±0.86 | 12 | 1.73±0.93 | 32 | -.72 (-1.40 to -.04) |
|  | MQ Setuain et al. 2015b | Lateral trunk lean RoM (jumping) | 0.71±0.6 | 6 | 2.19±0.88 | 15 | -1.74 (-2.85 to -.63) |
|  | LQ Setuain et al. 2015a | Lateral trunk lean RoM (landing) | 1.24±0.84 | 12 | -0.61±0.71 | 32 | 2.43 (1.58 to 3.29) |
|  | MQ Setuain et al. 2015b | Lateral trunk lean RoM (landing) | 9.31±0.93 | 6 | -9.43±3.56 | 15 | 5.82 (3.65 to 7.99) |
| **20 cm vertical unilateral drop jump** | | | | | | | |
|  | LQ Setuain et al. 2015a | Lateral trunk lean RoM (jumping) | -3.39±2.95 | 16 | -0.11±1.81 | 28 | -1.41 (-2.10 to -.72) |
|  | MQ Setuain et al. 2015b | Lateral trunk lean RoM (jumping) | 2.3±3.35 | 8 | -3.58±2.01 | 15 | 2.23 (1.12 to 3.35) |
|  | LQ Setuain et al. 2015a | Lateral trunk lean RoM (landing) | 1.61±2.71 | 16 | 0.41±1.42 | 28 | .60 (-.03 to 1.22) |
|  | MQ Setuain et al. 2015b | Lateral trunk lean RoM (landing) | -1.33±2.78 | 8 | 3.33±1.17 | 15 | -2.40 (-3.55 to -1.26) |
|  |  | **Transverse plane - Other** | | | | | |
|  | Authors | Variable of interest | ACLR | N | Controls | N | SMD (95% CI) |
| **Running** | | | | | | | |
|  | MQ Slater et al. 2020 | Trunk rotation (stance phase) | Waveform analysis | 33 | Waveform analysis | 29 | UTD |
| **Single leg hop for distance** | | | | | | | |
|  | HQ Fukuda et al. 2021b | Trunk rotation RoM (landing) | 4.4±1.8 | 54 | 5.1±1.6 | 44 | -.41 (-.81 to -.00) |
| **Single leg jump and cut** | | | | | | | |
|  | MQ Scarneo-Miller et al. 2019 | Peak trunk rotation (landing) | NR | 23 | NR | 23 | UTD |
|  | MQ Scarneo-Miller et al. 2019 | Trunk rotation RoM (landing) | NR | 23 | NR | 23 | UTD |
| **Single leg squat** | | | | | | | |
|  | MQ Scarneo-Miller et al. 2019 | Peak trunk rotation | NR | 23 | NR | 23 | UTD |
|  | MQ Scarneo-Miller et al. 2019 | Trunk rotation RoM | NR | 23 | NR | 23 | UTD |
| **Vertical unilateral countermovement jump** | | | | | | | |
|  | LQ Setuain et al. 2015a | Trunk rotation RoM (jumping) | 4.00±5.40 | 16 | -7.89±2.83 | 28 | 2.96 (2.07 to 3.85) |
|  | MQ Setuain et al. 2015b | Trunk rotation RoM (jumping) | 2.66±-9.90 | 8 | -5.25±2.36 | 15 | 1.26 (.32 to 2.21) |
|  | LQ Setuain et al. 2015a | Trunk rotation RoM (landing) | 1.34±2.24 | 16 | 1.98±1.62 | 28 | -.34 (-.96 to .28) |
|  | MQ Setuain et al. 2015b | Trunk rotation RoM (landing) | 3.04±-1.61 | 8 | 0.54±1.04 | 15 | 1.91 (.86 to 2.97) |
| **50 cm vertical bilateral drop jump** | | | | | | | |
|  | LQ Setuain et al. 2015a | Trunk rotation RoM (jumping) | 0.30±0.97 | 12 | 2.57±1.52 | 32 | -1.60 (-2.35 to -.85) |
|  | MQ Setuain et al. 2015b | Trunk rotation RoM (jumping) | -0.75±-1.46 | 6 | -1.02±0.66 | 15 | .28 (-.68 to 1.23) |
|  | LQ Setuain et al. 2015a | Trunk rotation RoM (landing) | 0.51±1.05 | 12 | -0.77±0.56 | 32 | 1.74 (.98 to 2.51) |
|  | MQ Setuain et al. 2015b | Trunk rotation RoM (landing) | 32.22±3.75 | 6 | 38.29±2.18 | 15 | -2.17 (-3.37 to -.98) |
| **20 cm vertical unilateral drop jump** | | | | | | | |
|  | LQ Setuain et al. 2015a | Trunk rotation RoM (jumping) | -1.80±3.85 | 16 | -4.64±2.52 | 28 | .91 (.27 to 1.56) |
|  | MQ Setuain et al. 2015b | Trunk rotation RoM (jumping) | -0.46±2.50 | 8 | -5.65±1.71 | 15 | 2.49 (1.32 to 3.66) |
|  | LQ Setuain et al. 2015a | Trunk rotation RoM (landing) | 0.83±1.55 | 16 | 1.00±1.32 | 28 | -.12 (-.73 to .50) |
|  | MQ Setuain et al. 2015b | Trunk rotation RoM (landing) | 0.73±1.31 | 8 | 0.84±0.97 | 15 | -.10 (-.96 to .76) |

Data reported as mean±SD

The shaded area represents between-groups comparisons with significant difference

* Data is positive for standardization purpose

(1) group with ipsilateral bone–patellar tendon–bone autograft, (2) group with ipsilateral semitendinosus and gracilis autograft

*ACLR* anterior cruciate ligament reconstruction, *IC* initial contact, *NR* not reported, *RoM* range of motion, *UTD* unable to determine.

**Table F7** Trunk moment, ACLR versus controls

|  |  | **Sagittal plane** | | | | | |
| --- | --- | --- | --- | --- | --- | --- | --- |
|  | Authors | Variable of interest | ACLR |  | Controls |  | SMD (95% CI) |
| **Medial and lateral hop** | | | | | | | |
|  | MQ Smeets et al. 2020 | Trunk flexion moment from IC until 500 milliseconds after IC (landing) | Waveform analysis | 21 | Waveform analysis | 21 | UTD |
| **Single leg hop for distance** | | | | | | | |
|  | MQ Smeets et al. 2020 | Trunk flexion moment from IC until 500 milliseconds after IC (landing) | Waveform analysis | 21 | Waveform analysis | 21 | UTD |
|  | MQ Sritharan et al. 2020 | Peak trunk flexion moment (landing) | 6.5±3.0 | 65 | 5.0±2.6 | 32 | .52 (.09 to .95) |
| **Vertical hop with 90° of medial or lateral rotation** | | | | | | | |
|  | MQ Smeets et al. 2020 | Trunk flexion moment from IC until 500 milliseconds after IC (landing) | Waveform analysis | 21 | Waveform analysis | 21 | UTD |

Data reported as mean±SD

The shaded area represents between-groups comparisons with significant difference

*ACLR* anterior cruciate ligament reconstruction, *IC* initial contact, *UTD* unable to determine.

**Additional file 1G** Sensitivity analysis

**
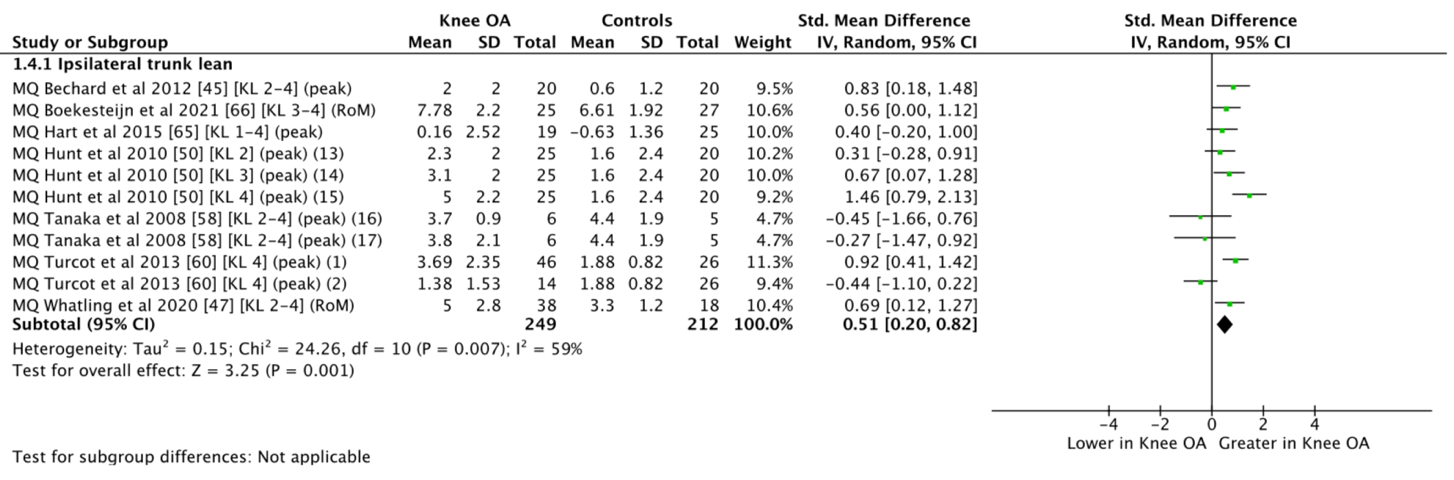
**

**Fig. G1** Meta-analysis of trunk kinematics in the frontal plane during walking in individuals with knee OA compared to controls. (1) varus OA group, (2) valgus OA group, (13) mild OA group, (14) moderate OA group, (15) severe OA group, (16) group with unilateral OA pain, (17) group with bilateral OA pain. *MQ* moderate quality, *OA* osteoarthritis.

**Additional file 1H** Evidence gap map for discrete variables of trunk kinematics and kinetics

| **Kinematics** | | | **Knee disorders** | | | | | |
| --- | --- | --- | --- | --- | --- | --- | --- | --- |
|  |  |  | **Knee OA** | **TKA** | **PFP** | **PT** | **ACLD** | **ACLR** |
| **Running** | | | | | | | | |
| **IC** | Running | Trunk flexion at IC |  |  | 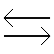 |  |  |  |
|  | Running | Ipsilateral trunk lean at IC |  |  | 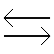 |  |  |  |
|  | Running | Contralateral trunk lean at IC |  |  |  |  |  | 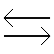 |
| **Peak** | Running | Peak trunk flexion |  |  | 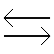 |  |  | 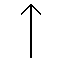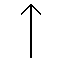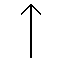 |
|  | Running | Peak ipsilateral trunk lean |  |  | 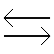 |  |  |  |
|  | Running | Peak contralateral trunk lean |  |  | 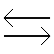 |  | 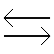 | 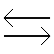 |
| **RoM** | Running | Trunk flexion RoM |  |  | 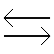 |  |  |  |
|  | Running | Ipsilateral trunk lean RoM |  |  | 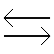 |  |  |  |
|  | Running | Contralateral trunk lean RoM |  |  |  |  | 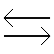 | 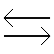 |
| **Other** | Running | Trunk flexion at peak PFJ stress |  |  | 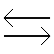 |  |  |  |
| **Walking** | | | | | | | | |
| **IC** | Walking | Trunk flexion at IC |  | 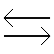 |  |  |  |  |
|  | Walking | Ipsilateral trunk lean at IC | 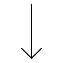 | 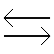 |  |  |  |  |
|  | Walking | Contralateral trunk rotation at IC |  | 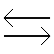 |  |  |  |  |
| **Peak** | Walking | Peak trunk flexion | 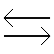 |  |  |  |  |  |
|  | Walking | Peak trunk extension |  |  |  |  | 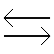 |  |
|  | Walking | Peak ipsilateral trunk lean | 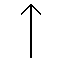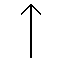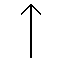 |  |  |  |  |  |
|  | Walking | Peak contralateral trunk lean | 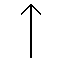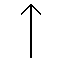 |  |  |  |  | 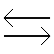 |
|  | Walking | Peak ipsilateral trunk rotation | 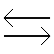 |  |  |  | 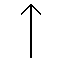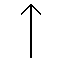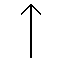 |  |
| **RoM** | Walking | Trunk flexion RoM | 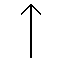 |  |  |  |  |  |
|  | Walking | Ipsilateral trunk lean RoM | 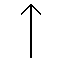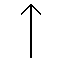 |  |  |  |  |  |
|  | Walking | Contralateral trunk lean RoM |  |  |  |  | 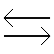 | 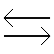 |
|  | Walking | Ipsilateral trunk rotation RoM | 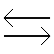 |  |  |  |  |  |
| **Other** | Walking | Trunk flexion at peak KEM |  |  | 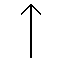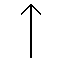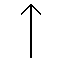 |  |  |  |
|  | Walking | Mean trunk flexion(15-25% stance phase) | 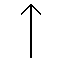 |  |  |  |  |  |
|  | Walking | Ipsilateral trunk lean at first peak of VGRF | 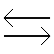 |  |  |  |  |  |
|  | Walking | Ipsilateral trunk lean at midstance point | 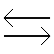 |  |  |  |  |  |
|  | Walking | Ipsilateral trunk lean at second peak of VGRF | 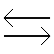 |  |  |  |  |  |
|  | Walking | Ipsilateral trunk lean at peak KAM | 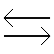 |  |  |  |  |  |
|  | Walking | Contralateral trunk lean(end of stance phase) | 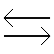 |  |  |  |  |  |
| **Squatting tasks** | | | | | | | | |
| **Peak** | Single leg squat | Peak trunk flexion | 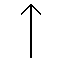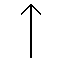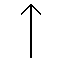 |  |  |  |  | 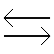 |
|  | Sit-to-stand | Peak trunk flexion |  | 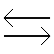 |  |  |  |  |
|  | Sit-to-stand | Peak ipsilateral trunk lean | 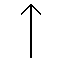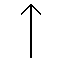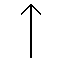 |  |  |  |  |  |
|  | Single leg squat | Peak contralateral trunk lean |  |  |  |  | 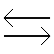 | 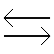 |
| **RoM** | Single leg squat | Trunk flexion RoM |  |  | 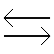 |  |  | 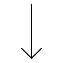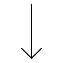 |
|  | Single leg squat | Ipsilateral trunk lean RoM |  |  |  |  | 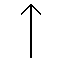 |  |
|  | Single leg squat | Contralateral trunk lean RoM |  |  |  |  | 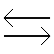 | 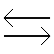 |
|  | Stand-to-sit | Trunk flexion RoM | 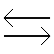 |  |  |  |  |  |
| **Other** | Decline squat | Trunk flexion at peak knee flexion angle |  |  |  | 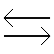 |  |  |
|  | Single leg squat | Trunk flexion at peak knee flexion angle |  |  | 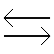 | 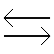 |  | 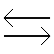 |
|  | Small knee bend | Trunk flexion at peak knee flexion angle |  |  |  | 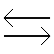 |  |  |
|  | Single leg squat | Ipsilateral trunk lean at peak knee flexion angle |  |  | 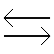 |  |  | 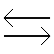 |
|  | Sit-to-stand | Peak trunk flexion angular velocity |  | 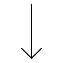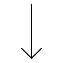 |  |  |  |  |
|  | Squatting | Two-dimensional trunk flexion |  |  | 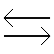 |  |  |  |
| **Stepping tasks** | | | | | | | | |
| **IC** | Stair ascent | Trunk flexion at IC |  | 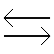 |  |  |  |  |
|  | Stair ascent | Contralateral trunk lean at IC |  | 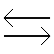 |  |  |  |  |
|  | Stair descent | Contralateral trunk lean at IC |  |  | 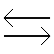 |  |  |  |
|  | Stair ascent | Contralateral trunk rotation at IC |  | 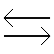 |  |  |  |  |
|  | Stair descent | Contralateral trunk lean in load response |  |  | 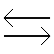 |  |  |  |
| **Peak** | Stair ascent | Peak trunk flexion | 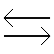 |  |  |  | 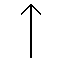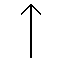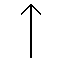 |  |
|  | Stair descent | Peak trunk extension |  |  |  |  | 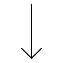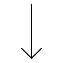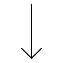 |  |
|  | Stair ascent | Peak contralateral trunk lean |  |  |  |  | 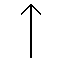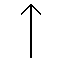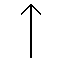 |  |
|  | Stair descent | Peak contralateral trunk lean |  |  |  |  | 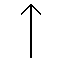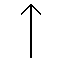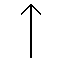 |  |
|  | Stair ascent | Peak contralateral trunk rotation |  |  |  |  | 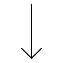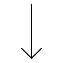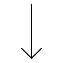 |  |
|  | Stair descent | Peak contralateral trunk rotation |  |  |  |  | 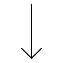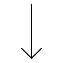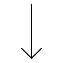 |  |
| **RoM** | Stair ascent | Trunk flexion RoM | 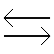 |  |  |  |  |  |
|  | Stair descent | Trunk flexion RoM |  |  |  |  |  |  |
|  | Stair descent | Ipsilateral trunk rotation RoM |  |  |  |  |  |  |
|  | Stair descent | Contralateral trunk rotation RoM |  |  |  |  |  |  |
| **Other** | Stair ascent | Trunk flexion at first peak KCF |  |  |  |  |  |  |
|  | Stair ascent | Ipsilateral trunk lean at first peak KCF |  |  |  |  |  |  |
|  | Stair descent | Trunk flexion at first peak KCF |  |  |  |  |  |  |
|  | Stair descent | Ipsilateral trunk lean at first peak KCF |  |  |  |  |  |  |
|  | Step up and over task | Trunk flexion at peak total support moment |  |  |  |  |  |  |
| **Landing tasks** | | | | | | | | |
| **IC** | Drop vertical landing | Trunk flexion at IC |  |  |  |  |  |  |
|  | Single leg hop for distance | Trunk flexion at IC |  |  |  |  |  |  |
|  | Side hop | Trunk flexion at IC |  |  |  |  |  |  |
|  | Side hop | Ipsilateral trunk lean at IC |  |  |  |  |  |  |
| **Peak** | Drop vertical landing | Peak trunk flexion |  |  |  |  |  |  |
|  | Single leg drop vertical jump | Peak trunk flexion |  |  |  |  |  |  |
|  | Single leg hop for distance | Peak trunk flexion |  |  |  |  |  |  |
|  | Single leg jump cut | Peak trunk flexion |  |  |  |  |  |  |
|  | Single leg triple hop | Peak trunk flexion |  |  |  |  |  |  |
|  | Single leg vertical hop | Peak trunk flexion |  |  |  |  |  |  |
|  | Side hop | Peak ipsilateral trunk lean |  |  |  |  |  |  |
|  | Single leg drop jump | Peak ipsilateral trunk lean |  |  |  |  |  |  |
|  | Single leg drop vertical jump | Peak ipsilateral trunk lean |  |  |  |  |  |  |
|  | Single leg hop for distance | Peak ipsilateral trunk lean |  |  |  |  |  |  |
|  | Single leg hop for distance | Peak contralateral trunk lean |  |  |  |  |  |  |
| **RoM** | Bilateral drop jump | Trunk flexion RoM |  |  |  |  |  |  |
|  | Single leg drop jump | Trunk flexion RoM |  |  |  |  |  |  |
|  | Single leg hop for distance | Trunk flexion RoM |  |  |  |  |  |  |
|  | Single leg jump cut | Trunk flexion RoM |  |  |  |  |  |  |
|  | Single leg vertical countermovement jump | Trunk flexion RoM |  |  |  |  |  |  |
|  | Side hop | Ipsilateral trunk lean RoM |  |  |  |  |  |  |
|  | Single leg hop for distance | Ipsilateral trunk lean RoM |  |  |  |  |  |  |
|  | Single leg hop for distance | Contralateral trunk lean RoM |  |  |  |  |  |  |
|  | Single leg hop for distance | Trunk rotation RoM |  |  |  |  |  |  |
| **Other** | Single leg drop vertical jump | Peak trunk flexion(landing and jumping) |  |  |  |  |  |  |
|  | Single leg drop vertical jump | Trunk flexion at peak knee flexion angle |  |  |  |  |  |  |
|  | Single leg drop vertical jump | Ipsilateral trunk lean at peak knee flexion angle |  |  |  |  |  |  |
|  | Drop vertical jump | Time to peak trunk flexion |  |  |  |  |  |  |
|  | Single leg triple hop | Peak trunk flexion (1st and 2nd rebounds) |  |  |  |  |  |  |
|  | Single leg triple hop | Time to peak trunk flexion |  |  |  |  |  |  |
|  | Drop vertical jump | Time to peak ipsilateral trunk lean |  |  |  |  |  |  |
|  | Single leg triple hop | Time to peak ipsilateral trunk lean |  |  |  |  |  |  |
|  | Drop vertical jump | Time to peak ipsilateral trunk rotation |  |  |  |  |  |  |
|  | Single leg triple hop | Time to peak ipsilateral trunk rotation |  |  |  |  |  |  |
|  | Landing and cutting tasks | Two-dimensional trunk flexion |  |  |  |  |  |  |
|  | Single leg landing | Two-dimensional trunk flexion |  |  |  |  |  |  |
| **Jumping tasks** | | | | | | | | |
| **Peak** | Single leg triple hop | Peak trunk flexion |  |  |  |  |  |  |
|  | Single leg vertical hop | Peak trunk flexion |  |  |  |  |  |  |
|  | Single leg triple hop | Peak ipsilateral trunk lean |  |  |  |  |  |  |
| **RoM** | Bilateral drop jump | Trunk flexion RoM |  |  |  |  |  |  |
|  | Single leg drop jump | Trunk flexion RoM |  |  |  |  |  |  |
|  | Single leg vertical countermovement jump | Trunk flexion RoM |  |  |  |  |  |  |
| **Kinetics** | | | **Knee disorders** | | | | | |
|  |  |  | **Knee OA** | **TKA** | **PFP** | **PT** | **ACLD** | **ACLR** |
| **Running** | | | | | | | | |
| **Muscle force** | Running | Erector spinae co-contraction ratio at impact peak |  |  |  |  |  |  |
|  | Running | Erector spinae muscle force |  |  |  |  |  |  |
|  | Running | External oblique muscle force |  |  |  |  |  |  |
|  | Running | Internal oblique muscle force |  |  |  |  |  |  |
|  | Running | Rectus abdominis muscle force |  |  |  |  |  |  |
| **Landing tasks** | | | | | | | | |
| **Peak** | Single leg hop for distance | Peak trunk flexion moment |  |  |  |  |  |  |
|  | Single leg hop for distance | Peak erector spinae muscle force |  |  |  |  |  |  |
|  | Directions:  = lower with small effect; = lower with moderate effect; = lower with large effect.  = greater with small effect; = greater with moderate effect; = greater with large effect.  = no significant difference between groups.  Levels of evidence:  No evidence, gray; conflicting evidence, **black**; very limited, red; limited, orange; moderate, blue; strong, green | | | | | | | |

*IC* initial contact, *KAM* knee adduction moment, *KCF* knee contact force, *KEM* knee extensor moment, *PFJ* patellofemoral joint, *RoM* range of motion, *VGRF* vertical ground reaction force.

**Additional file 1I** Evidence gap map for waveform analyses of trunk biomechanics

| **Kinematics** | | | **Knee disorders** | | | | | |
| --- | --- | --- | --- | --- | --- | --- | --- | --- |
|  |  |  | **Knee OA** | **TKA** | **PFP** | **PT** | **ACLD** | **ACLR** |
| **Running** | | | | | | | | |
|  | Running | Trunk angle in the sagittal plane |  |  |  |  |  |  |
|  | Running | Trunk lean in the frontal plane |  |  |  |  |  | contralateral trunk lean at loading, from terminal stance through midswing, midswing and late swing |
|  | Running | Trunk rotation |  |  |  |  |  | ipsilateral trunk rotation from midstance to midswing phases |
|  | Running | Mean trunk angle in the sagittal plane |  |  |  |  |  |  |
| **Walking** | | | | | | | | |
|  | Walking | Trunk angle in the sagittal plane |  |  |  |  |  |  |
|  | Walking | Trunk lean in the frontal plane |  |  |  |  |  |  |
|  | Walking | Trunk lean in the frontal plane | trunk rotation angle during the whole stance phase |  |  |  |  |  |
|  | Walking | Mean trunk angle in the sagittal plane |  |  |  |  |  |  |
|  | Walking | Mean lateral trunk lean |  |  |  |  |  |  |
|  | Walking | Mean lateral rotation lean |  |  |  |  |  |  |
| **Squatting tasks** | | | | | | | | |
|  | Forward lunge | Trunk angle in the sagittal plane |  |  |  |  |  |  |
|  | Forward lunge | Trunk lean in the frontal plane |  |  |  |  |  |  |
|  | Forward lunge | Trunk rotation |  |  |  |  |  |  |
|  | Sideward lunge | Trunk angle in the sagittal plane |  |  |  |  |  |  |
|  | Sideward lunge | Trunk lean in the frontal plane |  |  |  |  |  |  |
|  | Sideward lunge | Trunk rotation |  |  |  |  |  |  |
|  | Single leg squat | Trunk angle in the sagittal plane |  |  |  |  |  |  |
|  | Single leg squat | Trunk lean in the frontal plane |  |  |  |  |  |  |
|  | Single leg squat | Trunk rotation |  |  |  |  |  |  |
|  | Sit-to-stand | Trunk angle in the sagittal plane |  |  |  |  |  |  |
|  | Sit-to-stand | Trunk lean in the frontal plane |  |  |  |  |  |  |
|  | Sit-to-stand | Trunk rotation |  |  |  |  |  |  |
| **Stepping tasks** | | | | | | | | |
|  | Stair ascent | Trunk angle in the sagittal plane |  |  |  |  |  |  |
|  | Stair ascent | Trunk lean in the frontal plane |  |  |  |  |  |  |
|  | Stair ascent | Trunk rotation |  |  |  |  |  |  |
|  | Stair descent | Trunk angle in the sagittal plane |  |  |  |  |  |  |
|  | Stair descent | Trunk lean in the frontal plane |  |  |  |  |  |  |
|  | Stair descent | Trunk rotation |  |  |  |  |  |  |
| **Balance tasks** | | | | | | | | |
|  | Unipodal standing | Trunk angle in the sagittal plane |  |  |  |  |  |  |
|  | Unipodal standing | Trunk lean in the frontal plane | contralateral lean in the second half of the task (~50– 100%) |  |  |  |  |  |
|  | Unipodal standing | Trunk rotation |  |  |  |  |  |  |
|  | Quite standing task | Mean trunk angle in the sagittal plane |  |  |  |  |  |  |
|  | Quite standing task | Mean lateral trunk lean |  |  |  |  |  |  |
| **Landing tasks** | | | | | | | | |
|  | lateral and medial hop | Trunk angle in the sagittal plane |  |  |  |  |  |  |
|  | Single leg hop for distance | Trunk angle in the sagittal plane |  |  |  |  |  |  |
|  | Single leg hop for distance | Mean trunk angle in the sagittal plane |  |  |  |  | flexion during the whole landing phase |  |
|  | Vertical hop w/ medial or lateral rotation | Trunk angle in the sagittal plane |  |  |  |  |  |  |
| **Jumping tasks** | | | | | | | | |
|  | Single leg triple hop | Trunk angle in the sagittal plane |  |  | flexion during the entire jumping phase |  |  |  |
|  | Single leg triple hop | Trunk lean in the frontal plane |  |  |  |  |  |  |
|  | Single leg triple hop | Trunk rotation |  |  |  |  |  |  |
| **Kinetics** | | | **Knee disorders** | | | | | |
|  |  |  | **Knee OA** | **TKA** | **PFP** | **PT** | **ACLD** | **ACLR** |
| **Landing tasks** | | | | | | | | |
|  | lateral and medial hop | Trunk flexion moment (IC - 500 milliseconds) |  |  |  |  |  |  |
|  | Single leg hop for distance | Trunk flexion moment (IC - 500 milliseconds) |  |  |  |  |  |  |
|  | Vertical hop with medial or lateral rotation | Trunk flexion moment (IC - 500 milliseconds) |  |  |  |  |  |  |
| **Jumping tasks** | | | | | | | | |
| **Induced acceleration** | Single leg triple hop | Ipsilateral erector spinae (jumping) |  |  | activity during the entire phase |  |  |  |
|  | Single leg triple hop | Ipsilateral internal oblique (jumping) |  |  | (10% and 20% of the phase) |  |  |  |
|  | Single leg triple hop | Contralateral erector spinae (jumping) |  |  | activity during the entire phase |  |  |  |
|  | Single leg triple hop | Contralateral external oblique (jumping) |  |  | (from start to 25%, and from 75% to the end) |  |  |  |
|  | Single leg triple hop | Contralateral internal oblique (jumping) |  |  | (10% and 20% of the phase) |  |  |  |
|  | Directions:  = lower;= greater; = no significant difference between groups.  Levels of certainty:  No evidence, gray; conflicting evidence, black; very limited, red; limited, orange; moderate, blue; strong, green. | | | | | | | |

*IC* initial contact.
